# Immune responses and clinical outcomes after COVID-19 vaccination in patients with liver disease and liver transplant recipients

**DOI:** 10.1016/j.jhep.2023.10.009

**Published:** 2024-01

**Authors:** Sam M. Murray, Elisa Pose, Melanie Wittner, Maria-Carlota Londoño, Golda Schaub, Jonathan Cook, Stavros Dimitriadis, Georgina Meacham, Sophie Irwin, Zixiang Lim, Paul Duengelhoef, Martina Sterneck, Ansgar W. Lohse, Valeria Perez, Palak Trivedi, Khush Bhandal, Benjamin H. Mullish, Pinelopi Manousou, Nicholas M. Provine, Emma Avitabile, Miles Carroll, Tom Tipton, Saoirse Healy, Patrizia Burra, Paul Klenerman, Susanna Dunachie, Barbara Kronsteiner, Agnieszka Katarzyna Maciola, Giulia Pasqual, Virginia Hernandez-Gea, Juan Carlos Garcia-Pagan, Pietro Lampertico, Massimo Iavarone, Pere Gines, Marc Lütgehetmann, Julian Schulze zur Wiesch, Francesco Paolo Russo, Eleanor Barnes, Thomas Marjot, the OCTAVE Collaborative Group, PITCH study, and the EASL supported COVID-Hep vaccine network

**Affiliations:** 1 Peter Medawar Building for Pathogen Research, Nuffield Department of Clinical Medicine, University of Oxford, Oxford, UK; 2 Liver Unit, Hospital Clínic, Institut de Investigacions Biomèdiques August Pi i Sunyer (IDIBAPS), University of Barcelona, Barcelona, Spain; 3 CIBERehd (Centro de Investigación Biomédica en Red Enfermedades Hepáticas y Digestivas), Spain; 4 German Center for Infection Research (DZIF), Partner Site Hamburg-Lübeck-Borstel-Riems, Germany; 5 Department of Internal Medicine, University Medical Center Hamburg-Eppendorf, Hamburg, Germany; 6 Centre for Statistics in Medicine, University of Oxford, Oxford, UK; 7 Translational Gastroenterology Unit, Nuffield Department of Medicine, University of Oxford, Oxford, UK; 8 Health Care Provider of the European Reference Network on Rare Liver Disorders (ERN-Liver), Germany; 9 National Institute for Health Research Birmingham Biomedical Research Centre, Centre for Liver and Gastrointestinal Research, Institute of Immunology and Immunotherapy, University of Birmingham, UK; 10 Liver Unit, University Hospitals Birmingham NHS Foundation Trust, Queen Elizabeth Hospital, Birmingham, UK; 11 Division of Digestive Diseases, Department of Metabolism, Digestion and Reproduction, Faculty of Medicine, Imperial College London, London, UK; 12 Department of Hepatology, St Mary’s Hospital, Imperial College Healthcare NHS Trust, London, UK; 13 Wellcome Centre for Human Genetics, University of Oxford, Oxford, UK; 14 University of Padova, Department of Surgery, Oncology and Gastroenterology DISCOG, Italy; 15 The Oxford NIHR Biomedical Research Centre, Oxford University Hospital NHS Trust, Oxford, UK; 16 Mahidol Oxford Tropical Medicine Research Unit, University of Mahidol, Bangkok, Thailand; 17 Laboratory of Synthetic Immunology, Department of Surgery, Oncology and Gastroenterology, University of Padova, Padova, Italy; 18 Veneto Institute of Oncology IOV-IRCCS, Padova, Italy; 19 Division of Gastroenterology and Hepatology, Foundation IRCCS Ca’ Granda Ospedale Maggiore Policlinico, Milan, Italy; 20 CRC “A. M. and A. Migliavacca” Center for Liver Disease, Department of Pathophysiology and Transplantation, University of Milan, Milan, Italy; 21 Institute of Medical Microbiology, Virology and Hygiene, University Medical Center Hamburg-Eppendorf, Hamburg, Germany; 22 Oxford Centre for Diabetes, Endocrinology and Metabolism (OCDEM), NIHR Oxford Biomedical Research Centre, Churchill Hospital, University of Oxford, Oxford, UK; 23 Oxford Liver Unit, Oxford University Hospitals NHS Foundation Trust, John Radcliffe Hospital, Oxford, UK

**Keywords:** COVID-19, Vaccination, Liver transplantation, Autoimmune hepatitis, Cirrhosis, Vascular liver disease, T cells, Antibodies, SARS-CoV-2, Variants of Concern

## Abstract

**Background & Aims:**

Comparative assessments of immunogenicity following different COVID-19 vaccines in patients with distinct liver diseases are lacking. SARS-CoV-2-specific T-cell and antibody responses were evaluated longitudinally after one to three vaccine doses, with long-term follow-up for COVID-19-related clinical outcomes.

**Methods:**

A total of 849 participants (355 with cirrhosis, 74 with autoimmune hepatitis [AIH], 36 with vascular liver disease [VLD], 257 liver transplant recipients [LTRs] and 127 healthy controls [HCs]) were recruited from four countries. Standardised immune assays were performed pre and post three vaccine doses (V1-3).

**Results:**

In the total cohort, there were incremental increases in antibody titres after each vaccine dose (p <0.0001). Factors associated with reduced antibody responses were age and LT, whereas heterologous vaccination, prior COVID-19 and mRNA platforms were associated with greater responses. Although antibody titres decreased between post-V2 and pre-V3 (p = 0.012), patients with AIH, VLD, and cirrhosis had equivalent antibody responses to HCs post-V3. LTRs had lower and more heterogenous antibody titres than other groups, including post-V3 where 9% had no detectable antibodies; this was heavily influenced by intensity of immunosuppression. Vaccination increased T-cell IFNγ responses in all groups except LTRs. Patients with liver disease had lower functional antibody responses against nine Omicron subvariants and reduced T-cell responses to Omicron BA.1-specific peptides compared to wild-type. 122 cases of breakthrough COVID-19 were reported of which 5/122 (4%) were severe. Of the severe cases, 4/5 (80%) occurred in LTRs and 2/5 (40%) had no serological response post-V2.

**Conclusion:**

After three COVID-19 vaccines, patients with liver disease generally develop robust antibody and T-cell responses to vaccination and have mild COVID-19. However, LTRs have sustained no/low antibody titres and appear most vulnerable to severe disease.

**Impact and implications:**

Standardised assessments of the immune response to different COVID-19 vaccines in patients with liver disease are lacking. We performed antibody and T-cell assays at multiple timepoints following up to three vaccine doses in a large cohort of patients with a range of liver conditions. Overall, the three most widely available vaccine platforms were immunogenic and appeared to protect against severe breakthrough COVID-19. This will provide reassurance to patients with chronic liver disease who were deemed at high risk of severe COVID-19 during the pre-vaccination era, however, liver transplant recipients had the lowest antibody titres and remained vulnerable to severe breakthrough infection. We also characterise the immune response to multiple SARS-CoV-2 variants and describe the interaction between disease type, severity, and vaccine platform. These insights may prove useful in the event of future viral infections which also require rapid vaccine development and delivery to patients with liver disease.

## Highlights

• Factors associated with reduced antibody responses were age and previous LT.
• Heterologous vaccination, prior COVID-19, and mRNA platforms were associated with greater responses.
• Patients with liver disease had lower T-cell and functional antibody responses against Omicron subvariants compared to wild-type.
• LTRs had lower antibody responses than all other groups with titres heavily influenced by intensity of immunosuppression.
• 122 cases of breakthrough COVID-19 were reported; only 5/122 (4%) were severe and 4/5 (80%) of these severe cases occurred in LTRs.

## Graphical abstract

**Figure:**
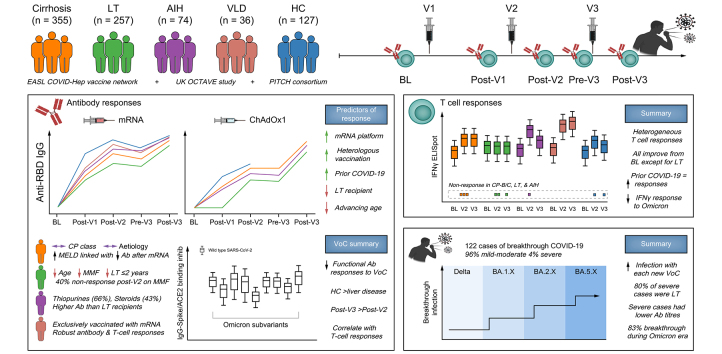

## Introduction

The rapid development and deployment of vaccinations against SARS-CoV-2, alongside a degree of naturally acquired immunity from past infection, has transformed the landscape of the COVID-19 pandemic. At a population level, vaccination has been shown to reduce SARS-CoV-2 infection and protect against hospitalisation and death from severe COVID-19.^1^^,^^2^ However, understanding the immunogenicity and effectiveness of vaccination programmes in vulnerable cohorts with functional or pharmacological immunosuppression remains an important clinical priority.^3^ This is particularly relevant given the continuing emergence of novel viral variants of concern (VoC). In the pre-vaccination era, patients with a range of liver conditions were shown to be at increased risk of SARS-CoV-2 infection and severe COVID-19. This included high rates of mortality in cohorts with cirrhosis, greater intensive care unit requirements in liver transplant recipients (LTR), and an elevated risk of SARS-CoV-2 infection and severe complications in patients with vascular liver disease (VLD).[4], [5], [6], [7] Epidemiological studies have since demonstrated a benefit of COVID-19 vaccination in some of these groups. This includes retrospective analysis of electronic health record data showing that three doses of mRNA vaccination are associated with a significant reduction in rates of SARS-CoV-2 infection and severe COVID-19 in patients with cirrhosis.^8^ In addition, there have been several studies exploring immune responses to vaccination in liver cohorts.[9], [10], [11] However, these studies have often examined responses after only one or two vaccine doses and overall conclusions have been hampered by small sample sizes, differing sampling timepoints, cross-sectional design, heterogeneity in laboratory assays, absence of VoC analysis, and lack of healthy control datasets. As a result, considerable uncertainty remains within the literature regarding vaccine immunogenicity across the various vaccine platforms and liver disease phenotypes.^10^ Data relating to T-cell responses after vaccination are also lacking, except for some small, detailed studies in individual liver groups.^12^ This requires further investigation in patients with liver disease given that cellular responses are an important correlate of protection against severe COVID-19.^13^^,^^14^ Lastly, to date, studies have tended to be single-centre and have interrogated solitary vaccine types which limits their widespread generalisability.

As a result, we sought to deliver a European multicentre prospective cohort study evaluating T-cell and antibody responses following COVID-19 vaccination in LTRs, patients with cirrhosis, autoimmune hepatitis (AIH), or VLD, and healthy controls (HCs). Herein, we performed longitudinal immunological assessments in these groups using standardised assays at multiple timepoints following up to three doses of differing COVID-19 vaccine regimens. It also involved assessments of vaccine responses to some of the most up-to-date viral variants including multiple sublineages of Omicron. In addition, patients were followed long-term to establish the occurrence and severity of breakthrough SARS-CoV-2 infection after vaccination.

## Patients and methods

### Study design and sampling protocol

We evaluated the humoral and cellular immune response after COVID-19 vaccination delivered as part of clinical care to European patients with cirrhosis, AIH, or VLD, or LTRs. Patients were prospectively recruited across two multicentre consortia; the EASL supported COVID-Hep vaccine network and the UK OCTAVE (Observational Cohort trial T-cells, Antibodies and Vaccine Efficacy in SARS-CoV-2) study. Both studies had identical inclusion criteria; >16 years of age, eligibility for COVID-19 vaccination with BNT162b2, mRNA-1273, or ChAdOx1 nCoV-19 (AZD1222, ChAdOx1) vaccine platforms, and an anticipated life expectancy >6 months. Healthy controls were recruited via the UK PITCH (Protective Immunity from T Cells in Healthcare workers) consortium. All studies included clinical and demographic data collection at baseline followed by longitudinal collection of serum and peripheral blood mononuclear cells (PBMCs) at standardised timepoints throughout the vaccination schedule, although sample availability at each timepoint was variable. A subset of data included in this work has been previously reported.[14], [15], [16] However, these studies included: i) patients who had received a single vaccine type such that comparisons of immunogenicity and vaccine effectiveness between vaccine types could not be made,^16^ ii) patients who received only two vaccine doses and no booster vaccines^14^^,^^15^ and iii) did not include any data on SARS-CoV-2 infection rates and disease severity data.^15^^,^^16^

### Ethical and regulatory approvals

All centres involved in the EASL supported COVID-Hep vaccine registry recruited participants through local ethics approvals. All studies were conducted in compliance with relevant ethical regulations for work with human participants according to the principles of the Declaration of Helsinki (2008) and written informed consent was obtained for all included participants. Additional methods are available in the supplementary materials.

### Outcome measures and sample size calculations

The two primary outcomes of this study were levels of anti-SARS-CoV-2 IgG antibodies and the magnitude of the T-cell responses to wild-type (WT) SARS-CoV-2 peptides following COVID-19 vaccination. The study was powered to detect a difference in SARS-CoV-2 IgG anti-S antibody titres 28 days after V2 between disease cohorts (cirrhosis, AIH, VLD, LTR) and HCs. Sample size calculations were performed *a priori* based on available data at the time of study conception relating to COVID-19 vaccine immunogenicity in phase III trials and historic data showing diminished vaccine responses to a range of other viruses in LTRs and patients with cirrhosis. To allow for comparisons between vaccine platforms and assuming a 20% reduction in antibody titres in patients relative to controls we estimated that at least 100 patients per disease group would be required to detect a difference with 90% power and an alpha of 0.05. Secondary outcomes included the magnitude of the T-cell responses specifically to SARS-CoV-2 Omicron BA.1 peptides, IgG binding and inhibition of angiotensin-converting enzyme 2 (ACE2) binding to SARS-CoV-2 VoC, and rates and severity of breakthrough SARS-CoV-2 infection after COVID-19 vaccination.

### Clinical phenotyping and definitions

Clinical data for all participants were uploaded electronically from participating sites using REDCap (Research Electronic Data Capture) databases hosted by the University of Oxford or University of Birmingham, UK. Clinical data comprised information on demographics, vaccination type, comorbidities, and disease-specific phenotyping including Child-Pugh (CP) class and aetiology of cirrhosis, and type and dose of immunosuppression for patients with AIH and LTRs. Previous SARS-CoV-2 infection was defined as a patient-reported episode of confirmed COVID-19 or a positive antibody or T-cell response to SARS-CoV-2 nucleocapsid antigen at the first timepoint at which a patient was sampled. Nucleocapsid antibody titres were additionally assessed at post-V1, and post-V3 timepoints. Model for end-stage liver disease (MELD) score included serum sodium, creatinine, bilirubin and international normalised ratio.^17^

### Anti-SARS-CoV-2 Ig analysis

For all participants, the magnitude of anti-SARS-CoV-2 antibodies was measured using identical Roche Elecsys® Anti-SARS-CoV-2-S (anti-S) and Roche Elecsys® Anti-SARS-CoV-2-N (anti-N) assays. The Roche Anti-SARS-CoV-2-S assay measures the presence and the amount of serum antibodies to the spike receptor binding domain (RBD) antigen of SARS-CoV-2. Seroconversion was manufacturer defined as anti-S antibodies ≥0.8 U/ml and anti-N antibodies >1U/ml. The upper limit of detection for anti-S antibodies was 25,000 U/ml. Low response was defined as <380 U/ml as per.^14^ Assays for all samples were completed at the University of Hamburg or the UKHSA Laboratories at Porton Down.

### Anti-SARS-CoV-2 VoC IgG binding and ACE2 inhibition

IgG titres and ACE2 inhibition were measured against the spike or RBD of WT SARS-CoV-2 and the nine most prevalent Omicron subvariants as of February 2023 (B.1.1.529/BA.1/BA.1.15, BA.2.75, BA.2.75.2, BA.4.6, BA.5, BF.7, BQ.1, BQ.1.1, and XBB.1), using multiplexed Meso Scale Discovery immunoassay panels 32 and 33 (K15668U and K15679U). Assays were performed as per manufacturer recommendations in a subset of patients and HCs, who were selected to include a range of post-V2 anti-RBD Ig titres. Additional methods are available in the supplementary materials.

### IFNγ T-cell ELISpot assay

IFNγ ELISpots were performed using Human IFNγ ELISpot Basic kit (Mabtech) as previously described.^18^ Wells included 200,000 thawed PBMCs and stimulation conditions included overlapping peptide pools (18 mers with 10 amino acid overlap), negative control (DMSO only) or positive control (CEF and concanavalin A). IFNγ ELISpots for liver disease groups were all performed at one laboratory at the University of Oxford. IFNy ELISpots for healthy controls were performed separately at the University of Oxford. Samples were only included in the analysis if the IFNγ response to DMSO was <50 spot-forming units (SFU)/10^6^ PBMCs and positive responses were detected in the positive controls. Additional methods are available in the supplementary materials.

### Breakthrough SARS-CoV-2 infection after COVID-19 vaccination

Information regarding breakthrough SARS-CoV-2 infection after vaccination was also collected by screening electronic hospital records and/or contacting individual patients and included date of infection and COVID-19 severity. Data was collected up until December 2022. Severe COVID-19 was defined according to the World Health Organisation classification or based on hospitalisation status.^19^ Additional methods are available in the supplementary materials.

### Statistical methods

Descriptive statistics are presented as n (%) or median (IQR), unless otherwise indicated. A nominal 2-sided 5% significance level was adopted unless otherwise stated. Kruskal-Wallis test followed by *post hoc* Dunn’s testing with Holm-Bonferroni multiplicity adjustment was used for multiple pairwise comparisons. Mann-Whitney *U* test, Wilcoxon matched-paired signed rank test, Pearson’s Chi-Square, and Fisher’s exact test were used where required as per figure legends. Multiple comparisons corrections were made as per individual figure legends (Holm-Bonferroni adjustments). Spearman’s correlations or linear regressions of log^10^ transformed values investigated relationships between variables of interest. Spearman’s correlations were used to quantify the association between variables and linear regression models were used to model effects of clinical or immunological factors on anti-RBD Ig responses. Multivariable models (linear or logistic as per legends) were used to model multivariate effects of clinical and immunological factors on log^10^ transformed anti-RBD Ig responses (linear) or odds ratio of seropositivity (>0.8 AU/ml, logistic) at post-v2 in the whole cohort or in specific disease groups. Models were created as per figure legends. Statistical analyses were performed using R (v4.2.1) or in GraphPad Prism (v9.4.0). Figures were prepared using R (v4.2.2 (2022-10-31)) with RStudio 2023.03.1+446 or in GraphPad Prism (v9.4.0). R packages used included: ggplot2, rstatix, Hmisc, gtsummary, lme4.

## Results

### Cohort characteristics

Between March and September 2021, a total of 849 individuals (722 patients with liver disease and 127 HCs) were prospectively recruited from the UK, Italy, Germany, and Spain. The liver disease patient cohort comprised 355 (49%) individuals with cirrhosis, 257 (36%) LTRs, 74 (10%) individuals with AIH, and 36 (5%) with VLD. In the entire cohort, the primary two-dose vaccination course included ChAdOx1 (n = 246), BNT162b2 (n = 460), and mRNA-1273 (n = 118). An additional 13 individuals received heterologous vaccination with ChAdOx1 for first vaccination followed by an mRNA platform (BNT162b2 or mRNA-1273) for the second vaccination. Data were available for 307 participants after a third vaccination which included 187 (61%) receiving BNT162b2, 110 (36%) receiving mRNA-1273, and 10 (3%) where the vaccine type was unknown.

Across the entire liver disease cohort, the median age was 60 years (IQR 52–68), 425 (59%) were male, and 76 (11%) had previous evidence of SARS-CoV-2 infection. In the HC cohort, the median age was 36 years (IQR 25–45), 40 (31%) were male, and 40 (31%) had evidence of previous SARS-CoV-2 infection. Nineteen patients became newly positive for nucleocapsid antibody at the post-V3 timepoint. Clinical information for each specific disease category is presented in Table 1. For the analysis, 21 (29%) patients with AIH and concurrent cirrhosis were included in the AIH group and not the cirrhosis group.

**Table 1:** Clinical characteristics.

	LT (n = 257)	AIH (n = 74)	Cirr (n = 355)	VLD (n = 36)	HC (n = 127)	Total (N = 849)
Age (years, IQR)	60 (50-67)	61 (49-69)	62 (55-69)	46 (40-49)	36 (25-45)	58 (46-66)
Unknown	2 (0.8%)	0 (0%)	2 (0.6%)	0 (0%)	1 (0.8%)	5 (0.5%)
Sex						
Female	98 (38%)	61 (82%)	123 (35%)	15 (42%)	86 (68%)	383 (45%)
Male	159 (62%)	13 (18%)	232 (65%)	21 (58%)	40 (31%)	465 (55%)
Unknown	0 (0%)	0 (0%)	0 (0%)	0 (0%)	1 (0.8%)	1 (0.1%)
Ethnicity						
Asian	1 (0.4%)	5 (6.8%)	7 (2.0%)	0 (0%)	16 (13%)	29 (3.4%)
Black	2 (0.8%)	0 (0%)	8 (2.3%)	1 (2.8%)	1 (0.8%)	12 (1.4%)
Other	3 (1.2%)	2 (2.7%)	7 (2.0%)	4 (11%)	4 (3.1%)	20 (2.4%)
White	132 (51%)	65 (88%)	293 (83%)	31 (86%)	84 (66%)	605 (71%)
Unknown	119 (46%)	2 (2.7%)	40 (11%)	0 (0%)	22 (17%)	183 (22%)
Obesity						
No	156 (61%)	51 (69%)	220 (62%)	31 (86%)	82 (65%)	540 (64%)
Yes	49 (19%)	14 (19%)	116 (33%)	4 (11%)	5 (3.9%)	188 (22%)
Unknown	52 (20%)	9 (12%)	19 (5.4%)	1 (2.8%)	40 (31%)	121 (14%)
Smoking status						
Never smoked	227 (88%)	44 (59%)	188 (53%)	25 (69%)	117 (92%)	601 (71%)
Previously smoked	28 (11%)	22 (30%)	107 (30%)	2 (5.6%)	10 (7.9%)	169 (20%)
Currently smoke	2 (0.8%)	8 (11%)	60 (17%)	9 (25%)	0 (0%)	79 (9.3%)
Diabetes						
Yes	25 (9.7%)	10 (14%)	102 (29%)	2 (5.6%)	0 (0%)	139 (17%)
Hypertension						
Yes	87 (34%)	12 (16%)	136 (38%)	0 (0%)	3 (2.9%)	238 (29%)
Prior SARS-CoV-2 infection						
No confirmed infection	241 (94%)	68 (92%)	307 (86%)	30 (83%)	87 (69%)	733 (86%)
Previously infected	16 (6.2%)	6 (8.2%)	48 (14%)	6 (17%)	40 (31%)	116 (14%)
Vaccine type - dose 1						
ChAdOx1 nCoV-19	73 (28%)	38 (51%)	112 (32%)	0 (0%)	39 (31%)	262 (31%)
BNT162b2	175 (68%)	23 (31%)	176 (50%)	0 (0%)	87 (69%)	461 (54%)
mRNA-1273	7 (2.7%)	13 (18%)	64 (18%)	36 (100%)	1 (0.8%)	121 (14%)
Unknown	2 (0.8%)	0 (0%)	3 (0.8%)	0 (0%)	0 (0%)	5 (0.6%)
Vaccine type - dose 2						
ChAdOx1 nCoV-19	66 (26%)	37 (50%)	104 (29%)	0 (0%)	39 (31%)	246 (29%)
BNT162b2	180 (70%)	22 (30%)	180 (51%)	0 (0%)	87 (69%)	469 (55%)
mRNA-1273	9 (3.5%)	14 (19%)	64 (18%)	33 (92%)	1 (0.8%)	121 (14%)
Unknown	2 (0.8%)	1 (1.4%)	7 (2.0%)	3 (8.3%)	0 (0%)	13 (1.5%)
Vaccine type - dose 3∗						
BNT162b2	88 (85%)	4 (24%)	70 (52%)	0 (0%)	25 (89%)	187 (61%)
mRNA-1273	9 (8.7%)	13 (76%)	61 (46%)	24 (100%)	3 (11%)	110 (36%)
Unknown	7 (6.7%)	0 (0%)	3 (2.2%)	0 (0%)	0 (0%)	10 (3.3%)
<2 years post LT						
Yes	37 (14%)	—	—	—	—	—
Indication for LT						
Acute liver failure	21 (8%)	—	—	—	—	—
Decompensated cirrhosis	99 (39%)	—	—	—	—	—
HCC	33 (13%)	—	—	—	—	—
Other	40 (16%)	—	—	—	—	—
Unknown	64 (25%)	—	—	—	—	—
IS						
Azathioprine	16 (6.2%)	21 (29%)	2 (0.6%)	—	—	—
Sirolimus	6 (2.3%)	0 (0%)	—	—	—	—
Everolimus	44 (17%)	0 (0%)	—	—	—	—
6-MP	5 (1.9%)	27 (37%)	1 (0.3%)	—	—	—
MMF	79 (31%)	9 (12%)	1 (0.3%)	1 (3%)	—	—
MTX	—	—	2 (0.6%)	—	—	—
Corticosteroids	50 (19%)	33 (43%)	1 (0.3%)	1 (3%)	—	—
Ciclosporin	41 (16%)	0 (0%)	—	—	—	—
Tacrolimus	195 (76%)	6 (8.2%)	—	—	—	—
No. IS therapies						
0	3 (1.2%)	8 (11%)	—	—	—	—
1	95 (37%)	36 (49%)	—	—	—	—
2	132 (51%)	23 (32%)	—	—	—	—
3	27 (11%)	6 (8.2%)	—	—	—	—
IS combinations						
CNI only	81 (32%)	—	—	—	—	—
mTori only	13 (5.1%)	—	—	—	—	—
CNI + MMF (+/- other)	79 (31%)	—	—	—	—	—
CNI + other (+/- other)	81 (32%)	—	—	—	—	—
Cirrhosis severity						
MELD score (median, IQR)	—	—	7.25 (6.67, 8.43)	—	—	—
Unknown	—	—	60 (17%)	—	—	—
Child-Pugh class						
A	—	19 (26%)	232 (65%)	—	—	—
B	—	2 (2.7%)	82 (23%)	—	—	—
C	—	1 (1.4%)	31 (8.7%)	—	—	—
No cirrhosis	—	52 (70%)	0 (0%)	—	—	—
Unknown	—	—	10 (2.8%)	—	—	—
Cirrhosis aetiology						
NAFLD	—	1 (5%)	98 (28%)	—	—	—
ALD	—	1 (5%)	152 (43%)	—	—	—
HCV	—	2 (9%)	80 (22%)	—	—	—
HBV	—	1 (5%)	24 (6.7%)	—	—	—
PBC	—	2 (9%)	12 (2.8%)	—	—	—
PSC	—	2 (9%)	12 (3.1%)	—	—	—
Unknown	—	15 (68%)	33 (9.3%)	—	—	—
VLD aetiology						
NCPVT	—	—	—	16 (44%)	—	—
BCS	—	—	—	9 (25%)	—	—
PSVD	—	—	—	11 (31%)	—	—

6-MP, 6-mercaptopurine; AIH, autoimmune hepatitis; ALD, alcohol-related liver disease; BCS, Budd-Chiari syndrome; Cirr, cirrhosis; CNI, calcineurin inhibitor; HCs, healthy controls; HCC, hepatocellular carcinoma; IS, immunosuppression; LT, liver transplant; MMF, mycophenolate mofetil; mTORi, mTOR inhibitor; MTX, methotrexate; NAFLD, non-alcoholic fatty liver disease; NCPVT, non-cirrhotic non-tumoral portal vein thrombosis; PBC, primary biliary cholangitis; PSC, primary sclerosing cholangitis; PSVD, porto-sinusoidal vascular disorder; VLD, vascular liver disease. ∗Numbers only given for individuals with third dose anti-RBD Ig titre data available.

### Antibody responses

#### Antibody responses across liver disease phenotypes and vaccine platforms

**Fig. 1:**
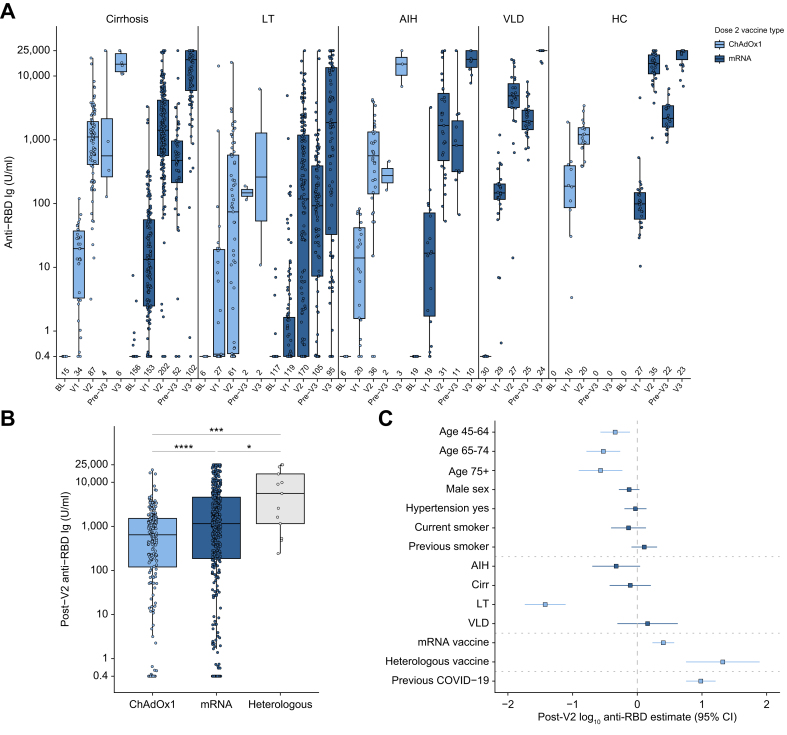
COVID-19 vaccine-induced anti-SARS-CoV-2 RBD total Ig in SARS-CoV-2-naïve individuals. (A) Assessment of COVID-19 vaccine responses at baseline (BL), post first vaccine (V1), 28 days after second vaccine (V2), immediately prior to third vaccine (pre-V3) and 28 days after third vaccine (V3). All participants had an mRNA vaccine as their third dose, individuals vaccinated with a heterologous vaccine regimen were excluded. (B) Comparison of vaccination platforms at the post-V2 timepoint. (C) Forest plot depicting results from multivariable linear regression model of post-V2 log10 transformed anti-SARS-CoV-2 RBD Ig in all participants. (A,B) Boxes represent median and IQR, whiskers represent +/- 1.5x IQR. (C) Point represents odds ratio, whiskers 95% CI. (B) Mann-Whitney *U* test with Holm-Bonferroni adjustment. Dark blue indicates significantly predictive variables (*p* < 0.05). ∗*p* < 0.05, ∗∗∗*p* < 0.001. AIH, autoimmune hepatitis; Cirr, cirrhosis; HCs, healthy controls; LT, liver transplant; RBD, receptor binding domain; VLD, vascular liver disease.

Longitudinal assessment of antibody responses using the Roche anti-RBD assay across all liver disease phenotypes and HCs is presented in Fig. 1A. Prior SARS-CoV-2 infection was associated with a significant increase in anti-RBD titres across all disease groups (Fig. S1) and therefore previously infected individuals were removed from the primary analysis (n = 76 with liver disease and n = 40 HCs excluded).

Across the total liver disease cohort, there was a stepwise incremental increase in median antibody titres after each consecutive vaccine dose (6.24 U/ml [0.4–44.9] post-V1 *vs.* 846 U/ml [158–2,653] post-V2 *vs.* 12,746 U/ml [2,508–25,000] U/ml post-V3; *p* < 0.0001). This observation remained significant after excluding 19 patients who had become newly positive for nucleocapsid antibody between enrolment and post-V3 (Fig. S1B). A decrease in antibody titre between the post-V2 and pre-V3 timepoint was also observed in both liver disease and HC groups and was subsequently boosted by the administration of a third vaccine dose (Table S1). mRNA platforms were associated with significantly higher post-V2 anti-RBD titres compared to ChAdOx1 in both liver disease (953 [158–3,214] mRNA *vs.* 593 U/ml [114–1521] ChAdOx1; *p =* 0.0005) and HC cohorts (15,634 U/ml [10,829–21,445] mRNA *vs.* 1,198 U/ml [855–1,546] ChAdOx1; *p* < 0.001). Thirteen patients who received heterologous first and second vaccines had significant elevations in post-V2 antibody titres compared to homologous ChAdOx1 (*p =* 0.0002) and homologous mRNA regimens (*p =* 0.004) (Fig. 1B). In cohorts where data were available for both BNT162b2 or mRNA-1273 vaccinated individuals, there were no significant differences in anti-RBD at the post-V2 timepoint; therefore, both mRNA vaccines were grouped for further analysis (Fig. S2). Within the entire study cohort (patients and HCs) multivariable analyses showed that the factors significantly associated with lower antibody response after V2 were advancing age and inclusion in the LTR group, and factors associated with greater response were mRNA vaccination, heterologous vaccination (V1 + V2) and previous COVID-19 infection (Fig. 1C, Table S2).

Our study allowed us to compare antibody responses relating to specific combinations of disease and vaccine types. At the post-V2 and post-V3 timepoints LTRs mounted lower antibody titres compared to all other disease groups and HCs regardless of vaccine type (Table S3). mRNA-vaccinated patients with cirrhosis had reduced post-V2 antibody titres compared to mRNA-vaccinated HCs, but ChAdOx1-vaccinated patients with cirrhosis had comparable post-V2 titres to ChAdOx1-vaccinated HCs.

We observed variable non-response rates across disease groups depending on vaccine type and timepoint (Table S4). No HCs were seronegative after either two or three vaccine doses. LTRs had the highest rates of serological non-response with 18/52 (35%) and 52/179 (29%) having absent responses after two doses of ChAdOx1 and mRNA vaccines, respectively. Non-response rates were reduced at the post-V3 timepoint in all disease groups, with only 9/97 (9%) LTRs and 2/108 (2%) patients with cirrhosis not having a serological response to vaccine at the post-V3 timepoint.

#### Serological cross-reactivity of SARS-CoV-2 VoC

**Fig. 2:**
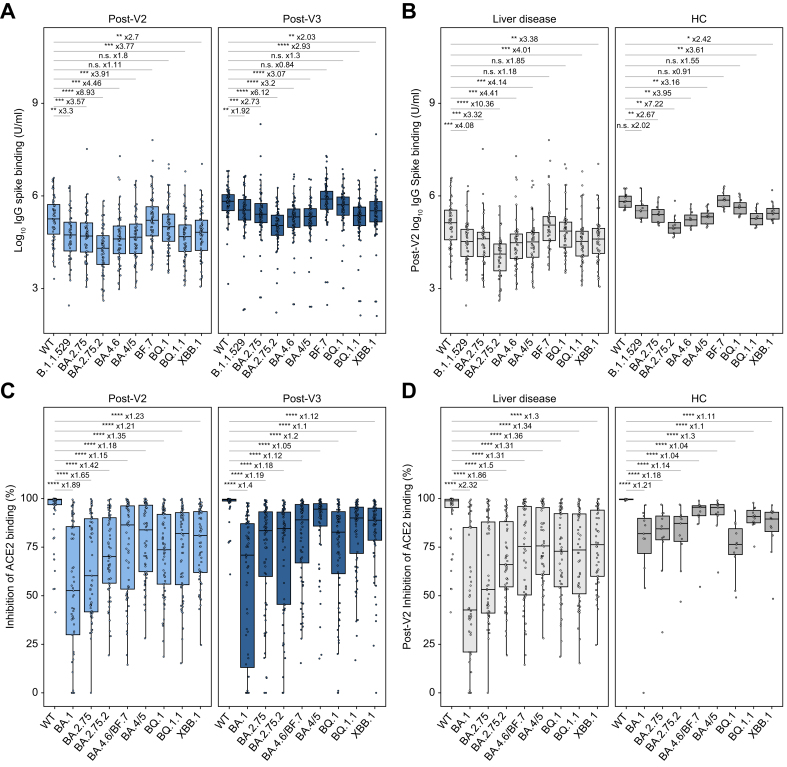
COVID-19 vaccine-induced serological responses to Omicron subvariants. (A,B) IgG to subvariant spike protein and (C,D) inhibition of ACE2 binding to subvariant RBD in 68 individuals. (A,C) Includes all individuals split by timepoint and (B,D) are post-V2 responses separated by liver disease (inc. liver transplant) and HCs. Mann-Whitney *U* test with Holm-Bonferroni adjustment. Boxes represent median and IQR, whiskers represent +/- 1.5x IQR. Fold-change of median depicted. ∗*p* < 0.05, ∗∗*p* < 0.01, ∗∗∗*p* < 0.001, ∗∗∗∗*p* <0.0001. ACE2, angiotensin-converting enzyme 2; HCs, healthy controls; RBD, receptor binding domain; WT, wild-type.

IgG binding to the spike protein of SARS-CoV-2 Omicron subvariants was assessed at post-V2 and post-V3 timepoints in selected samples from liver disease and HC cohorts (Fig. 2A). Within the combined cohort (liver disease and HCs) at both timepoints, serological titres to all Omicron subvariants (except for BF.7 and BQ.1) were significantly lower compared to WT SARS-CoV-2. The subvariants with the greatest decrease in IgG binding compared to WT were BA2.75.2 (median fold decrease: x8.93 post-V2 and x6.12 post-V3) and BA.4.6 (x4.46 post-V2 and x3.2 post-V3) which were both first identified in autumn 2022. Notably, the magnitude of reduction in IgG binding to Omicron subvariants relative to WT was lower following V3 compared to post-V2. The same trends were observed when IgG binding was split by liver disease aetiology (Fig. 2B and Fig. S3A). However, HCs had less of a decrease in IgG binding to Omicron subvariants relative to WT than seen in patients with liver disease. Serum from most participants inhibited ACE2 binding to WT RBD at post-V2 and post-V3 (Fig. 2C), whereas there was a significant decrease in inhibition across all Omicron subvariants relative to WT. Again, the decrease in ACE2 binding post-V3 was less pronounced compared to post-V2. There was also less of a decrease at the post-V2 and post-V3 timepoints in HCs compared to patients with liver disease (Figs 2D and S3B). The ratio of IgG binding to WT spike compared to VoC spike was significantly increased by a third vaccine in the liver disease group, but no change was observed between two and three vaccine responses in HCs (Fig. S3C). We observed significant positive correlations at post-V2 and post-V3 timepoints when comparing the Roche anti-RBD titre with VoC binding IgG, ACE2 inhibition, and with the ratio between WT to VoC binding IgG (Fig. S4A-C).

#### Liver transplant recipients

**Fig. 3:**
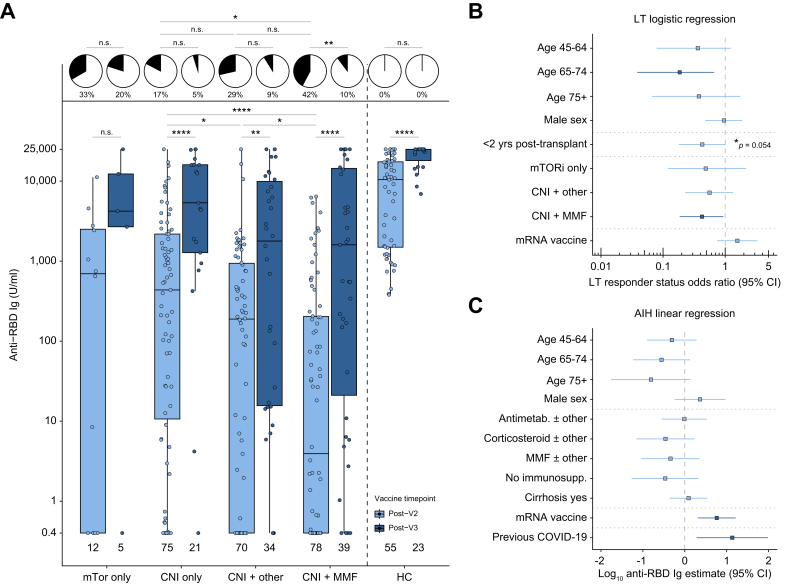
Serological responses to COVID-19 vaccines in immunosuppressed patients with LT and AIH. (A) Magnitude of anti-SARS-CoV-2 RBD Ig in SARS-CoV-2 infection-naïve liver transplant recipients and HCs. Proportions of seropositive (white) and seronegative (black) (<0.8 U/ml) patients in each subgroup presented in pie charts. (B) Forest plot of multivariable logistic regression showing odds of seropositivity at the post-V2 timepoint within the liver transplant cohort and (C) results of multivariable linear regression of log10 anti-SARS-CoV-2 RBD Ig at post-v2 timepoint in autoimmune hepatitis cohort. (A) Boxes represent median and IQR, whiskers represent +/- 1.5x IQR. (B,C) Point represents odds ratio, whiskers 95% CI. (A) Mann-Whitney *U* test or Fisher’s exact tests with Holm-Bonferroni adjustment. Dark blue indicates significantly associated variables (*p* < 0.05). n.s., non-significant, ∗*p* < 0.05, ∗∗*p* < 0.01, ∗∗∗*p* < 0.001, ∗∗∗∗*p* < 0.0001. AIH, autoimmune hepatitis; Antimetab, antimetabolite immunosuppression; CNI, calcineurin inhibitor; CNI + other, calcineurin inhibitor plus any immunosuppression other than MMF; HC, healthy control; LT, liver transplant; MMF, mycophenolate mofetil; mTORi, mTOR inhibitor; RBD, receptor binding domain.

Antibody responses to two and three vaccine doses in LTRs are presented in Fig. 3A and are separated according to class of immunosuppression. This shows a downward trend in post-V2 anti-RBD titres associated with increasing intensity of immunosuppression, with significant reductions observed in patients on a calcineurin inhibitor (CNI) plus mycophenolate mofetil (MMF) *vs*. a CNI alone, and in patients on a CNI plus another immunosuppressant other than MMF, compared to CNI alone. LTRs on MMF additionally had significantly reduced responses compared to those on thiopurines (*p =* 0.011, Fig. S5A), and an increasing daily dose of MMF was significantly associated with decreased anti-RBD titres at the post-V2 timepoint (*p =* 0.031) (Fig. S5B). A third vaccine dose significantly improved antibody responses across all groups, except with mTOR inhibitor monotherapy where cohort numbers were small (Fig. 3A). LTRs had high rates of antibody non-response, with non-response rates at the post-V2 timepoint of 4/12 (33%) in the mTOR inhibitor only group, 13/75 (17%) in the CNI only group, 20/70 (29%) in the CNI plus other immunosuppression group and 33/78 (42%) in the CNI plus MMF group. The rate of serological non-responsiveness was significantly higher in the CNI plus MMF group compared to the CNI alone group. A third vaccine dose led to improvement in rates of non-response across all subgroups of immunosuppression (mTOR inhibitor only 1/5 [20%]; CNI only 1/21 [5%]; CNI plus other immunosuppression 3/34 [9%]; CNI plus MMF 4/39 [10%]).

Among LTRs, both univariable and multivariable analyses showed that the factors significantly associated with reduced odds of seropositivity after V2 were age (65-74 age group) and CNI plus MMF (Fig. 3B, Table S5). LTRs with previous SARS-CoV-2 infection were removed from the logistic regression, as all of these patients had a detectable response after V2.

#### Patients with autoimmune hepatitis

Patients with AIH had higher post-V2 antibody responses than LTRs with both ChAdOx1 and mRNA platforms despite both groups being immunosuppressed and being of similar age (61 years [49–69] *vs.* 60 years [52–68]). Unlike LTRs, there were no significant differences in serological response between class and intensity of immunosuppression at post-V2, despite the fact some patients with AIH (6/8 with MMF dose data) were on high dose (2 g/day) MMF (Fig. S6A). There were also no differences in response when the AIH cohort was split by the presence or absence of cirrhosis (Fig. S6B). Univariable and multivariable analyses showed that the factors significantly associated with higher antibody responses after V2 were mRNA vaccination and previous COVID-19 (Fig. 3C, Table S6).

#### Patients with cirrhosis

**Fig. 4:**
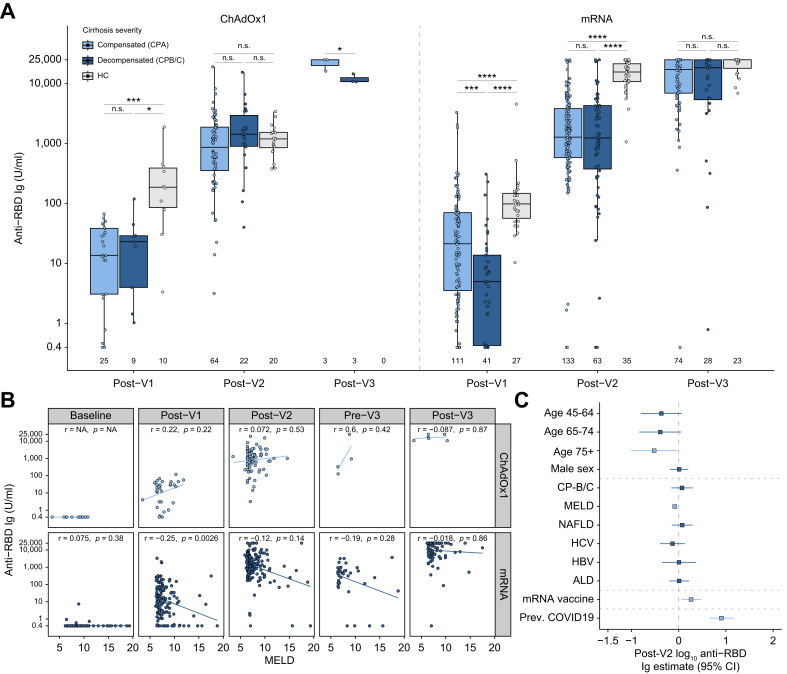
Serological responses to COVID-19 vaccines in patients with cirrhosis. (A) Magnitude of vaccine responses in SARS-CoV-2 infection-naïve patients with cirrhosis and healthy controls. Vaccine type is for first two vaccine doses. (B) Spearman correlation of MELD score with anti-SARS-CoV-2 RBD Ig in same cirrhosis patients as A. (C) Forest plot depicting results of multivariable linear regression of log10 anti-SARS-CoV-2 RBD Ig at post-V2 timepoint in cirrhosis cohort. (A) Boxes represent median and IQR, whiskers represent +/- 1.5x IQR. (B) Line represents linear regression. (C) Point represents odds ratio, whiskers 95% CI. (A) Kruskal-Wallis with *post hoc* Dunn’s test adjusted with Holm-Bonferroni method. (C) Dark blue indicates significantly associated variables (*p* <0.05). n.s., non-significant, ∗∗*p* < 0.01, ∗∗∗*p* < 0.001, ∗∗∗∗*p* < 0.0001. ALD, alcohol-related liver disease; CP, Child-Pugh class; HC, healthy control; NALFD, non-alcoholic fatty liver disease.

Antibody responses in patients with cirrhosis at post-V1, post-V2, and post-V3 are presented in Fig. 4A and are separated according to vaccination type and CP class. This revealed a dynamic interaction between number of doses, severity of cirrhosis, and vaccine type. At post-V1 there were no differences in antibody titres between compensated (CP-A) and decompensated (CP–B/C) cirrhosis when vaccinated with ChAdOx1. However, CP-A did have higher antibodies than CP-B/C at post-V1 when vaccinated with mRNA. At post-V1, both CP-A and CP-B/C had lower antibody titres than HC with both vaccine types. At post-V2, there were no significant differences between CP-A and CP-B/C or between patients and HCs when vaccinated with ChAdOx1. Whereas at post-V2 for mRNA, HCs had higher titres than patients with cirrhosis irrespective of CP class. At post-V3, there were no significant differences between any groups. Broadly, this suggested an association between liver disease severity and reduced antibody responses when vaccinated with mRNA but not the ChAdOx1 platform, particularly early in the vaccination course. To explore the interaction between disease severity and vaccine type further we plotted the correlation between MELD score and anti-RBD titres (Fig. 4B). At post-V1, this again showed that increasing disease severity was associated with decreased anti-RBD titres when vaccinated with mRNA platform but not with ChAdOx1. This trend persisted after V2 and V3, although it was non-significant. Among patients with cirrhosis, both univariable and multivariable analyses showed that age over 75 years and increasing MELD score were associated with a lower antibody response, whereas mRNA platform and previous COVID-19 were associated with higher titres (Fig. 4C, Table S7).

#### Patients with vascular liver disease

All patients with VLD were vaccinated with an mRNA platform. The VLD cohort had lower post-V2 responses compared to mRNA-vaccinated HCs but equivalent titres post-V3. Patients with VLD had higher post-V2 antibody titres compared to mRNA-vaccinated LTRs (*p* < 0.0001), and patients with cirrhosis (*p =* 0.004) and AIH (*p* < 0.0001) (Tables S1 and S2).

### T-cell responses

#### T-cell responses to WT SARS-CoV-2 across liver disease phenotypes

**Fig. 5:**
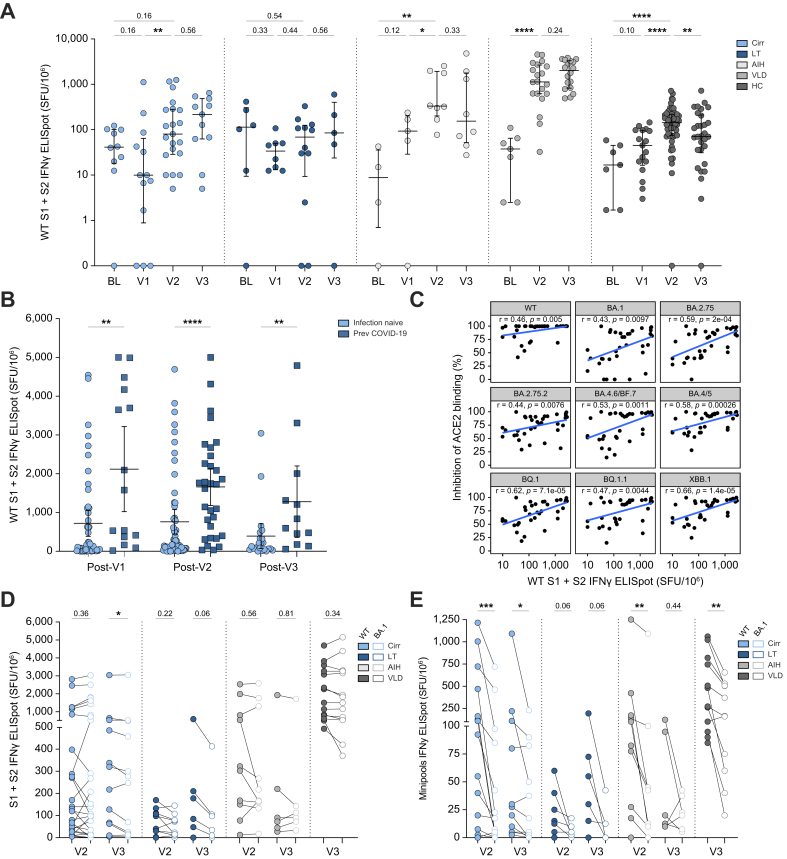
IFNγ T-cell responses to COVID-19 vaccination. (A) Magnitude of IFNγ T-cell response to WT SARS-CoV-2 spike peptides across time in a subgroup of SARS-CoV-2-naïve individuals with cirrhosis (Cirr, n = 24), autoimmune hepatitis (AIH, n = 12), or vascular liver disease (VLD, n = 22), as well as in liver transplant recipients (LTRs, n = 12) and healthy controls (HCs, n = 28). Baseline data are from same individuals as later timepoints. (B) IFNγ T-cell responses in SARS-CoV-2-naïve (n = 68) and previously infected individuals (n = 31) across all disease groups. (C) Spearman correlation between magnitude of IFNγ T-cell responses to WT spike and percent inhibition of ACE2 binding to Omicron subvariant RBD by serum at post-V2 timepoint. (D,E) Magnitude of IFNγ T-cell responses to WT (filled circles) and Omicron BA.1 (open circles) peptides after two or three vaccines covering (D) whole spike and (E) minipools. (A,B) Line represents median and whiskers IQR. (C) Line represents linear regression. (A,B) Mann-Whitney *U* test. (D&E) Wilcoxon matched-paired signed rank test. n.s., non-significant, ∗*p* < 0.05,∗∗*p* < 0.01,∗∗∗*p* < 0.001,∗∗∗∗*p* < 0.0001. AIH, autoimmune hepatitis; BL, baseline; RBD, receptor binding domain; SFU, spot-forming units; WT, wild-type.

T-cell IFNγ responses to WT virus for a subset of infection-naïve participants from each disease group are presented in Fig. 5A and Fig. S7. The majority of patients across the entire cohort had positive T-cell responses (>26 SFU/10^6^ PBMCs) after at least one dose of vaccination. Within the liver disease cohort all patients generated a positive T-cell response after V2 except for 3/10 (30%) LTRs, 4/24 (17%) with cirrhosis, and 1/12 (8%) with AIH. Despite significant heterogeneity, all groups except for LTRs had a significant increase in the magnitude of IFNγ responses after two or three vaccine doses (Fig. 5A). Within the total liver disease cohort, patients with previous COVID-19 had significantly higher IFNγ responses after V1, V2, and V3 (Fig. 5B). There were positive correlations between post-V2 T-cell responses to WT spike, anti-RBD binding antibodies to WT, and functional antibody responses to all variants (ACE2 binding inhibition) (Figs 5C and S4A).

#### T-cell responses to Omicron BA.1 SARS-CoV-2 across liver disease phenotypes

To determine the cross-reactivity of vaccine-induced cellular responses we additionally assessed IFNγ T-cell responses to peptides covering the Omicron (BA.1) spike protein (Fig. 5D). Compared to the WT antigen, T-cell responses to BA.1 spike were well preserved regardless of disease group at post-V2 and post-V3 timepoints. However, when only assessing responses to peptides that differed between WT and BA.1 spike (mutated peptide pools; “minipools”) there were significant reductions in BA.1 peptide-specific reactivity compared to WT in all groups (Fig. 5E), indicating that T-cell responses specifically to mutated epitopes were reduced but overall responses were maintained.

### Breakthrough SARS-CoV-2 infection after vaccination

**Fig. 6:**
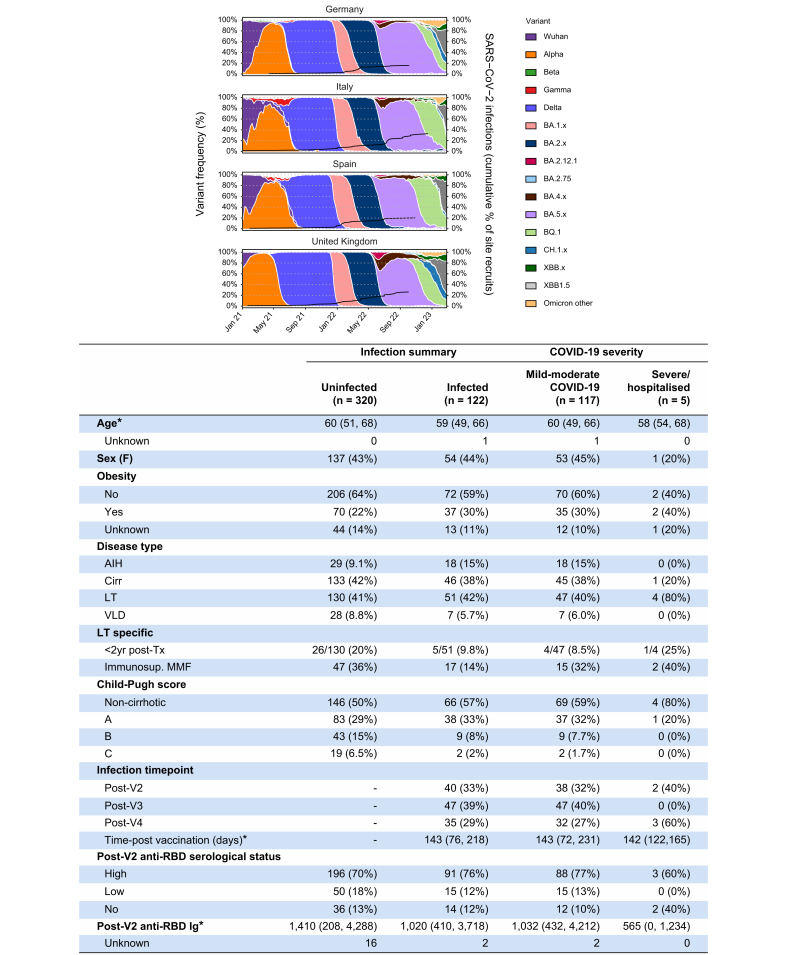
Breakthrough SARS-CoV-2 infection after two COVID-19 vaccines in liver disease cohort (n = 442). (A) Reported SARS-CoV-2 infection after second dose vaccine split across recruitment site countries. Frequency of SARS-CoV-2 and Omicron subvariants per country per week with cumulative proportion of infected individuals out of total individuals recruited at each site (black line). (B) Demographics and immunogenicity of cases with breakdown of severity (bottom panel). ∗Median and IQR. AIH, autoimmune hepatitis; Cirr, cirrhosis; LT, liver transplant; MMF, mycophenolate mofetil; VLD, vascular liver disease. (This figure appears in color on the web.)

SARS-CoV-2 infection status after COVID-19 vaccination was available for 442/722 (57%) of the entire liver disease cohort (Fig. 6A). Of these cases, 122/442 (28%) developed breakthrough infection after at least two vaccines; 40/122 (33%) after V2 and 47/122 (39%) after V3, and 35/122 (29%) after fourth vaccine (Fig. 6B). In those developing breakthrough infections, 117/122 (96%) were mild-moderate and 5/122 (4%) were severe. The majority (101/122, 83%) of breakthrough infections occurred after the emergence of Omicron as the dominant SARS-CoV-2 variant in each recruiting country. A breakthrough infection occurred in 51/181 (28%) LTRs, 46/179 (26%) patients with cirrhosis, 18/47 (38%) with AIH and 8/35 (23%) with VLD. Of those with severe breakthrough COVID-19, 4/5 (80%) were LTRs, two of whom were immunosuppressed with MMF and had absent anti-RBD responses after V2. Although sample size prevented robust statistical comparisons, the median post-V2 anti-RBD titre was numerically lower in those with severe COVID-19 compared to mild-moderate disease (565 U/ml [0-1,234] severe; 1,032 U/ml mild-moderate [432-4,212]) (Fig. S8). Two of five individuals with severe symptomatic breakthrough infection had PBMCs available at the post-V2 timepoint and had similar T-cell responses to WT spike (163 SFU/10^6^ PBMCs and 65 SFU/10^6^ PBMCs) as other LTRs (median 65 SFU/10^6^ PBMCs) (Fig. 5A).

## Discussion

In a large, international, prospective study we assess humoral and cellular immune responses to multiple COVID-19 vaccine platforms across a range of liver disease types and severities using standardised timepoints and laboratory assays. We report on functional antibody and cellular responses to novel viral variants including a number of the most up-to-date Omicron subtypes, and assess COVID-19 infection rates and disease severity.

Longitudinal serum sampling demonstrated a stepwise increase in the magnitude of anti-SARS-CoV-2 RBD antibodies following one, two and three vaccine doses, and following past SARS-CoV-2 infection. In our cohort, use of the mRNA platform was associated with greater antibody titres compared to ChAdOx1 which is in line with other datasets in healthy and immunosuppressed cohorts.^20^^,^^21^ In addition, as observed in healthy populations,^22^ heterologous first and second vaccination was associated with >5-fold increase in post-V2 antibody responses compared to homologous vaccine delivery, suggesting that this approach should also be considered in liver cohorts.

Our data identifies LTRs as a particularly vulnerable cohort, having the lowest post-V2 antibody titres compared to all other disease groups. Although the number of cases of severe breakthrough infection in our cohort was small, it is notable that 4/5 (80%) were LTRs, two of whom received MMF therapy and had absent anti-RBD responses after V2. Vaccine immunogenicity in LTRs was heavily influenced by intensity of immunosuppressive medication with MMF associated with low antibody titres and high rates of non-response and MMF dose negatively associated with anti-RBD titres. This supports evidence from other patient groups that short-term discontinuation or dose reductions of immunosuppressive therapy may help maximise antibody responses to vaccination.^23^^,^^24^ Although other studies have reported suboptimal antibody responses in LTRs,^25^^,^^26^ our cohort is notable for its size and geographic diversity. It also allows for direct comparisons with immunosuppressed patients with AIH who have more robust antibody responses despite being of similar age. This is most likely accounted for by the predominant use of thiopurines and corticosteroids, and the absence of MMF plus CNI dual therapy in patients with AIH. Reassuringly, irrespective of immunosuppressive status, antibody titres and rates of seroconversion were universally improved in all groups following the third vaccination, though some LTRs remained non-responsive to vaccination.

In parallel with increasing serological titres to WT SARS-CoV-2, the cross-reactivity of vaccine-induced antibodies to VoC also improved across the liver disease cohort between post-V2 and post-V3 timepoints. This suggests that increasing the magnitude of serological response through a third vaccination maximises the likelihood that a proportion of antibodies will cross-react with VoC even in individuals who may have reduced capacity to produce high-affinity class-switched or somatically hypermutated antibodies.^27^ Despite improvements in antibody function with repeat vaccination, there was still a significant decrease in IgG binding and ACE-2 binding inhibition to nearly all Omicron subvariants in patients with liver disease after V2 and V3 relative to WT virus. This immune escape may partly account for the clear stepwise increments in breakthrough infection rates observed in our cohort.

Early in the vaccination course, severity of cirrhosis (indicated by CP class and MELD score) was associated with lower antibody responses to mRNA but not ChAdOx1. Although this effect was ultimately overcome by repeated vaccine doses it does point towards differential immunological mechanisms governing antibody response according to vaccine type. mRNA and adenoviral vector platforms are thought to induce antibody production through varying biological pathways which may be differentially impacted by cirrhosis-associated immune dysfunction.^28^^,^^29^ Further work is required to decipher the complex interplay between cirrhosis-associated immune dysfunction and immune responses to COVID-19 vaccination. Nonetheless, it is important to note that mRNA vaccines still induce higher antibody titres than ChAdOx1 at the post-V2 timepoint.

Despite heterogeneity in the magnitude of T-cell responses across HCs (as observed elsewhere^18^) and disease cohorts, the majority of assessed individuals (88%) mounted an IFNγ response to WT SARS-CoV-2 spike antigens after a single vaccine dose which was preserved after V2 and V3. Notably, nearly all infection-naïve participants had low-level T-cell responses detected at baseline, likely as a consequence of cross-reactivity with seasonal human coronaviruses, or possible undetected previous COVID-19 due to waning of post-infection nucleocapsid antibodies.[30], [31], [32] Patients with VLD had particularly robust IFNγ T-cell responses compared to other disease cohorts, which is possibly related to the absence of immunosuppressive medications, a possible unknown biological mechanism, preserved liver function, and use of mRNA-1273 vaccination which has previously been shown in healthy individuals to be associated with higher CD4+ and CD8+ SARS-CoV-2-specific T-cell responses.^21^ Overall, T-cell responses in individuals with liver diseases were also well maintained against the Omicron BA.1 variant, however reduced responses to BA.1 minipools suggest that responses to specific mutated epitopes may be lost.^33^ The cellular immune response to vaccination has emerged as a major determinant of individual risk of developing severe COVID-19, including in immunocompromised individuals.^13^^,^^14^ This may help explain why the majority of breakthrough infections reported in the liver disease cohort were mild-moderate with only 5/122 (4%) total infections reported as severe. However, post-V2 T-cell responses were detectable in the two participants with severe breakthrough COVID-19 and available PBMCs in this cohort, and other factors including potentially reduced virulence of later SARS-CoV-2 variants may additionally impact this.^34^

There are some limitations to our study. Firstly, despite recruiting from four different countries with diverse immunisation regimens, certain vaccine platforms in particular groups are lacking, including mRNA-1273-vaccinated HCs, third dose of ChAdOx1 in HCs, and ChAdOx1-or BNT162b2-vaccinated patients with VLD. Although it is tempting to extrapolate the immunological principles identified across three vaccines, the precise immune changes after multiple subsequent vaccine doses remain to be determined. Another constraint of our dataset is that the HC cohort is comprised of healthcare workers who are significantly younger with fewer comorbidities than the liver disease population. However, we have performed a multivariable analysis of the entire cohort which accounts for age in order to identify cofactors and disease groups associated with vaccine response. The breakthrough COVID-19 data must also be interpreted with caution as asymptomatic infection may not have been identified and it remains impossible to fully account for important confounding variables such as local SARS-CoV-2 prevalence, viral load exposure, further vaccine doses, and individual patient behaviours including shielding measures. Furthermore, we were unable to systematically collect accurate data on the use of antiviral medications and recombinant antibodies due to incomplete documentation in electronic hospital records and geographic variability in access to these agents. As a result, we have opted to remain descriptive with this domain of the study and have not performed statistical analyses. Lastly, due to sampling limitations, IFNγ T-cell assay results were not available in all participants with severe breakthrough infection.

In summary, we demonstrate that the three most widely available vaccine platforms are immunogenic and appear to protect against severe COVID-19 in a diverse group of patients with a variety of underlying liver conditions. Even patients with advanced cirrhosis mount robust immune responses after two and three vaccine doses irrespective of vaccine type. This will provide reassurance to patients with chronic liver disease who were previously deemed at high risk of severe COVID-19 and death during the pre-vaccination era. In addition, our data will be encouraging in the event of future unforeseen viral infections which may also require rapid vaccine development and delivery to patients with liver disease. However, we show that LTRs mount lower antibody and T-cell responses, related to intensity of immunosuppression and the use of MMF, with most cases of severe COVID-19 occurring in this patient group. We recommend that LTRs should be vigilantly monitored for the development of severe COVID-19 if infected, and prioritised for repeated vaccination, prophylactic antiviral agents, and enrolment into trials exploring the role of immunosuppressive dose modification and alternative vaccine strategies.

## Abbreviations

AIH, autoimmune hepatitis; CNI, calcineurin inhibitor; ChAdOx1, ChAdOx1 nCoV-19; CP, Child-Pugh; HC, healthy control; MELD, model for end-stage liver disease; MMF, mycophenolate mofetil; RBD, receptor binding domain; LTRs, liver transplant recipients; VoC, variant of concern; VLD, vascular liver disease; WT, wild-type.

## Financial support

The COVID-Hep vaccine network is supported by a registry grant from the European Association for the Study of the Liver (EASL). The UK OCTAVE study is funded by a grant from UK Research and Innovation (UKRI) administered by the Medical Research Council (reference: MC_PC_20031). PITCH is funded by the UK Department of Health and Social Care by UKRI as part of “Investigation of proven vaccine breakthrough by SARS-CoV-2 variants in established UK healthcare worker cohorts: SIREN consortium & PITCH Plus Pathway (reference: MR/W02067X/1), with contributions from UKRI/NIHR through the UK Coronavirus Immunology Consortium (UK–CIC), the Huo Family Foundation and the NIHR UKRIDHSC COVID-19 Rapid Response Rolling Call (reference: COV19-RECPLAS). SMM is supported by the Medical Research Council. T.M. is supported via a Wellcome Trust Clinical Research Training Fellowship (reference: 102176/B/13/Z). E.B. is supported by the Oxford NIHR Biomedical Research Centre and is an NIHR Senior Investigator. P.J.T receives institutional salary support from the National Institute for Health Research (NIHR) Birmingham Biomedical Research Centre (BRC). BHM is the recipient of an NIHR Academic Clinical Lectureship (CL-2019-21-002). A.W.L, J.S.z.W., M.L, receive financial support from the German Center for Infection Research (DZIF). Part of the work of this study (P.G.) has been funded by a grant of the Instituto de Salud Carlos III-ISCIII, grant number: PI020/00579. The Division of Digestive Diseases at Imperial College London receives financial and infrastructure support from the NIHR Imperial Biomedical Research Centre (BRC) based at Imperial College Healthcare NHS Trust and Imperial College London. The views expressed in this article are those of the authors and not necessarily those of the NHS, the NIHR, or the Department of Health.

## Conflicts of interest

E.B. and P.K. have received consultancy fees from AstraZeneca. E.B has received consultancy fees from Vaccitech. P.L. is on the advisory board for BMS, Roche, Gilead Sciences, GSK, AbbVie, MSD, Arrowhead, ALNYLAM, Janssen, SBRING Bank, MYR, Eiger, Antios, ALIGOS, VI. M.I. is on the advisory board or has been a speaker for Bureau for Bayer, Gilead Sciences, BMS, Janssen, Ipsen, MSD, BTG-Boston Scientific, AbbVie, Guerbet, EISAI, Roche, AstraZeneca. P.J.T has received grant support from the Wellcome Trust, the Medical Research Foundation, LifeArc, Innovate UK, GSK, Guts UK, PSC Support, Intercept/Advanz Pharma, Dr. Falk Pharma, Gilead sciences, and Bristol Myers Squibb, speaker fees from Intercept and Dr Falk, and advisory board/consultancy fees from Albireo, Cymabay, Pliant Pharma, IPSEN, Intercept, Dr. Falk and GSK. MCL has received advisory fees from Intercept/AdvanzPharma, IPSEN, GSK and lecture fees from Intercept/AdvanzPharma. All other authors declare no conflicts of interest.

Please refer to the accompanying ICMJE disclosure forms for further details.

## Authors’ contributions

TM and EB are Chief Investigators of the EASL supported COVID-Hep vaccine network. SJD and PK are the Chief Investigators of the PITCH Study. EB, SJD, are members of the OCTAVE Trial Management Group. TM and EB wrote the grant proposal for EASL registry funding. TM, EB, FPR, JSZW, MI, ML, EP wrote the EASL network study protocol. SMM, GM, SI, NP performed T-cell laboratory assays. ML and JSZW performed antibody titre assays. MC, TT, and SH performed variant of concern antibody assays. TM, EB, MW, GS, PD, ZL, SD, GM, ML, PT, KB, BM, PM, EP, MI, MS, JSZW, PG, VHG, JGP, VP were all involved in sample and/or clinical data collection. SMM, JC, and TM performed the statistical analysis. SMM and TM wrote the initial manuscript. All named authors contributed to the interpretation of the analyses and the writing of the paper. The corresponding authors had full access to all the data in the study and had final responsibility for the decision to submit for publication.

## Data availability statement

Data may be made available upon reasonable request to corresponding author.
