# Supplementary material for: Immune responses and clinical outcomes after COVID-19 vaccination in patients with liver disease and liver transplant recipients
Source: J Hepatol. 2024 Jan;80(1):109–23. doi: 10.1016/j.jhep.2023.10.009 (PMC10914634; doi:10.1016/j.jhep.2023.10.009)
Supplement: Multimedia component 1 [file mmc1.pdf]

Immune responses and clinical outcomes after COVID-19 vaccination in patients with liver disease and in liver transplant recipients

Sam M. Murray, Elisa Pose, Melanie Wittner, Maria-Carlota Londoño, Golda Schaub, Jonathan Cook, Stavros Dimitriadis, Georgina Meacham, Sophie Irwin, Zixiang Lim, Paul Duengelhoef, Martina Sterneck, Ansgar W. Lohse, Valeria Perez, Palak Trivedi, Khush Bhandal, Ben Mullish, Pinelopi Manousou, Nicholas M. Provine, Emma Avitabile, Miles Carroll, Tom Tipton, Saoirse Healy, Patrizia Burra, Paul Klenerman, Susanna Dunachie, Barbara Kronsteiner, Agnieszka Katarzyna Maciola, Giulia Pasqual, Virginia Hernandez-Gea, Juan Carlos Garcia-Pagan, Pietro Lampertico, Massimo Iavarone, Pere Gines, Marc Lütgehetmann, Julian Schulze zur Wiesch, Francesco Paolo Russo, Eleanor Barnes, Thomas Marjot on behalf of the OCTAVE Collaborative Group, PITCH study, and the EASL supported COVID-Hep vaccine network

Table of contents

OCTAVE Collaborative group 4
EASL supported COVID-Hep vaccine network 6
PITCH Consortium 7
Supplementary methods 8
Fig. S1 10
Fig. S2 11
Fig. S3 12
Fig. S4 13
Fig. S5 14
Fig. S6 15

Fig. S7 16
Fig. S8 17
Table S1 18
Table S2 19
Table S3 20
Table S4 21
Table S5 22
Table S6 23
Table S7 24
Supplementary reference 25

OCTAVE Collaborative group

Gary Middleton<br>Charlotte Gaskell<br>Daniel Rea<br>Sarah Pirrie<br>Sarah J Bowden<br>Ann Pope<br>Ana Hughes<br>Molly Harrison<br>Amanda Kirkham<br>Lucinda Middleton<br>Faye Lowe<br>Sophia Magwaro	Cancer Centre, University Hospitals Birmingham, NHS Foundation Trust, Birmingham B15 2WB, UK
Pamela Kearns	National Institute for Health Research Birmingham Biomedical Research Centre, Institute of Cancer and Genomic Sciences, University of Birmingham, Birmingham B15 2TT UK.<br>Cancer Research UK Clinical Trials Unit (CRCTU), University of Birmingham, Edgbaston, Birmingham. B15 2TT, UK.
Sean H Lim	Centre for Cancer Immunology, University of Southampton, Southampton, SO16 6YD UK.
Michelle Willicombe<br>Maria Prendecki<br>Candice Clarke<br>Paige Mortimer<br>Stacey McIntyre<br>David Thomas	Centre for Inflammatory Disease, Department of Immunology and Inflammation, Imperial College London, Hammersmith Campus, Du Cane Road, London W12 0NN UK.
Alex Richter<br>Sally Al-Taei	Clinical Immunology Service, University of Birmingham, Edgbaston, Birmingham. B15 2TT, UK.
Carl S Goodyear<br>Stefan Siebert<br>Neil Basu<br>Ashley Gilmour<br>Iain B McInnes	College of Medical, Veterinary & Life Sciences; University of Glasgow, Glasgow; G12 8QQ, UK.
Andrew Tong<br>Kieran Woolcock<br>Faisal Basheer<br>Charles Crawley<br>Ram Malladi<br>Andrew King<br>Sophie Lockey<br>Ben Uttenthal	Department of Haematology, Cambridge University Hospitals NHS Foundation Trust, Cambridge, CB2 OQQ, UK
John A Snowden<br>Rachael Selby	Department of Haematology, Sheffield Teaching Hospitals NHS Foundation Trust, Royal Hallamshire Hospital, Sheffield. S10 2JF, UK.
Kim Orchard	Department of Haematology, University Hospital Southampton NHS Foundation Trust, Southampton, S016 6YD UK.
Thushan I de Silva<br>Naomi Meardon<br>Sam Hansford<br>Gurjinder Sandhar	Department of Infection, Immunity and Cardiovascular Disease, The Medical School, The University of Sheffield, Sheffield. S10 2RX, UK.
Peter Kelleher	Department of Infectious Diseases, Imperial College London, School of Medicine Chelsea and Westminster Hospital, London SW10 9NH UK
Murali Kesavan	Department of Oncology, Cancer and Haematology Centre, Churchill Hospital, Old Road, Headington, Oxford, OX3 7LE, UK
Celia Moore<br>Pinelopi Manousou<br>Gareth Hahn<br>Benjamin Mullish	Division of Digestive Diseases, Department of Metabolism, Digestion and Reproduction, Faculty of Medicine, Imperial College London, London, W2 1NY, UK
Maria Atta	Haematology Department, Hammermith Hospital, London, W12 0HS UK
Sarah Gleeson<br>Liz Lightstone<br>Paul Martin<br>Stephen McAdoo<br>Tina Thomson	Imperial College Healthcare NHS Trust, Hammersmith Hospital, London, W12 OHS, UK
Mickey BC Koh	Infection and Immunity Clinical Academic Group, St George's, University of London; Department of Haematology, St George's University Hospital NHS Foundation Trust, London SW17 0QT
Daniele Avenoso<br>Robin Sanderson	King's College Hospital NHS Foundation Trust, London, SE5 9RS, UK
Claire Taylor	Leeds Institute of Medical Research, University of Leeds, Leeds, LS2 9NL
Khushpreet Bhandal<br>Diana Hall	Liver Research Delivery Team, University Hospitals Birmingham NHS Foundation Trust, Birmingham, B15 2GW, UK
Andrew Filer<br>Palak Trivedi<br>Gordon Cook	National Institute for Health Research (NIHR) Birmingham Biomedical Research Centre and NIHR Clinical Research Facility, Institute of Inflammation and Ageing, University of Birmingham and University Hospitals Birmingham NHS Foundation Trust, Birmingham, B15 2TT, UK

Erin Hurst<br>Amy Publicover<br>Katy Scouse	Northern Centre for Cancer Care, Freeman Hospital, Newcastle upon Tyne, NE7 7DN, UK
Paul Klenerman<br>Susanna J Dunachie,<br>Eleanor Barnes	Nuffield Department of Medicine, University of Oxford, Oxford, OX1 2JD, UK.<br>National Institute for Health Research, Oxford Biomedical Research Centre, Oxford University Hospitals NHS Trust, Oxford, UK.
Sam M Murray<br>Zixiang Lim<br>Jack Satsangi<br>Sophie Irwin<br>Georgina Meacham<br>Thomas Marjot<br>Stavros Dimitriadis<br>Jem Chalk<br>Daniel Hanke<br>Josef Hanke<br>Saoirse Healy<br>Stephen Laidlaw<br>Stephanie Longet<br>Nicholas Provine<br>Sarah Thomas<br>Victoria Walker<br>Zay Win	Nuffield Department of Medicine, University of Oxford, Oxford, OX1 2JD, UK.
Richard Beesley<br>Vicky Churchill<br>Holly Loughton<br>Elspeth Insch<br>Eilean MacDonald	Patient and Public Representatives on the Trial Management Group
Doreen Trown	Sheffield Teaching Hospitals NHS Foundation Trust, Royal Hallamshire Hospital, Sheffield S10 2JF, UK
Patricia Faria<br>Julie Chackathayil	St George’s hospital and Medical School, St George’s University Hospitals NHS Foundation Trust, London, SW17 0QT, UK
Clare Hutchison<br>Deborah Richardson	University Hospital Southampton NHS Foundation Trust, Southampton General Hospital, Southampton, SO16 6YD, UK
Maxine Arnott<br>Louise Bennett<br>James Brock<br>Victora Keillor<br>Andrew Melville<br>Lisa Melville<br>Samantha Miller<br>Aurelie Najm<br>Caron Paterson<br>Lewis Rodgers<br>Matthew Rutherford<br>Suzann Rundell<br>Emily Smith<br>Lynn Stewart<br>Flavia Sunzini	University of Glasgow, Glasgow, G12 8QQ, UK
Miles Carroll	Wellcome Centre for Human Genetics, University of Oxford, Oxford, UK.

EASL supported COVID-Hep vaccine network

Patrizia Burra	University of Padova, Department of Surgery, Oncology and Gastroenterology DISCOG, Italy
Francesco Paolo Russo	
Paola Zanaga	
Thomas Marjot	Oxford Centre for Diabetes, Endocrinology and Metabolism (OCDEM), NIHR Oxford Biomedical Research Centre, Churchill Hospital, University of Oxford, Oxford, UK
Sam M. Murray	Nuffield Department of Medicine, University of Oxford, Oxford, OX1 2JD, UK.
Eleanor Barnes	
Victoria Walker	
Anthony Brown	
Georgina Meacham	
Sophie Irwin	
Marc Lütgehetmann	Institute of Medical Microbiology, Virology and Hygiene, University Medical Center Hamburg-Eppendorf, Hamburg, Germany
Melanie Wittner	Department of Internal Medicine, University Medical Center Hamburg-Eppendorf, Hamburg, Germany
Golda Schaub	
Paul Duengelhoef	
Martina Sterneck	
Ansgar W. Lohse	
Julian Schulze zur Wiesch	
Pietro Lampertico	Division of Gastroenterology and Hepatology, Foundation IRCCS Ca' Granda Ospedale Maggiore Policlinico, Milan, Italy
Massimo Iavarone	
Jonathan Cook	Centre for Statistics in Medicine, University of Oxford, Oxford, UK
Maria-Carlota Londoño	Liver Unit, Hospital Clínic, Institut de Investigacions Biomèdiques August Pi i Sunyer (IDIBAPS), University of Barcelona, Barcelona. CIBEREHD (Centro de Investigación Biomédica en Red Enfermedades Hepáticas y Digestivas). Health Care Provider of the European Reference Network on Rare Liver Disorders (ERN-Liver), Spain
Elisa Pose	
Valeria Perez	
Virginia Hernandez-Gea	
Juan Carlos Garcia-Pagan	
Pere Gines	
Nicola van Berckel	

PITCH Consortium

Thushan I. de Silva	Department of Infection, Immunity and Cardiovascular Disease, University of Sheffield, Sheffield, UK
Sarah L. Rowland-Jones	
Sian Faustini	Institute for Immunology and Immunotherapy, College of Medical and Dental Science, University of Birmingham, Birmingham, UK
Alex Richter	
Susan L Dobson	Institute of Infection, Veterinary and Ecological Sciences, University of Liverpool, Liverpool, UK
Shona C Moore	
Lance Turtle	
Daniel G. Wootton	
James E.D. Thaventhiran	MRC Toxicology Unit
Donal Skelly	Nuffield Department of Clinical Neurosciences, University of Oxford, Oxford, UK
Priyanka Abraham	Nuffield Department of Medicine, University of Oxford, Oxford, UK
Sandra Adele	
Mohammad Ali	
Eleanor Barnes	
Anthony Brown	
Miles Carroll	
Christopher P. Conlon	
Alexandra S Deeks	
Susanna Dunachie	
John Frater	
Lisa Frending	
Siobhan Gardiner	
Anni Jamsen	
Paul Klenerman	
Barbara Kronsteiner	
Stephanie Longet	
Tom Malone	
Alexander J. Mentzer	
Eloise Phillips	
Patpong Rongkard	
Lizzie Stafford	
Sagida Bibi	Oxford Vaccine Group, Department of Paediatrics, University of Oxford, Oxford, UK
Christina Dold	
Teresa Lambe	
Katie Jeffery	Radcliffe Department of Medicine, University of Oxford, Oxford, UK
Christopher JA Duncan	Translational and Clinical Research Institute, Newcastle University, Newcastle-upon-Tyne
Rebecca P. Payne	
Simon Travis	Translational Gastroenterology Unit, University of Oxford, Oxford, UK
Sarah Foulkes	UK Health Security Agency, UK
Victoria Hall	
Susan Hopkins	
Jasmin Islam	
Ashley Otter	

Supplementary methods

Ethical and regulatory approvals

All centres involved in the EASL supported COVID-Hep vaccine registry recruited participants through local ethics approvals as follows: University Medical Center Hamburg-Eppendorf (approved by local ethics committee, Hamburg, Germany) ref No. PV7103 and PV7298; Foundation IRCCS Ca' Granda Ospedale Maggiore Policlinico: part of PolImmuneCOVID study (No. 286_2021) approved by INMI "Lazzaro Spallanzani" Ethics Committee (Roma, Italy); University of Padova: URC code COVID16; University of Barcelona: Reg. No. HCB/2021/0632 approved by Comité de ética e investigación médica (CEIM). The UK OCTAVE study was approved by the UK Medicines and Healthcare Products Regulatory Agency and London and Chelsea Research Ethics Committee (REC reference: 21/HRA/0489). The PITCH study is a sub-study of the SIREN study, which was approved by the Berkshire Research Ethics Committee, Health Research 250 Authority (REC reference: 20/SC/0230). All PBMCs collected at participating sites that were centralised to the University of Oxford were transferred and stored in accordance with the UK Human Tissue Act.

Anti-SARS-CoV-2 VoC IgG binding and ACE2 inhibition

In order to assess antibody responses to VoC, IgG titres to the spike protein of wild-type SARS-CoV-2 and nine of the most prevalent Omicron subvariants (as of February 2023: B.1.1.529/BA.1/BA.1.15, BA.2.75, BA.2.75.2, BA.4.6, BA.5, BF.7, BQ.1, BQ.1.1, and XBB.1) were assessed using a multiplexed MSD® immunoassay (K15668U). In brief, antigens were spotted at 200−400 µg/mL in 96-well plates which were blocked with MSD® Blocker A for 30 minutes. Following washing plasma/serum samples were diluted 1:10,000 and 1:30,000 in diluent buffer and incubated for 2 hours. Samples were then washed and detected using a MESO® SECTOR S 600 Reader. Concentrations were expressed in Units/ml (U/mL).

To assess functional antibody responses, a V-PLEX SARS-CoV-2 Panel 33 (ACE2) Kit (K15679U) was used to measure the ability of serum/plasma samples to inhibit angiotensin-converting enzyme 2 (ACE2) binding to the RBD of wild-type and the same Omicron subvariants listed above. Assays were performed as per manufacturer's instructions with 1:10 and 1:100 dilutions of serum/plasma. Percentage ACE2 inhibition was determined by comparison of chemiluminescence of sample spots compared to negative controls (blanks) on each plate.

IFNγ T-cell ELISpot assay

200,000 thawed PBMCs were rested for 3 hours and added to Multiscreen-IP filter plates (Millipore) coated with capture antibody (clone 1-D1K). Overlapping peptide pools (18-mers with 10 amino acid overlap, Mimotopes) representing wild-type S1 and S2 regions, membrane, and N proteins were added at a final concentration of 2ug/ml for 16–18hrs at 37°C. Selected samples also included pools covering

the entire Omicron (B.1.1.529, BA.1) S1 and S2 regions, and pools including only peptides which contained mutations in BA.1 spike, or the analogous peptides from wild-type (minipools). CEF and concanavalin A were used as positive controls, DMSO in Rab10 was used as a negative control. Plates were developed and then read using a CTL immunocapture (Cellular Technology Limited) plate reader, using Smartcount® settings. Mean spots from DMSO negatives are removed from stimulation to give antigen-specific responses. A positive IFNγ response was defined as mean DMSO + 2 standard deviations.

Breakthrough SARS-CoV-2 infection after COVID-19 vaccination

Rates of breakthrough infection were plotted over time for each of the 4 recruiting countries alongside the corresponding proportions of circulating viral variants. Country-specific proportions of SARS-CoV-2 variants were calculated based on data shared via GISAID [1] EpiCoV database, downloaded 23 March 2023. The date on which Omicron became the dominant variant (defined as representing >90% of circulating variants) was 1st January 2022 in the UK, 16th January 2022 in Italy and Spain, and 23rd January 2022 in Germany.

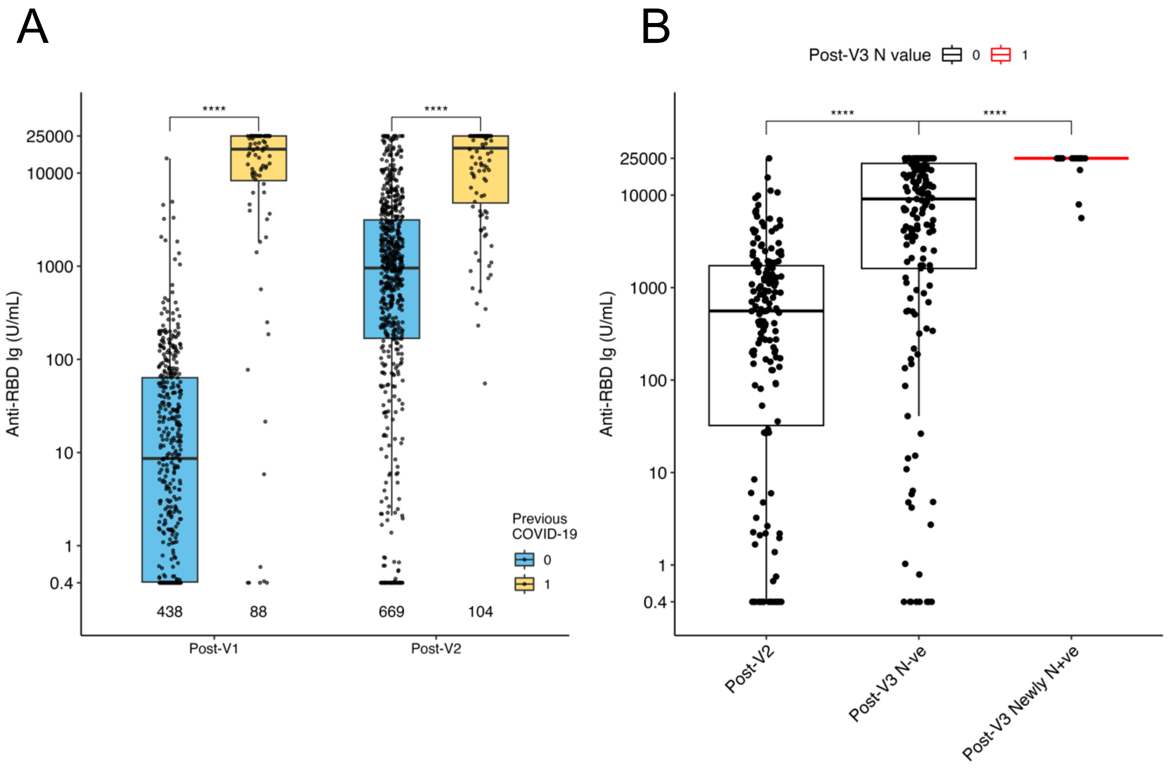

Fig. S1. A) Magnitude of anti SARS-CoV-2 RBD Ig in infection naïve and previously SARS-CoV-2 infected individuals at post-V1 and post-V2 timepoints. B) Magnitude of anti SARS-CoV-2 RBD Ig in Naïve individuals at post-V2 and post-V3 timepoints and in individuals who became nucleocapsid positive between second and third vaccines (Post-V3 Newly N+ve). Boxes represent median and IQR, whiskers represent +/- 1.5x IQR. Mann Whitney U test used, adjusted P value presented.

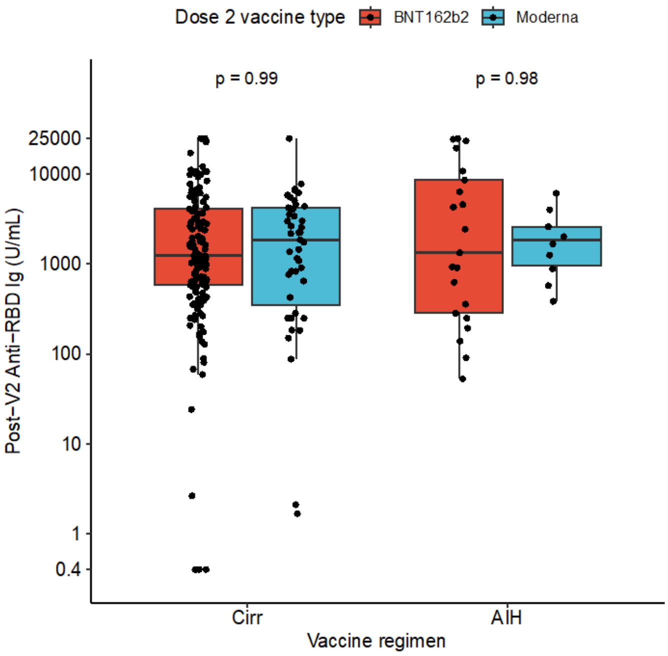

Fig. S2. Magnitude of anti-SARS-CoV-2 RBD Ig in BNT162b2 and mRNA=1273 (Moderna) vaccinated SARS-CoV-2 infection naive individuals from the cirrhosis and autoimmune hepatitis (AIH) disease groups at the post-V2 timepoint. Boxes represent median and IQR, whiskers represent +/- 1.5x IQR. Mann-Whitney U test used, adjusted P value presented.

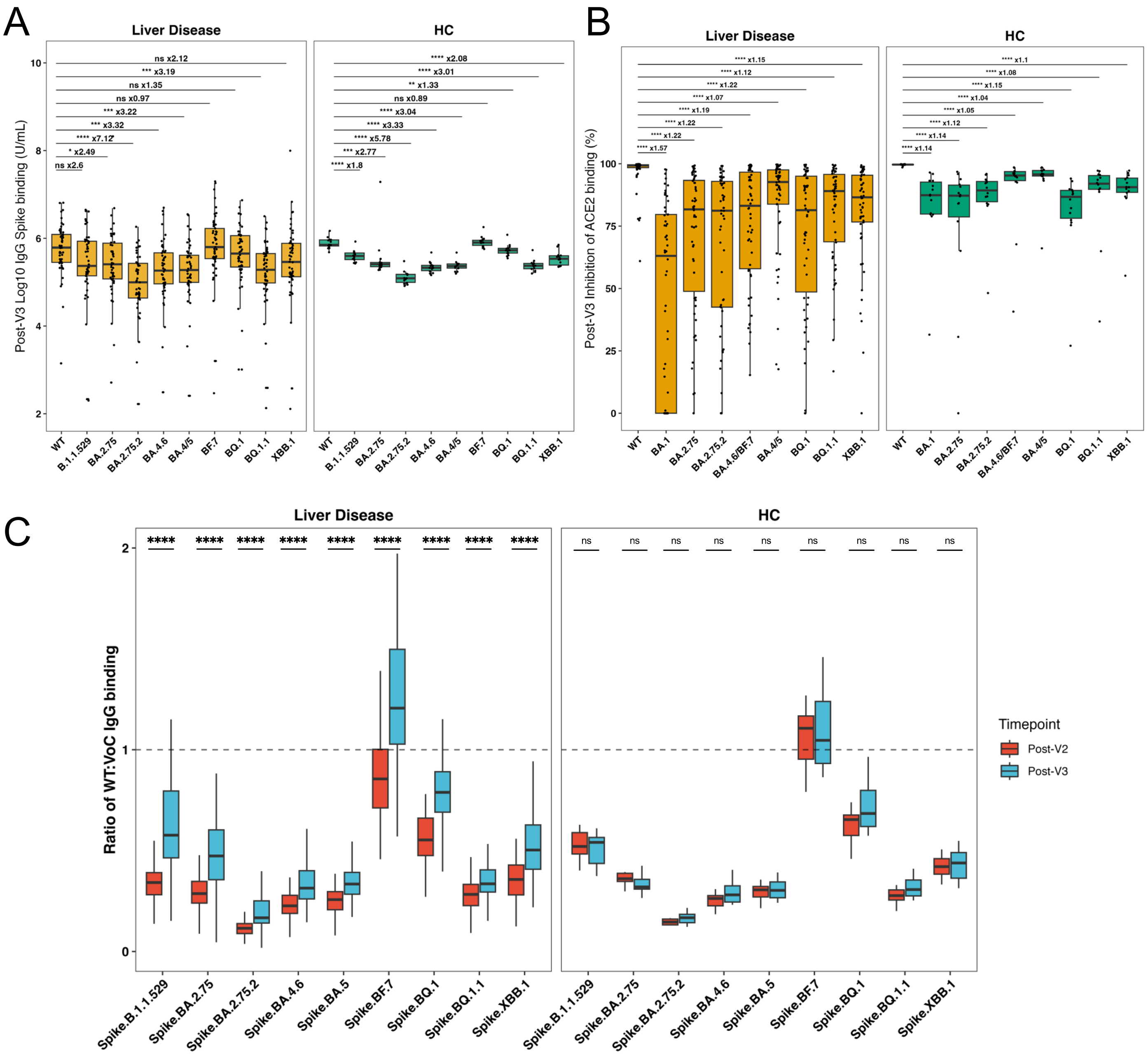

Fig. S3. Post-v3 IgG (A) and ACE2 inhibition (B) to SARS-CoV-2 VoC, separated by liver disease versus healthy controls. C) Ratio of IgG binding to WT and each respective VoC at post-v2 and post-v3 timepoints in liver disease and HC. Two-sided Mann-Whitney U test adjusted with Holm-Bonferroni. Fold-change of median depicted. Boxes represent median and IQR, whiskers represent +/- 1.5x IQR. HC = healthy controls; ACE2 = angiotensin-converting enzyme 2, WT = wild-type. * = P<0.05, ** = P<0.01, *** = P<0.001, **** = P < 0.0001.

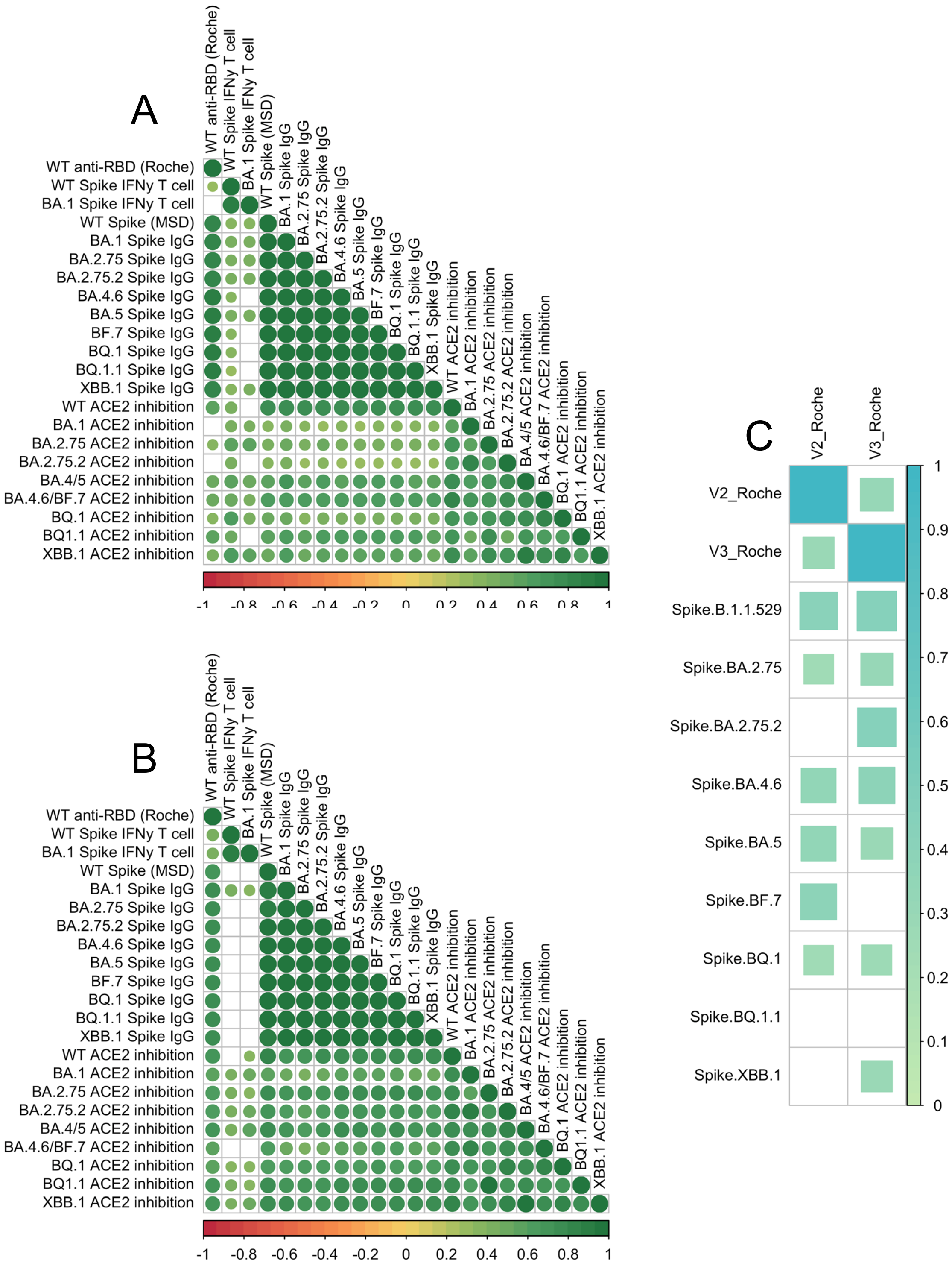

Fig. S4. Correlation of immune assays at A) post-v2 and B) post-v3 timepoints. C) correlation of WT anti-RBD Ig with ratios of WT:VoC binding at post-V2 and post-V3 timepoint. Only significant correlations (P<0.05) are shown. Spearman's correlation. Size and shade of spots/squares represent r value. V2_Roche = post-V2 anti-RBD Ig; V3_Roche = post-V3 anti-RBD Ig

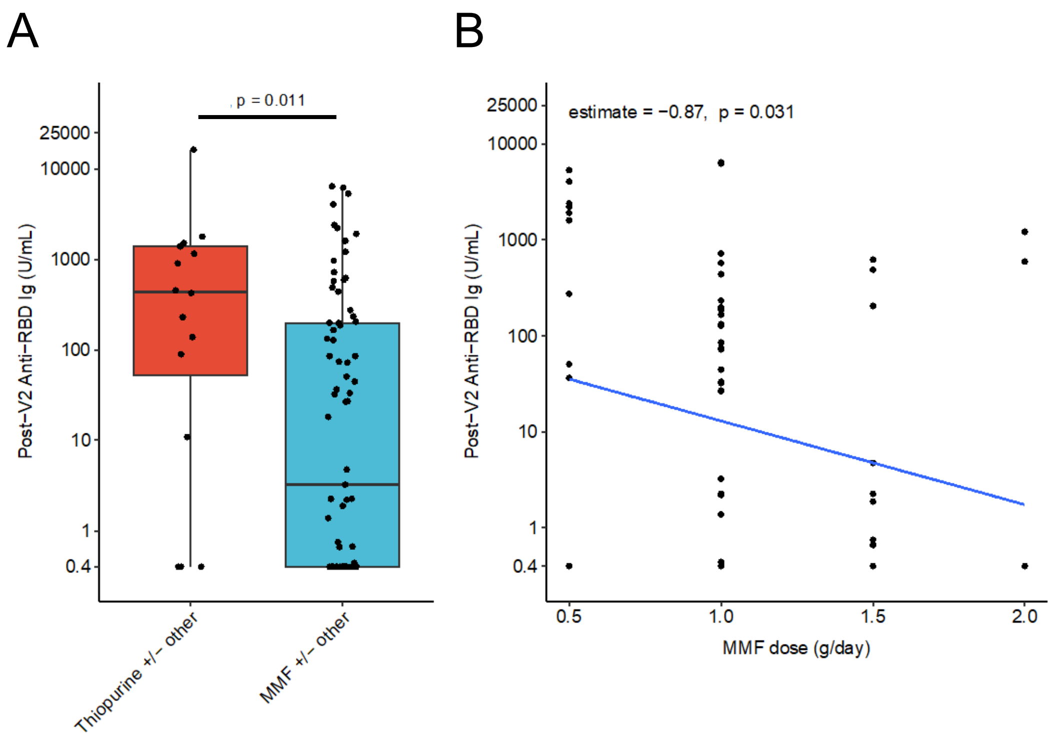

Fig. S5. A.) Anti-RBD Ig responses at the post-V2 timepoint in SARS-CoV-2 infection naive LT recipients who received either thiopurine (Azathioprine or 6-mercaptopurine) or Mycophenolate mofetil (MMF) as an immunosuppressive therapeutic. Participants may have received other immunosuppressive therapeutics in addition. B) MMF dose breakdown (gram/day) in SARS-CoV-2 infection naïve LT recipients at post-V2 timepoint. Linear model of log10 transformed Anti-RBD Ig compared with daily MMF dose. Line represents linear fit, shading represents 95% confidence interval. Boxes represent median and IQR, whiskers represent +/- 1.5x IQR. A) Statistical comparison with Mann-Whitney U test.

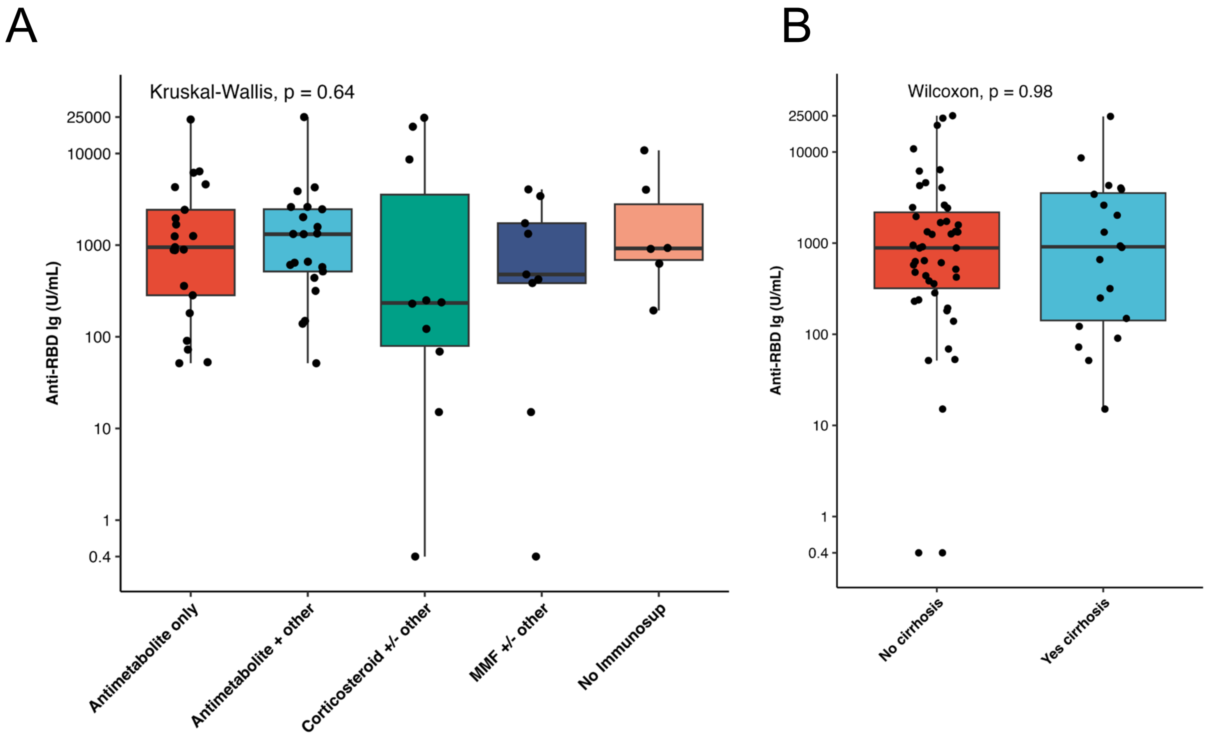

Fig. S6. SARS-CoV-2 infection naïve patients with autoimmune hepatitis at post-v2 timepoint, comparing A) immunosuppressive therapies and B) presence of cirrhosis. Boxes represent median and IQR, whiskers represent +/- 1.5x IQR. Kruskal Wallis (A) or Two-sided Mann-Whitney U test (B). Antimetabolites include 6-mercaptopurine and azathioprine. MMF = Mycophenolate mofetil.

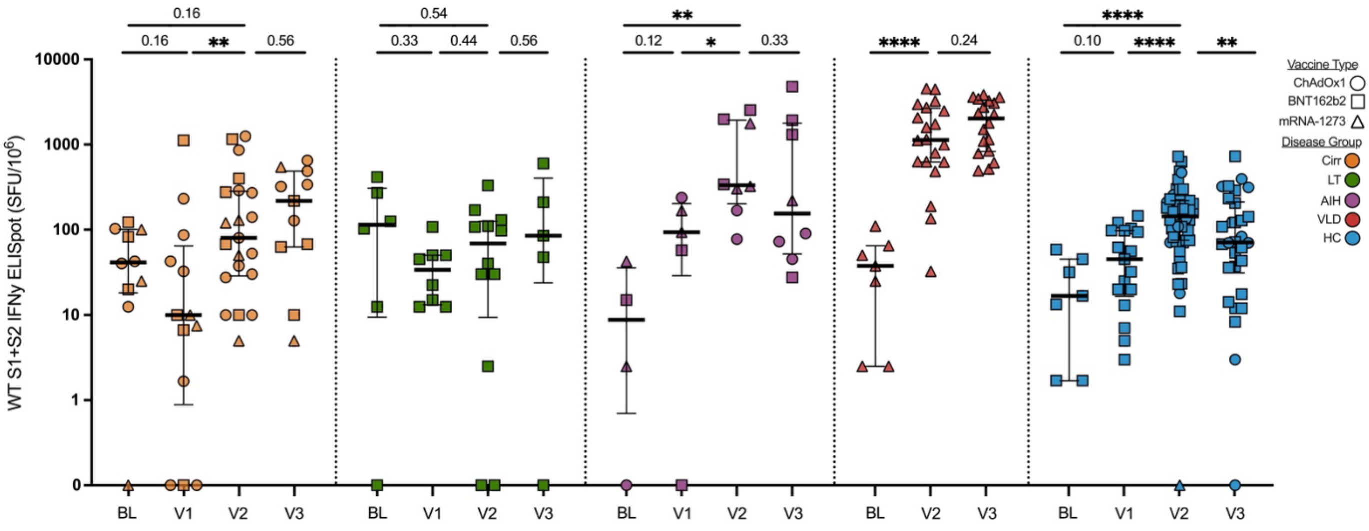

Fig. S7. As in Figure 5A, magnitude of IFN-γ T cell response to wild-type SARS-CoV-2 spike peptides across time in a subgroup of SARS-CoV-2 naïve people with cirrhosis (Cirr, n = 24), liver transplant recipients (LT, n = 12), autoimmune hepatitis (AIH, n=12), vascular liver disease (VLD, n = 22) and healthy controls (HC, n = 28). Baseline data are from same individuals later timepoints. Vaccine type indicated by point of shape. Mann-Whitney U test.

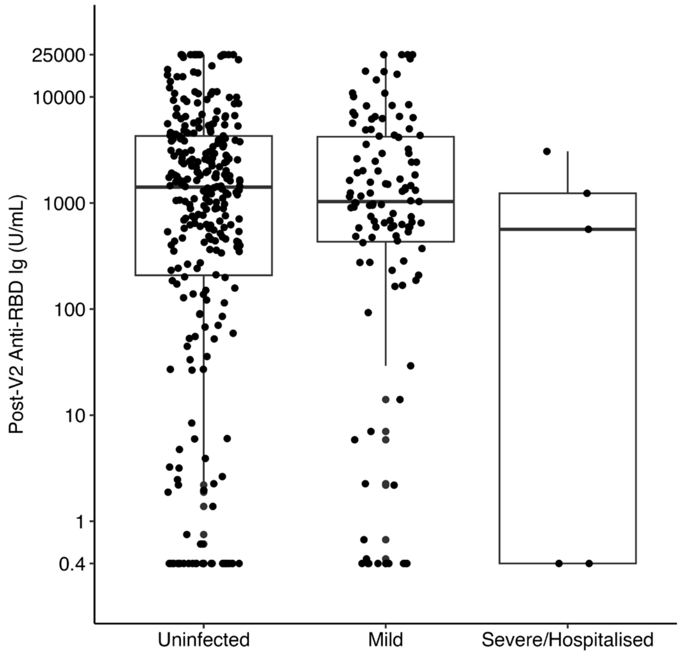

Fig. S8. Anti-RBD Ig at post-v2 timepoint in individuals who did not get breakthrough SARS-CoV-2 infection, had mild-moderate breakthrough SARS-CoV-2 infection or had severe SARS-CoV-2 breakthrough infection after vaccination.

		Baseline		Post-V1			Post-V2			Pre-V3			Post-V3			
Vaccine Type	Disease group	N	Median (IQR)	N	Median (IQR)	P val[1]	N	Median (IQR)	Pval[2]	N	Median (IQR)	P val[3]	N	Median (IQR)	P val[4]	P val[5]
ChAdOx1	Cirr	15	0.4 (0.4-0.4)	34	19.6 (3.3-37.1)	1	85	1106 (395-1838)	7.2×10^{-11}	4	637 (281-6956)	1	6	15368 (11784-22791)	1	0.3
	AIH	6	0.4 (0.4-0.4)	20	15.1 (1.6-41.9)	1	34	498 (129-1323)	4.3×10^{-4}	2	312 (237-387)	1	3	15401 (11133-20201)	1	1
	LT	6	0.4 (0.4-0.4)	27	0.4 (0.4-19.0)	1	52	63 (0.4-608)	0.1	2	151 (133-170)	1	2	3133 (1572-4694)	1	1
	HC	-	-	10	187.5 (87.0-392)	N/A	20	1198 (855-1546)	0.02	-	-	N/A	-	-	N/A	N/A
mRNA	Cirr	156	0.4 (0.4-0.4)	153	13.3 (2.5-55.6)	9.2×10^{-11}	204	1413.5 (578-4140)	6.6×10^{-23}	52	476 (214-958)	0.35	102	18015 (5927-25000)	1.5×10^{-8}	2.2×10^{-6}
	AIH	19	0.4 (0.4-0.4)	19	16.6 (1.8-71.6)	0.87	33	1341 (577-4605)	0.002	11	815 (316-2035)	1	10	18280.5 (13784-25000)	0.73	1
	LT	117	0.4 (0.4-0.4)	119	0.4 (0.4-1.6)	0.09	179	114 (0.4-1113)	2.9×10^{-13}	105	92 (7-390)	1	95	1861 (34-13534)	0.003	9.4×10^{-5}
	VLD	30	0.4 (0.4-0.4)	29	147 (116-203)	0.10	27	4880 (3203-7663)	4.7×10^{-4}	25	1943 (1441-2907)	1	24	25000 (25000-25000)	0.002	0.1
	HC	-	-	27	98.7 (57.2-148)	N/A	35	15634 (10829-21445)	3.2×10^{-11}	22	2171 (1584-3416)	0.007	23	25000 (18359-25000)	2.1×10^{-5}	1

Table S1: In SARS-CoV-2 infection naïve individuals, Roche anti-RBD antibody response to one, two and three doses of COVID-19 vaccine, separated by vaccine type and disease group. [1] comparison of Baseline and Pre-V2, [2] comparison of Pre-V2 and Post-V2, [3] comparison of Post-V2 and Pre-V3, [4] comparison of Pre-V3 and Post-V3, [4] comparison of Post-V2 and Post-V3. Kruskal Wallis with Dunn's post-hoc test, adjusted for multiple comparisons using Bonferroni correction. Cir = Cirrhosis, AIH = Autoimmune hepatitis, LT = Liver transplant, Az = AstraZeneca vaccine.

	Univariable		Multivariable	
**Variable**	**Estimate (95% CI)**	**P value**	**Estimate (95% CI)**	**P value**
Age 45-64	-0.6 (-0.8 - -0.3)	4.81E-06	-0.3 (-0.6 - -0.1)	0.0034
Age 65-74	-0.9 (-1.2 - -0.7)	5.22E-11	-0.5 (-0.8 - -0.3)	8.91E-05
Age 75+	-1 (-1.4 - -0.7)	1.38E-07	-0.6 (-0.9 - -0.2)	0.001
Male sex	-0.3 (-0.5 - -0.1)	0.0030	-0.1 (-0.3 - 0)	0.12
Obesity	0.2 (-0.1 - 0.4)	0.17	-	NA
Hypertension yes	-0.3 (-0.5 - -0.1)	0.013	0 (-0.2 - 0.1)	0.75
Current smoker	0.2 (-0.1 - 0.5)	0.21	-0.1 (-0.4 - 0.1)	0.33
Previous smoker	0.2 (-0.1 - 0.4)	0.16	0.1 (-0.1 - 0.3)	0.29
AIH	-1 (-1.3 - -0.6)	6.81E-08	-0.3 (-0.7 - 0)	0.084
Cirr	-0.7 (-1 - -0.5)	5.01E-08	-0.1 (-0.4 - 0.2)	0.50
LT	-2.1 (-2.4 - -1.8)	1.11E-46	-1.4 (-1.7 - -1.1)	6.18E-18
VLD	-0.2 (-0.7 - 0.2)	0.33	0.2 (-0.3 - 0.6)	0.50
mRNA vaccine	0.3 (0.1 - 0.5)	0.011	0.4 (0.2 - 0.6)	3.39E-06
Heterologous vaccine	1 (0.3 - 1.7)	0.0074	1.3 (0.8 - 1.9)	6.18E-06
Previous COVID-19	1.4 (1.1 - 1.6)	3.43E-23	1 (0.8 - 1.2)	1.47E-16

Table S2: Linear regression model of post-v2 log10 transformed anti-RBD Ig across entire cohort. Age variable is compared to 18-44year old age group. * indicates significant values (P<0.05)

	Comparison groups		Timepoints	
	Group 1	Group 2	Post-V2 (p val)	Post-V3 (p val)
AstraZeneca	Cirr	AIH	0.06	-
	Cirr	LT	2.00E-08*	-
	Cirr	HC	0.57	-
	AIH	LT	0.01*	-
	AIH	HC	0.06	-
	LT	HC	0.000017*	-
mRNA	Cirr	AIH	0.69	0.50999
	Cirr	LT	2.00E-15*	1.98E-08*
	Cirr	VLD	0.0039*	0.00031*
	Cirr	HC	1.30E-10*	0.06007
	AIH	LT	3.40E-06*	0.00219*
	AIH	VLD	0.05	0.12800
	AIH	HC	5.96E-06*	0.57498
	LT	VLD	5.90E-12*	9.10E-13*
	LT	HC	1.28E-27*	1.6641E-07*
	VLD	HC	0.03*	0.23352

Table S3: In infection naïve individuals, comparison of Roche anti-RBD antibody response across disease groups at post-V2 and post-V3. Comparisons at post-v3 in AstraZeneca vaccinated individuals not made due to low n numbers. Kruskal Wallis with Dunn's post-hoc test, adjusted for multiple comparisons using Benjamini Hochberg. Cir = Cirrhosis, AIH = Autoimmune hepatitis, LT = Liver transplant, Az = AstraZeneca vaccine. * indicates statistical significance ($P<0.05$)

Disease group	Vaccine platform	Timepoint	Seronegative (%)	Seropositive (%)
Cir	ChAdOx1	Post-V1	4 (12%)	30 (88%)
		Post-V2	-	85 (100%)
		Post-V3	-	6 (100%)
	mRNA	Post-V1	20 (13%)	133 (87%)
		Post-V2	4 (2%)	200 (98%)
		Post-V3	2 (2%)	100 (98%)
LT	ChAdOx1	Post-V1	14 (52%)	13 (48%)
		Post-V2	18 (35%)	34 (65%)
		Post-V3	-	2 (100%)
	mRNA	Post-V1	80 (67%)	39 (33%)
		Post-V2	52 (29%)	127 (71%)
		Post-V3	9 (9%)	86 (91%)
AIH	ChAdOx1	Post-V1	4 (20%)	16 (80%)
		Post-V2	2 (6%)	32 (94%)
		Post-V3	-	3 (100%)
	mRNA	Post-V1	4 (21%)	15 (79%)
		Post-V2	-	33 (100%)
		Post-V3	-	10 (100%)
VLD	mRNA	Post-V1	1 (3%)	28 (97%)
		Post-V2	-	27 (100%)
		Post-V3	-	24 (100%)
HC	ChAdOx1	Post-V1	-	10 (100%)
		Post-V2	-	20 (100%)
		Post-V3	-	-
	mRNA	Post-V1	-	27 (100%)
		Post-V2	-	35 (100%)
		Post-V3	-	23 (100%)

Table S4: In infection naïve individuals, comparison of Roche anti-RBD antibody response rate across disease groups at post-V2 and post-V3. Seropositive defined as >0.8AU/mL by anti-RBD Ig assay. Cir = Cirrhosis, AIH = Autoimmune hepatitis, LT = Liver transplant, VLD = Vascular liver disease; HC = healthy control

	Univariable		Multivariable	
Variable	Odds Ratio (95% CI)	P value	Odds Ratio (95% CI)	P value
Age 45-64	0.61 (0.3 - 1.2)	0.17	0.36 (0.078 - 1.2)	0.14
Age 65-74	0.33 (0.16 - 0.69)	0.0035	0.19 (0.038 - 0.67)	0.017
Age 75+	0.49 (0.2 - 1.2)	0.11	0.38 (0.067 - 1.8)	0.23
Male sex	0.69 (0.44 - 1.1)	0.12	0.96 (0.48 - 1.9)	0.9
ALF	0.98 (0.4 - 2.4)	0.96	-	-
HCC	0.99 (0.54 - 1.8)	0.97	-	-
Decompensation	0.77 (0.49 - 1.2)	0.25	-	-
<2yrs post-transplant	0.52 (0.28 - 0.96)	0.038	0.43 (0.18 - 1)	0.054
mTORi only	0.83 (0.32 - 2.2)	0.7	0.49 (0.12 - 2.2)	0.32
CNI + Other	0.66 (0.38 - 1.2)	0.14	0.56 (0.23 - 1.3)	0.2
CNI + MMF	0.37 (0.22 - 0.63)	0.00029	0.42 (0.19 - 0.93)	0.036
mRNA vaccine	1.3 (0.76 - 2.2)	0.34	1.6 (0.74 - 3.3)	0.23

Table S5: Logistic regression models of anti-RBD seropositivity (>0.8U/mL) in liver transplant recipients following two doses of COVID-19 vaccine. Age is a continuous variable, all other variables are discrete. Age is compared to 18-44 age group. Previous COVID-19 was removed as a variable as 100% of patients with previous COVID-19 had responses >0.8U/mL.

	Univariable		Multivariable	
Variable	Odds Ratio (95% CI)	P value	Odds Ratio (95% CI)	P value
Age 45-64	-0.49 (-1.1 - 0.14)	0.13	-0.31 (-0.9 - 0.28)	0.31
Age 65-74	-0.62 (-1.3 - 0.08)	0.081	-0.55 (-1.2 - 0.12)	0.11
Age 75+	-0.5 (-1.5 - 0.51)	0.32	-0.81 (-1.8 - 0.14)	0.1
Antimetab. +/- other	0.16 (-0.4 - 0.72)	0.56	-0.0093 (-0.55 - 0.53)	0.97
Cirrhosis yes	0.1 (-0.39 - 0.59)	0.68	0.092 (-0.36 - 0.54)	0.69
Corticosteroid +/- other	-0.49 (-1.2 - 0.22)	0.17	-0.46 (-1.2 - 0.23)	0.2
Male sex	0.4 (-0.22 - 1)	0.2	0.36 (-0.24 - 0.97)	0.24
MMF +/- other	-0.45 (-1.2 - 0.27)	0.22	-0.34 (-1 - 0.35)	0.34
mRNA vaccine	0.83 (0.42 - 1.2)	0.00013	0.76 (0.31 - 1.2)	0.0016
No immunosupp.	0.45 (-0.32 - 1.2)	0.25	-0.47 (-1.3 - 0.33)	0.26
Previous COVID-19	1.1 (0.26 - 2)	0.011	1.1 (0.28 - 2)	0.012

Table S6: Linear regression models of log10 anti-RBD in autoimmune hepatitis patients following two COVID-19 vaccine. Age is compared to 18-44year old category. Drugs compared to antimetabolite alone group.

	Univariable		Multivariable	
**Variable**	**Estimate (95% CI)**	**P value**	**Estimate (95% CI)**	**P value**
Age 45-64	-0.27 (-0.64 - 0.11)	0.16	-0.37 (-0.8 - 0.069)	0.098
Age 65-74	-0.32 (-0.7 - 0.069)	0.11	-0.39 (-0.83 - 0.055)	0.086
Age 75+	-0.55 (-1 - -0.088)	0.02	-0.52 (-1 - -0.029)	0.038
Male sex	-0.032 (-0.22 - 0.16)	0.74	0.0087 (-0.19 - 0.2)	0.93
MELD	-0.064 (-0.11 - -0.02)	0.0043	-0.085 (-0.13 - -0.039)	0.00032
CP-B/C	0.042 (-0.15 - 0.23)	0.67	0.071 (-0.16 - 0.3)	0.54
ALD	0.04 (-0.14 - 0.22)	0.67	0.0091 (-0.2 - 0.22)	0.93
HBV	0.1 (-0.25 - 0.45)	0.56	0.0061 (-0.36 - 0.37)	0.97
HCV	-0.1 (-0.32 - 0.11)	0.34	-0.13 (-0.39 - 0.13)	0.33
NAFLD	0.034 (-0.17 - 0.23)	0.74	0.075 (-0.15 - 0.3)	0.5
mRNA Vaccine	0.17 (-0.026 - 0.36)	0.09	0.26 (0.05 - 0.47)	0.015
Previous COVID-19	0.88 (0.63 - 1.1)	1.90E-11	0.9 (0.65 - 1.2)	1.40E-11

Table S7: Linear regression models of log10 transformed anti-RBD antibody (>0.8U/mL) in cirrhosis patients at the Post-V2 timepoint. MELD is a continuous variable, all other variables are discrete. Age is compared to 18-44 year old group. CP = Child's Pugh class, INR = International normalized ratio, CI = Confidence interval.

Supplementary reference

[1] Elbe S, Buckland-Merrett G. Data, disease and diplomacy: GISAID's innovative contribution to global health. Global Challenges 2017;1:33-46.
