# Supplementary material for: Immune responses and clinical outcomes after COVID-19 vaccination in patients with liver disease and liver transplant recipients
Source: J Hepatol. 2024 Jan;80(1):109–23. doi: 10.1016/j.jhep.2023.10.009 (PMC10914634; doi:10.1016/j.jhep.2023.10.009)
Supplement: Multimedia component 2 — : [file mmc2.docx]

**Journal of Hepatology**

**CTAT methods**

Tables for a “Complete, Transparent, Accurate and Timely account” (CTAT) are now mandatory for all revised submissions. The aim is to enhance the reproducibility of methods.

- Only include the parts relevant to your study
- Refer to the CTAT in the main text as ‘Supplementary CTAT Table’
- Do not add subheadings
- Add as many rows as needed to include all information
- Only include one item per row

**If the CTAT form is not relevant to your study, please outline the reasons why:**

|  |
| --- |

- 1. **Antibodies**

| **Name** | **Citation** | **Supplier** | **Cat no.** | **Clone no.** |
| --- | --- | --- | --- | --- |
| Human IFNy ELISpot basic kit |  | Mabtech | 3420-2A | 1-D1K 7-B6-1, biotin Streptavidin-ALP |

- 1. **Cell lines**

| **Name** | **Citation** | **Supplier** | **Cat no.** | **Passage no.** | **Authentication test method** |
| --- | --- | --- | --- | --- | --- |
| N/A |  |  |  |  |  |

- 1. **Organisms**

| **Name** | **Citation** | **Supplier** | **Strain** | **Sex** | **Age** | **Overall n number** |
| --- | --- | --- | --- | --- | --- | --- |
| N/A |  |  |  |  |  |  |

- 1. **Sequence based reagents**

| **Name** | **Sequence** | **Supplier** |
| --- | --- | --- |
| N/A |  |  |

- 1. **Biological samples**

| **Description** | **Source** | **Identifier** |
| --- | --- | --- |
| Human peripheral blood mononuclear cells (PBMCs) | University Medical Center Hamburg-Eppendorf; Foundation IRCCS Ca’ Granda Ospedale Maggiore Policlinico (PolImmuneCOVID study); University of Padova; University of Barcelona. UK OCTAVE study; UK PITCH study | **N/A** |
| Human plasma/serum samples | University Medical Center Hamburg-Eppendorf; Foundation IRCCS Ca’ Granda Ospedale Maggiore Policlinico (PolImmuneCOVID study); University of Padova; University of Barcelona. UK OCTAVE study; UK PITCH study | **N/A** |

- 1. **Deposited data**

| **Name of repository** | **Identifier** | **Link** |
| --- | --- | --- |
| GISAID EpiCoV^TM^ | Total submissions by Clade/lineage and variants | https://gisaid.org/ |

- 1. **Software**

| **Software name** | **Manufacturer** | **Version** |
| --- | --- | --- |
| R |  | V4.2.1 |
| RStudio |  | 2023.03.1+446 |
| GraphPad Prism | GraphPad Software, LLC. | v9.4.0 |
| CTL Immunocapture | Cellular technology limited | 2.7.3 |
|  |  |  |
|  |  |  |
|  |  |  |

- 1. **Other (e.g. drugs, proteins, vectors etc.)**

| Wild-type SARS-CoV-2 Spike | Wuhan A | MesoScale Discovery K15668U |
| --- | --- | --- |
| SARS-CoV-2 Spike B.1.1.529; BA.1; BA.1.15 | Mutations compared to wild-type SARS-CoV-2 spike: A67V, ∆H69-V70, T95I, G142D, ∆143-145, ∆211/L212I, ins214EPE, G339D, S371L, S373P, S375F, K417N, N440K, G446S, S477N, T478K, E484A, Q493R, G496S, Q498R, N501Y, Y505H, T547K, D614G, H655Y, N679K, P681H, N764K, D796Y, N856K, Q954H, N969K, L981F | MesoScale Discovery K15668U |
| SARS-CoV-2 Spike XBB.1 | Mutations compared to wild-type SARS-CoV-2 spike: T19I, L24-A27>S, V83A, G142D, Y144del, H146Q, Q183E, V213E, G252V, G339H, R346T, L368I, S371F, S373P, S375F, T376A, D405N, R408S, K417N, N440K, V445P, G446S, N460K, S477N, T478K, E484A, F486S, F490S, Q498R, N501Y, Y505H, D614G, H655Y, N679K, P681H, N764K, D796Y, Q954H, N969K | MesoScale Discovery K15668U |
| SARS-CoV-2 Spike BF.7 | Mutations compared to wild-type SARS-CoV-2 spike: T19I, L24-A27>S, H69-V70del, G142D, V213G, G339D, R346T, S371F, S373P, S375F, T376A, D405N, R408S, K417N, N440K, L452R, S477N, T478K, E484A, F486V, Q498R, N501Y, Y505H, D614G, H655Y, N679K, P681H, N764K, D796Y, Q954H, N969K | MesoScale Discovery K15668U |
| SARS-CoV-2 Spike BA.2.75.2 | Mutations compared to wild-type SARS-CoV-2 spike: T19I, L24-A27>S, G142D, K147E, W152R, F157L, I210V, V213G, G257S, G339H, R346T, S371F, S373P, S375F, T376A, D405N, R408S, K417N, N440K, G446S, N460K, S477N, T478K, E484A, F486S, Q498R, N501Y, Y505H, D614G, H655Y, N679K, P681H, N764K, D796Y, Q954H, N969K, D1199N | MesoScale Discovery K15668U |
| SARS-CoV-2 Spike BQ.1.1 | Mutations compared to wild-type SARS-CoV-2 spike: T19I, L24-A27>S, H69-V70del, G142D, V213G, G339D, R346T, S371F, S373P, S375F, T376A, D405N, R408S, K417N, N440K, K444T, L452R, N460K, S477N, T478K, E484A, F486V, Q498R, N501Y, Y505H,D614G, H655Y, N679K, P681H, N764K, D796Y, Q954H, N969K | MesoScale Discovery K15668U |
| SARS-CoV-2 Spike BA.2.75 | Mutations compared to wild-type SARS-CoV-2 spike: T19I, L24-A27>S, G142D, K147E, W152R, F157L, I210V, V213G, G257S, G339H, S371F, S373P, S375F, T376A, D405N, R408S, K417N, N440K, G446S, N460K, S477N, T478K, E484A, Q498R, N501Y, Y505H, D614G, H655Y, N679K, P681H, N764K, D796Y, Q954H, N969K | MesoScale Discovery K15668U |
| SARS-CoV-2 Spike BA.4.6 | Mutations compared to wild-type SARS-CoV-2 spike: V3G, T19I, L24-A27>S, H69-V70del, G142D, V213G, G339D, R346T, S371F, S373P, S375F, T376A, D405N, R408S, K417N, N440K, L452R, S477N, T478K, E484A, F486V, Q498R, N501Y, Y505H, D614G, H655Y, N658S, N679K, P681H, N764K, D796Y, Q954H, N969K | MesoScale Discovery K15668U |
| SARS-CoV-2 Spike BQ.1 | Mutations compared to wild-type SARS-CoV-2 spike: T19I, L24-A27>S, H69-V70del, G142D, V213G, G339D, S371F, S373P, S375F, T376A, D405N, R408S, K417N, N440K, K444T, L452R, N460K, S477N, T478K, E484A, F486V, Q498R, N501Y, Y505H, D614G, H655Y, N679K, P681H, N764K, D796Y, Q954H, N969K | MesoScale Discovery K15668U |
| SARS-CoV-2 Spike BA.5 | Mutations compared to wild-type SARS-CoV-2 spike: T19I, (L24-A27)toS, del69/70, G142D, V213G, G339D, S371F, S373P, S375F, T376A, D405N, R408S, K417N, N440K, L452R, S477N, T478K, E484A, F486V, Q498R, N501Y, Y505H, D614G, H655Y, N679K, P681H, N764K, D796Y, Q954H, N969K | MesoScale Discovery K15668U |
| Wild-type SARS-CoV-2 RBD | SARS-CoV-2 S1 RBD - Wuhan A | MesoScale Discovery K15679U |
| SARS-CoV-2 RBD B.1.1.529; BA.1; BA.1.15 | Mutations compared to Wuhan A RBD: G339D, S371L, S373P, S375F, K417N, N440K, G446S, S477N, T478K, E484A, Q493R, G496S, Q498R, N501Y, Y505H | MesoScale Discovery K15679U |
| SARS-CoV-2 RBD BQ.1.1 | Mutations compared to Wuhan A RBD: G339D, R346T, S371F, S373P, S375F, T376A, D405N, R408S, K417N, N440K, K444T, L452R, N460K, S477N, T478K, E484A, F486V, Q498R, N501Y, Y505H | MesoScale Discovery K15679U |
| SARS-CoV-2 RBD BA.2.75.2 | Mutations compared to Wuhan A RBD: G339H, R346T, S371F, S373P, S375F, T376A, D405N, R408S, K417N, N440K, G446S, N460K, S477N, T478K, E484A, F486S, Q498R, N501Y, Y505H | MesoScale Discovery K15679U |
| SARS-CoV-2 RBD BA.4.6;BF.7 | Mutations compared to Wuhan A RBD: G339D, R346T, S371F, S373P, S375F, T376A, D405N, R408S, K417N, N440K, L452R, S477N, T478K, E484A, F486V, Q498R, N501Y, Y505H | MesoScale Discovery K15679U |
| SARS-CoV-2 RBD XBB.1 | Mutations compared to Wuhan A RBD: G339H, R346T, L368I, S371F, S373P, S375F, T376A, D405N, R408S, K417N, N440K, V445P, G446S, N460K, S477N, T478K, E484A, F486S, F490S, Q498R, N501Y, Y505H | MesoScale Discovery K15679U |
| SARS-CoV-2 RBD BA.4;BA.5 | Mutations compared to Wuhan A RBD: G339D, S371F, S373P, S375F, T376A, D405N, R408S, K417N, N440K, L452R, S477N, T478K, E484A, F486V, Q498R, N501Y, Y505H | MesoScale Discovery K15679U |
| SARS-CoV-2 RBD BA.2.75 | Mutations compared to Wuhan A RBD: G339H, S371F, S373P, S375F, T376A, D405N, R408S, K417N, N440K, G446S, N460K, S477N, T478K, E484A, Q498R, N501Y, Y505H | MesoScale Discovery K15679U |
| SARS-CoV-2 RBD BQ.1 | Mutations compared to Wuhan A RBD: G339D, S371F, S373P, S375F, T376A, D405N, R408S, K417N, N440K, K444T, L452R, N460K, S477N, T478K, E484A, F486V, Q498R, N501Y, Y505H | MesoScale Discovery K15679U |

- 1. **Please provide the details of the corresponding methods author for the manuscript:**

| Eleanor Barnes, ellie.barnes@ndm.ox.ac.uk |
| --- |

**2.0 Please confirm for randomised controlled trials all versions of the clinical protocol are included in the submission. These will be published online as supplementary information.**

|  |
| --- |
