# Supplementary material for: Immune responses and clinical outcomes after COVID-19 vaccination in patients with liver disease and liver transplant recipients
Source: J Hepatol. 2024 Jan;80(1):109–23. doi: 10.1016/j.jhep.2023.10.009 (PMC10914634; doi:10.1016/j.jhep.2023.10.009)
Supplement: Multimedia component 4 [file mmc4.pdf]

JOURNAL OF HEPATOLOGY

Immune responses and clinical outcomes after COVID-19 vaccination in patients with liver disease and liver transplant recipients

Sam M. Murray[1], Elisa Pose[2,3], Melanie Wittner[4,5], Maria-Carlota Londoño[2,3], Golda Schaub[4,5], Jonathan Cook[6], Stavros Dimitriadis[7], Georgina Meacham[7], Sophie Irwin[7], Zixiang Lim[7], Paul Duengelhoef[5], Martina Sterneck[5], Ansgar W. Lohse[4,5], Valeria Perez[2,3,8], Palak Trivedi[9,10], Khush Bhandal[10], Benjamin H. Mullish[11,12], Pinelopi Manousou[11,12], Nicholas M. Provine[7], Emma Avitabile[2,3], Miles Carroll[13], Tom Tipton[13], Saoirse Healy[13], Patrizia Burra[14], Paul Klenerman[1,7,15], Susanna Dunachie[1,15,16], Barbara Kronsteiner[1,15,16], Agnieszka Katarzyna Maciola[17], Giulia Pasqual[17,18], Virginia Hernandez-Gea[2,3,8], Juan Carlos Garcia-Pagan[2,3,8], Pietro Lampertico[19,20], Massimo Iavarone[19], Pere Gines[2,3], Marc Lütgehetmann[5,21], Julian Schulze zur Wiesch[4,5], Francesco Paolo Russo[14], Eleanor Barnes[1,15,*,†,‡], Thomas Marjot[22,23,*,†,‡], on behalf of the OCTAVE Collaborative Group, PITCH study, and the EASL supported COVID-Hep vaccine network[+]

Journal of Hepatology **2024**. vol. 80 | 109–123

Check for updates

Background & Aims: Comparative assessments of immunogenicity following different COVID-19 vaccines in patients with distinct liver diseases are lacking. SARS-CoV-2-specific T-cell and antibody responses were evaluated longitudinally after one to three vaccine doses, with long-term follow-up for COVID-19-related clinical outcomes.
Methods: A total of 849 participants (355 with cirrhosis, 74 with autoimmune hepatitis [AIH], 36 with vascular liver disease [VLD], 257 liver transplant recipients [LTRs] and 127 healthy controls [HCs]) were recruited from four countries. Standardised immune assays were performed pre and post three vaccine doses (V1-3).
Results: In the total cohort, there were incremental increases in antibody titres after each vaccine dose ($p < 0.0001$). Factors associated with reduced antibody responses were age and LT, whereas heterologous vaccination, prior COVID-19 and mRNA platforms were associated with greater responses. Although antibody titres decreased between post-V2 and pre-V3 ($p = 0.012$), patients with AIH, VLD, and cirrhosis had equivalent antibody responses to HCs post-V3. LTRs had lower and more heterogenous antibody titres than other groups, including post-V3 where 9% had no detectable antibodies; this was heavily influenced by intensity of immunosuppression. Vaccination increased T-cell IFNγ responses in all groups except LTRs. Patients with liver disease had lower functional antibody responses against nine Omicron subvariants and reduced T-cell responses to Omicron BA.1-specific peptides compared to wild-type. 122 cases of breakthrough COVID-19 were reported of which 5/122 (4%) were severe. Of the severe cases, 4/5 (80%) occurred in LTRs and 2/5 (40%) had no serological response post-V2.
Conclusion: After three COVID-19 vaccines, patients with liver disease generally develop robust antibody and T-cell responses to vaccination and have mild COVID-19. However, LTRs have sustained no/low antibody titres and appear most vulnerable to severe disease.

© 2023 The Author(s). Published by Elsevier B.V. on behalf of European Association for the Study of the Liver. This is an open access article under the CC BY license (http://creativecommons.org/licenses/by/4.0/).

Introduction

The rapid development and deployment of vaccinations against SARS-CoV-2, alongside a degree of naturally acquired immunity from past infection, has transformed the landscape of the COVID-19 pandemic. At a population level, vaccination has been shown to reduce SARS-CoV-2 infection and protect against hospitalisation and death from severe COVID-19.[1,2] However, understanding the immunogenicity and effectiveness of vaccination programmes in vulnerable cohorts with functional or pharmacological immunosuppression remains an important clinical priority.[3] This is particularly relevant given the continuing emergence of novel viral variants of concern (VoC). In the pre-vaccination era, patients with a range of liver conditions were shown to be at increased risk of SARS-CoV-2

Keywords: COVID-19; Vaccination; Liver transplantation; Autoimmune hepatitis; Cirrhosis; Vascular liver disease; T cells; Antibodies; SARS-CoV-2; Variants of Concern.
Received 9 June 2023; received in revised form 20 September 2023; accepted 3 October 2023; available online 19 October 2023
* Corresponding authors. Addresses: Nuffield Department of Medicine, University of Oxford, OUH Hospital NHS Trust, Oxford, OX3 9DU, UK; Tel.: 01865 281547. (E. Barnes), or Wellcome Trust Clinical Research Training Fellow, Oxford Liver Unit, Oxford University Hospitals NHS Foundation Trust, John Radcliffe Hospital, Oxford, OX3 9DU, UK.
E-mail addresses: ellie.barnes@ndm.ox.ac.uk (E. Barnes), thomas.marjot@ndm.ox.ac.uk (T. Marjot).
† Shared senior authors
‡ Shared corresponding authors
+ Consortia participants listed in the supplementary information.
https://doi.org/10.1016/j.jhep.2023.10.009

infection and severe COVID-19. This included high rates of mortality in cohorts with cirrhosis, greater intensive care unit requirements in liver transplant recipients (LTR), and an elevated risk of SARS-CoV-2 infection and severe complications in patients with vascular liver disease (VLD).[4–7] Epidemi ological studies have since demonstrated a benefit of COVID-19 vaccination in some of these groups. This includes retro spective analysis of electronic health record data showing that three doses of mRNA vaccination are associated with a significant reduction in rates of SARS-CoV-2 infection and severe COVID-19 in patients with cirrhosis.[8] In addition, there have been several studies exploring immune responses to vaccination in liver cohorts.[9–11] However, these studies have often examined responses after only one or two vaccine doses and overall conclusions have been hampered by small sample sizes, differing sampling timepoints, cross-sectional design, heterogeneity in laboratory assays, absence of VoC analysis, and lack of healthy control datasets. As a result, considerable uncertainty remains within the literature regarding vaccine immunogenicity across the various vaccine platforms and liver disease phenotypes.[10] Data relating to T-cell responses after vaccination are also lacking, except for some small, detailed studies in individual liver groups.[12] This requires further investigation in patients with liver disease given that cellular responses are an important correlate of protection against severe COVID-19.[13,14] Lastly, to date, studies have tended to be single-centre and have interrogated solitary vaccine types which limits their widespread generalisability.

As a result, we sought to deliver a European multicentre prospective cohort study evaluating T-cell and antibody responses following COVID-19 vaccination in LTRs, patients with cirrhosis, autoimmune hepatitis (AIH), or VLD, and healthy controls (HCs). Herein, we performed longitudinal immunological assessments in these groups using standardised assays at multiple timepoints following up to three doses of differing COVID-19 vaccine regimens. It also involved assessments of vaccine responses to some of the most up-to-date viral variants including multiple sublineages of Omicron. In addition, patients were followed long-term to establish the occurrence and severity of breakthrough SARS-CoV-2 infection after vaccination.

Patients and methods

Study design and sampling protocol

We evaluated the humoral and cellular immune response after COVID-19 vaccination delivered as part of clinical care to European patients with cirrhosis, AIH, or VLD, or LTRs. Patients were prospectively recruited across two multicentre consortia; the EASL supported COVID-Hep vaccine network and the UK OCTAVE (Observational Cohort trial T-cells, Antibodies and Vaccine Efficacy in SARS-CoV-2) study. Both studies had identical inclusion criteria; >16 years of age, eligibility for COVID-19 vaccination with BNT162b2, mRNA-1273, or ChAdOx1 nCoV-19 (AZD1222, ChAdOx1) vaccine platforms, and an anticipated life expectancy >6 months. Healthy controls were recruited via the UK PITCH (Protective Immunity from T Cells in Healthcare workers) consortium. All studies included clinical and demographic data collection at baseline followed by longitudinal collection of serum and peripheral blood mononuclear cells (PBMCs) at standardised timepoints throughout the vaccination schedule, although sample availability at each timepoint was variable. A subset of data included in this work has been previously reported.[14–16] However, these studies included: i) patients who had received a single vaccine type such that comparisons of immunogenicity and vaccine effectiveness between vaccine types could not be made,[16] ii) patients who received only two vaccine doses and no booster vaccines[14,15] and iii) did not include any data on SARS-CoV-2 infection rates and disease severity data.[15,16]

Ethical and regulatory approvals

All centres involved in the EASL supported COVID-Hep vaccine registry recruited participants through local ethics approvals. All studies were conducted in compliance with relevant ethical regulations for work with human participants according to the principles of the Declaration of Helsinki (2008) and written informed consent was obtained for all included participants. Additional methods are available in the supplementary materials.

Outcome measures and sample size calculations

The two primary outcomes of this study were levels of anti-SARS-CoV-2 IgG antibodies and the magnitude of the T-cell responses to wild-type (WT) SARS-CoV-2 peptides following COVID-19 vaccination. The study was powered to detect a difference in SARS-CoV-2 IgG anti-S antibody titres 28 days after V2 between disease cohorts (cirrhosis, AIH, VLD, LTR) and HCs. Sample size calculations were performed *a priori* based on available data at the time of study conception relating to COVID-19 vaccine immunogenicity in phase III trials and historic data showing diminished vaccine responses to a range of other viruses in LTRs and patients with cirrhosis. To allow for comparisons between vaccine platforms and assuming a 20% reduction in antibody titres in patients relative to controls we estimated that at least 100 patients per disease group would be required to detect a difference with 90% power and an alpha of 0.05. Secondary outcomes included the magnitude of the T-cell responses specifically to SARS-CoV-2 Omicron BA.1 peptides, IgG binding and inhibition of angiotensin-converting enzyme 2 (ACE2) binding to SARS-CoV-2 VoC, and rates and severity of breakthrough SARS-CoV-2 infection after COVID-19 vaccination.

Clinical phenotyping and definitions

Clinical data for all participants were uploaded electronically from participating sites using REDCap (Research Electronic Data Capture) databases hosted by the University of Oxford or University of Birmingham, UK. Clinical data comprised information on demographics, vaccination type, comorbidities, and disease-specific phenotyping including Child-Pugh (CP) class and aetiology of cirrhosis, and type and dose of immunosuppression for patients with AIH and LTRs. Previous SARS-CoV-2 infection was defined as a patient-reported episode of confirmed COVID-19 or a positive antibody or T-cell response to SARS-CoV-2 nucleocapsid antigen at the first timepoint at which a patient was sampled. Nucleocapsid antibody titres were additionally assessed at post-V1, and post-V3 timepoints. Model for end-stage liver disease (MELD) score

included serum sodium, creatinine, bilirubin and international normalised ratio.[17]

Anti-SARS-CoV-2 Ig analysis

For all participants, the magnitude of anti-SARS-CoV-2 antibodies was measured using identical Roche Elecsys® Anti-SARS-CoV-2-S (anti-S) and Roche Elecsys® Anti-SARS-CoV-2-N (anti-N) assays. The Roche Anti-SARS-CoV-2-S assay measures the presence and the amount of serum antibodies to the spike receptor binding domain (RBD) antigen of SARS-CoV-2. Seroconversion was manufacturer defined as anti-S antibodies ≥0.8 U/ml and anti-N antibodies >1U/ml. The upper limit of detection for anti-S antibodies was 25,000 U/ml. Low response was defined as <380 U/ml as per.[14] Assays for all samples were completed at the University of Hamburg or the UKHSA Laboratories at Porton Down.

Anti-SARS-CoV-2 VoC IgG binding and ACE2 inhibition

IgG titres and ACE2 inhibition were measured against the spike or RBD of WT SARS-CoV-2 and the nine most prevalent Omicron subvariants as of February 2023 (B.1.1.529/BA.1/BA.1.15, BA.2.75, BA.2.75.2, BA.4.6, BA.5, BF.7, BQ.1, BQ.1.1, and XBB.1), using multiplexed Meso Scale Discovery immunoassay panels 32 and 33 (K15668U and K15679U). Assays were performed as per manufacturer recommendations in a subset of patients and HCs, who were selected to include a range of post-V2 anti-RBD Ig titres. Additional methods are available in the supplementary materials.

IFNγ T-cell ELISpot assay

IFNγ ELISpots were performed using Human IFNγ ELISpot Basic kit (Mabtech) as previously described.[18] Wells included 200,000 thawed PBMCs and stimulation conditions included overlapping peptide pools (18 mers with 10 amino acid overlap), negative control (DMSO only) or positive control (CEF and concanavalin A). IFNγ ELISpots for liver disease groups were all performed at one laboratory at the University of Oxford. IFNy ELISpots for healthy controls were performed separately at the University of Oxford. Samples were only included in the analysis if the IFNγ response to DMSO was <50 spot-forming units (SFU)/10^6 PBMCs and positive responses were detected in the positive controls. Additional methods are available in the supplementary materials.

Breakthrough SARS-CoV-2 infection after COVID-19 vaccination

Information regarding breakthrough SARS-CoV-2 infection after vaccination was also collected by screening electronic hospital records and/or contacting individual patients and included date of infection and COVID-19 severity. Data was collected up until December 2022. Severe COVID-19 was defined according to the World Health Organisation classification or based on hospitalisation status.[19] Additional methods are available in the supplementary materials.

Statistical methods

Descriptive statistics are presented as n (%) or median (IQR), unless otherwise indicated. A nominal 2-sided 5% significance level was adopted unless otherwise stated. Kruskal-Wallis test followed by *post hoc* Dunn's testing with Holm-Bonferroni multiplicity adjustment was used for multiple pairwise comparisons. Mann-Whitney *U* test, Wilcoxon matched-paired signed rank test, Pearson's Chi-Square, and Fisher's exact test were used where required as per figure legends. Multiple comparisons corrections were made as per individual figure legends (Holm-Bonferroni adjustments). Spearman's correlations or linear regressions of $\log^{10}$ transformed values investigated relationships between variables of interest. Spearman's correlations were used to quantify the association between variables and linear regression models were used to model effects of clinical or immunological factors on anti-RBD Ig responses. Multivariable models (linear or logistic as per legends) were used to model multivariate effects of clinical and immunological factors on $\log^{10}$ transformed anti-RBD Ig responses (linear) or odds ratio of seropositivity (>0.8 AU/ml, logistic) at post-v2 in the whole cohort or in specific disease groups. Models were created as per figure legends. Statistical analyses were performed using R (v4.2.1) or in GraphPad Prism (v9.4.0). Figures were prepared using R (v4.2.2 (2022-10-31)) with RStudio 2023.03.1+446 or in GraphPad Prism (v9.4.0). R packages used included: ggplot2, rstatix, Hmisc, gtsummary, lme4.

Results

Cohort characteristics

Between March and September 2021, a total of 849 individuals (722 patients with liver disease and 127 HCs) were prospectively recruited from the UK, Italy, Germany, and Spain. The liver disease patient cohort comprised 355 (49%) individuals with cirrhosis, 257 (36%) LTRs, 74 (10%) individuals with AIH, and 36 (5%) with VLD. In the entire cohort, the primary two-dose vaccination course included ChAdOx1 (n = 246), BNT162b2 (n = 460), and mRNA-1273 (n = 118). An additional 13 individuals received heterologous vaccination with ChAdOx1 for first vaccination followed by an mRNA platform (BNT162b2 or mRNA-1273) for the second vaccination. Data were available for 307 participants after a third vaccination which included 187 (61%) receiving BNT162b2, 110 (36%) receiving mRNA-1273, and 10 (3%) where the vaccine type was unknown.

Across the entire liver disease cohort, the median age was 60 years (IQR 52–68), 425 (59%) were male, and 76 (11%) had previous evidence of SARS-CoV-2 infection. In the HC cohort, the median age was 36 years (IQR 25–45), 40 (31%) were male, and 40 (31%) had evidence of previous SARS-CoV-2 infection. Nineteen patients became newly positive for nucleocapsid antibody at the post-V3 timepoint. Clinical information for each specific disease category is presented in Table 1. For the analysis, 21 (29%) patients with AIH and concurrent cirrhosis were included in the AIH group and not the cirrhosis group.

Antibody responses

Antibody responses across liver disease phenotypes and vaccine platforms

Longitudinal assessment of antibody responses using the Roche anti-RBD assay across all liver disease phenotypes and HCs is presented in Fig. 1A. Prior SARS-CoV-2 infection was

Table 1. Clinical characteristics.

	LT (n = 257)	AIH (n = 74)	Cirr (n = 355)	VLD (n = 36)	HC (n = 127)	Total (N = 849)
Age (years, IQR)	60 (50-67)	61 (49-69)	62 (55-69)	46 (40-49)	36 (25-45)	58 (46-66)
Unknown	2 (0.8%)	0 (0%)	2 (0.6%)	0 (0%)	1 (0.8%)	5 (0.5%)
Sex						
Female	98 (38%)	61 (82%)	123 (35%)	15 (42%)	86 (68%)	383 (45%)
Male	159 (62%)	13 (18%)	232 (65%)	21 (58%)	40 (31%)	465 (55%)
Unknown	0 (0%)	0 (0%)	0 (0%)	0 (0%)	1 (0.8%)	1 (0.1%)
Ethnicity						
Asian	1 (0.4%)	5 (6.8%)	7 (2.0%)	0 (0%)	16 (13%)	29 (3.4%)
Black	2 (0.8%)	0 (0%)	8 (2.3%)	1 (2.8%)	1 (0.8%)	12 (1.4%)
Other	3 (1.2%)	2 (2.7%)	7 (2.0%)	4 (11%)	4 (3.1%)	20 (2.4%)
White	132 (51%)	65 (88%)	293 (83%)	31 (86%)	84 (66%)	605 (71%)
Unknown	119 (46%)	2 (2.7%)	40 (11%)	0 (0%)	22 (17%)	183 (22%)
Obesity						
No	156 (61%)	51 (69%)	220 (62%)	31 (86%)	82 (65%)	540 (64%)
Yes	49 (19%)	14 (19%)	116 (33%)	4 (11%)	5 (3.9%)	188 (22%)
Unknown	52 (20%)	9 (12%)	19 (5.4%)	1 (2.8%)	40 (31%)	121 (14%)
Smoking status						
Never smoked	227 (88%)	44 (59%)	188 (53%)	25 (69%)	117 (92%)	601 (71%)
Previously smoked	28 (11%)	22 (30%)	107 (30%)	2 (5.6%)	10 (7.9%)	169 (20%)
Currently smoke	2 (0.8%)	8 (11%)	60 (17%)	9 (25%)	0 (0%)	79 (9.3%)
Diabetes						
Yes	25 (9.7%)	10 (14%)	102 (29%)	2 (5.6%)	0 (0%)	139 (17%)
Hypertension						
Yes	87 (34%)	12 (16%)	136 (38%)	0 (0%)	3 (2.9%)	238 (29%)
Prior SARS-CoV-2 infection						
No confirmed infection	241 (94%)	68 (92%)	307 (86%)	30 (83%)	87 (69%)	733 (86%)
Previously infected	16 (6.2%)	6 (8.2%)	48 (14%)	6 (17%)	40 (31%)	116 (14%)
Vaccine type - dose 1						
ChAdOx1 nCoV-19	73 (28%)	38 (51%)	112 (32%)	0 (0%)	39 (31%)	262 (31%)
BNT162b2	175 (68%)	23 (31%)	176 (50%)	0 (0%)	87 (69%)	461 (54%)
mRNA-1273	7 (2.7%)	13 (18%)	64 (18%)	36 (100%)	1 (0.8%)	121 (14%)
Unknown	2 (0.8%)	0 (0%)	3 (0.8%)	0 (0%)	0 (0%)	5 (0.6%)
Vaccine type - dose 2						
ChAdOx1 nCoV-19	66 (26%)	37 (50%)	104 (29%)	0 (0%)	39 (31%)	246 (29%)
BNT162b2	180 (70%)	22 (30%)	180 (51%)	0 (0%)	87 (69%)	469 (55%)
mRNA-1273	9 (3.5%)	14 (19%)	64 (18%)	33 (92%)	1 (0.8%)	121 (14%)
Unknown	2 (0.8%)	1 (1.4%)	7 (2.0%)	3 (8.3%)	0 (0%)	13 (1.5%)
Vaccine type - dose 3*						
BNT162b2	88 (85%)	4 (24%)	70 (52%)	0 (0%)	25 (89%)	187 (61%)
mRNA-1273	9 (8.7%)	13 (76%)	61 (46%)	24 (100%)	3 (11%)	110 (36%)
Unknown	7 (6.7%)	0 (0%)	3 (2.2%)	0 (0%)	0 (0%)	10 (3.3%)
<2 years post LT						
Yes	37 (14%)	—	—	—	—	—
Indication for LT						
Acute liver failure	21 (8%)	—	—	—	—	—
Decompensated cirrhosis	99 (39%)	—	—	—	—	—
HCC	33 (13%)	—	—	—	—	—
Other	40 (16%)	—	—	—	—	—
Unknown	64 (25%)	—	—	—	—	—
IS						
Azathioprine	16 (6.2%)	21 (29%)	2 (0.6%)	—	—	—
Sirolimus	6 (2.3%)	0 (0%)	—	—	—	—
Everolimus	44 (17%)	0 (0%)	—	—	—	—
6-MP	5 (1.9%)	27 (37%)	1 (0.3%)	—	—	—
MMF	79 (31%)	9 (12%)	1 (0.3%)	1 (3%)	—	—
MTX	—	—	2 (0.6%)	—	—	—
Corticosteroids	50 (19%)	33 (43%)	1 (0.3%)	1 (3%)	—	—
Ciclosporin	41 (16%)	0 (0%)	—	—	—	—
Tacrolimus	195 (76%)	6 (8.2%)	—	—	—	—
No. IS therapies						
0	3 (1.2%)	8 (11%)	—	—	—	—
1	95 (37%)	36 (49%)	—	—	—	—
2	132 (51%)	23 (32%)	—	—	—	—
3	27 (11%)	6 (8.2%)	—	—	—	—
IS combinations						
CNI only	81 (32%)	—	—	—	—	—
mTori only	13 (5.1%)	—	—	—	—	—

(*continued on next page*)

Table 1. (*continued*)

	LT (n = 257)	AIH (n = 74)	Cirr (n = 355)	VLD (n = 36)	HC (n = 127)	Total (N = 849)
CNI + MMF (+/- other)	79 (31%)	–	–	–	–	–
CNI + other (+/- other)	81 (32%)	–	–	–	–	–
Cirrhosis severity						
MELD score (median, IQR)	–	–	7.25 (6.67, 8.43)	–	–	–
Unknown	–	–	60 (17%)	–	–	–
Child-Pugh class						
A	–	19 (26%)	232 (65%)	–	–	–
B	–	2 (2.7%)	82 (23%)	–	–	–
C	–	1 (1.4%)	31 (8.7%)	–	–	–
No cirrhosis	–	52 (70%)	0 (0%)	–	–	–
Unknown	–	–	10 (2.8%)	–	–	–
Cirrhosis aetiology						
NAFLD	–	1 (5%)	98 (28%)	–	–	–
ALD	–	1 (5%)	152 (43%)	–	–	–
HCV	–	2 (9%)	80 (22%)	–	–	–
HBV	–	1 (5%)	24 (6.7%)	–	–	–
PBC	–	2 (9%)	12 (2.8%)	–	–	–
PSC	–	2 (9%)	12 (3.1%)	–	–	–
Unknown	–	15 (68%)	33 (9.3%)	–	–	–
VLD aetiology						
NCPVT	–	–	–	16 (44%)	–	–
BCS	–	–	–	9 (25%)	–	–
PSVD	–	–	–	11 (31%)	–	–

6-MP, 6-mercaptopurine; AIH, autoimmune hepatitis; ALD, alcohol-related liver disease; BCS, Budd-Chiari syndrome; Cirr, cirrhosis; CNI, calcineurin inhibitor; HCs, healthy controls; HCC, hepatocellular carcinoma; IS, immunosuppression; LT, liver transplant; MMF, mycophenolate mofetil; mTORi, mTOR inhibitor; MTX, methotrexate; NAFLD, non-alcoholic fatty liver disease; NCPVT, non-cirrhotic non-tumoral portal vein thrombosis; PBC, primary biliary cholangitis; PSC, primary sclerosing cholangitis; PSVD, porto-sinusoidal vascular disorder; VLD, vascular liver disease. *Numbers only given for individuals with third dose anti-RBD Ig titre data available.

associated with a significant increase in anti-RBD titres across all disease groups (Fig. S1) and therefore previously infected individuals were removed from the primary analysis (n = 76 with liver disease and n = 40 HCs excluded).

Across the total liver disease cohort, there was a stepwise incremental increase in median antibody titres after each consecutive vaccine dose (6.24 U/ml [0.4–44.9] post-V1 *vs.* 846 U/ml [158–2,653] post-V2 *vs.* 12,746 U/ml [2,508–25,000] U/ml post-V3; $p < 0.0001$). This observation remained significant after excluding 19 patients who had become newly positive for nucleocapsid antibody between enrolment and post-V3 (Fig. S1B). A decrease in antibody titre between the post-V2 and pre-V3 timepoint was also observed in both liver disease and HC groups and was subsequently boosted by the administration of a third vaccine dose (Table S1). mRNA platforms were associated with significantly higher post-V2 anti-RBD titres compared to ChAdOx1 in both liver disease (953 [158–3,214] mRNA *vs.* 593 U/ml [114–1521] ChAdOx1; p = 0.0005) and HC cohorts (15,634 U/ml [10,829–21,445] mRNA *vs.* 1,198 U/ml [855–1,546] ChAdOx1; $p < 0.001$). Thirteen patients who received heterologous first and second vaccines had significant elevations in post-V2 antibody titres compared to homologous ChAdOx1 (p = 0.0002) and homologous mRNA regimens (p = 0.004) (Fig. 1B). In cohorts where data were available for both BNT162b2 or mRNA-1273 vaccinated individuals, there were no significant differences in anti-RBD at the post-V2 timepoint; therefore, both mRNA vaccines were grouped for further analysis (Fig. S2). Within the entire study cohort (patients and HCs) multivariable analyses showed that the factors significantly associated with lower antibody response after V2 were advancing age and inclusion in the LTR group, and factors associated with greater response were mRNA vaccination, heterologous vaccination (V1 + V2) and previous COVID-19 infection (Fig. 1C, Table S2).

Our study allowed us to compare antibody responses relating to specific combinations of disease and vaccine types. At the post-V2 and post-V3 timepoints LTRs mounted lower antibody titres compared to all other disease groups and HCs regardless of vaccine type (Table S3). mRNA-vaccinated patients with cirrhosis had reduced post-V2 antibody titres compared to mRNA-vaccinated HCs, but ChAdOx1-vaccinated patients with cirrhosis had comparable post-V2 titres to ChAdOx1-vaccinated HCs.

We observed variable non-response rates across disease groups depending on vaccine type and timepoint (Table S4). No HCs were seronegative after either two or three vaccine doses. LTRs had the highest rates of serological non-response with 18/52 (35%) and 52/179 (29%) having absent responses after two doses of ChAdOx1 and mRNA vaccines, respectively. Non-response rates were reduced at the post-V3 timepoint in all disease groups, with only 9/97 (9%) LTRs and 2/108 (2%) patients with cirrhosis not having a serological response to vaccine at the post-V3 timepoint.

Serological cross-reactivity of SARS-CoV-2 VoC

IgG binding to the spike protein of SARS-CoV-2 Omicron subvariants was assessed at post-V2 and post-V3 timepoints in selected samples from liver disease and HC cohorts (Fig. 2A). Within the combined cohort (liver disease and HCs) at both timepoints, serological titres to all Omicron subvariants (except for BF.7 and BQ.1) were significantly lower compared to WT SARS-CoV-2. The subvariants with the greatest decrease in IgG binding compared to WT were BA2.75.2 (median fold decrease: x8.93 post-V2 and x6.12 post-V3) and BA.4.6 (x4.46 post-V2 and x3.2 post-V3) which were both first identified in autumn 2022. Notably, the magnitude of reduction in IgG binding to Omicron subvariants relative to WT was lower following V3 compared to post-V2. The same trends were

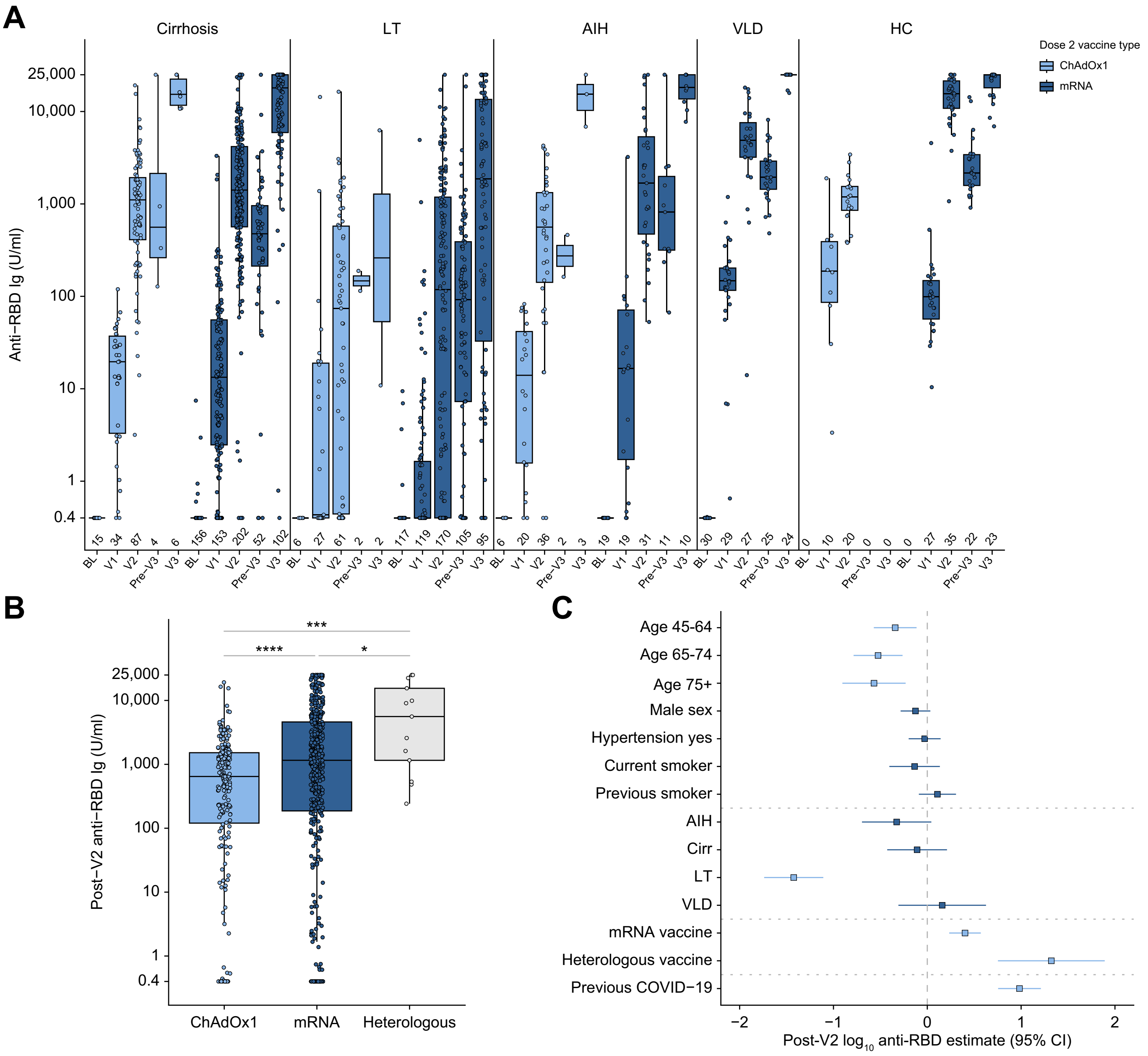

Fig. 1. COVID-19 vaccine-induced anti-SARS-CoV-2 RBD total Ig in SARS-CoV-2-naïve individuals. (A) Assessment of COVID-19 vaccine responses at baseline (BL), post first vaccine (V1), 28 days after second vaccine (V2), immediately prior to third vaccine (pre-V3) and 28 days after third vaccine (V3). All participants had an mRNA vaccine as their third dose, individuals vaccinated with a heterologous vaccine regimen were excluded. (B) Comparison of vaccination platforms at the post-V2 timepoint. (C) Forest plot depicting results from multivariable linear regression model of post-V2 log10 transformed anti-SARS-CoV-2 RBD Ig in all participants. (A,B) Boxes represent median and IQR, whiskers represent +/- 1.5x IQR. (C) Point represents odds ratio, whiskers 95% CI. (B) Mann-Whitney *U* test with Holm-Bonferroni adjustment. Dark blue indicates significantly predictive variables ($p < 0.05$). *$p < 0.05$, ***$p < 0.001$. AIH, autoimmune hepatitis; Cirr, cirrhosis; HCs, healthy controls; LT, liver transplant; RBD, receptor binding domain; VLD, vascular liver disease.

observed when IgG binding was split by liver disease aetiology (Fig. 2B and Fig. S3A). However, HCs had less of a decrease in IgG binding to Omicron subvariants relative to WT than seen in patients with liver disease. Serum from most participants inhibited ACE2 binding to WT RBD at post-V2 and post-V3 (Fig. 2C), whereas there was a significant decrease in inhibition across all Omicron subvariants relative to WT. Again, the decrease in ACE2 binding post-V3 was less pronounced compared to post-V2. There was also less of a decrease at the post-V2 and post-V3 timepoints in HCs compared to patients with liver disease (Figs 2D and S3B). The ratio of IgG binding to WT spike compared to VoC spike was significantly increased by a third vaccine in the liver disease group, but no change was observed between two and three vaccine responses in HCs (Fig. S3C). We observed significant positive correlations at post-V2 and post-V3 timepoints when comparing the Roche anti-RBD titre with VoC binding IgG, ACE2 inhibition, and with the ratio between WT to VoC binding IgG (Fig. S4A-C).

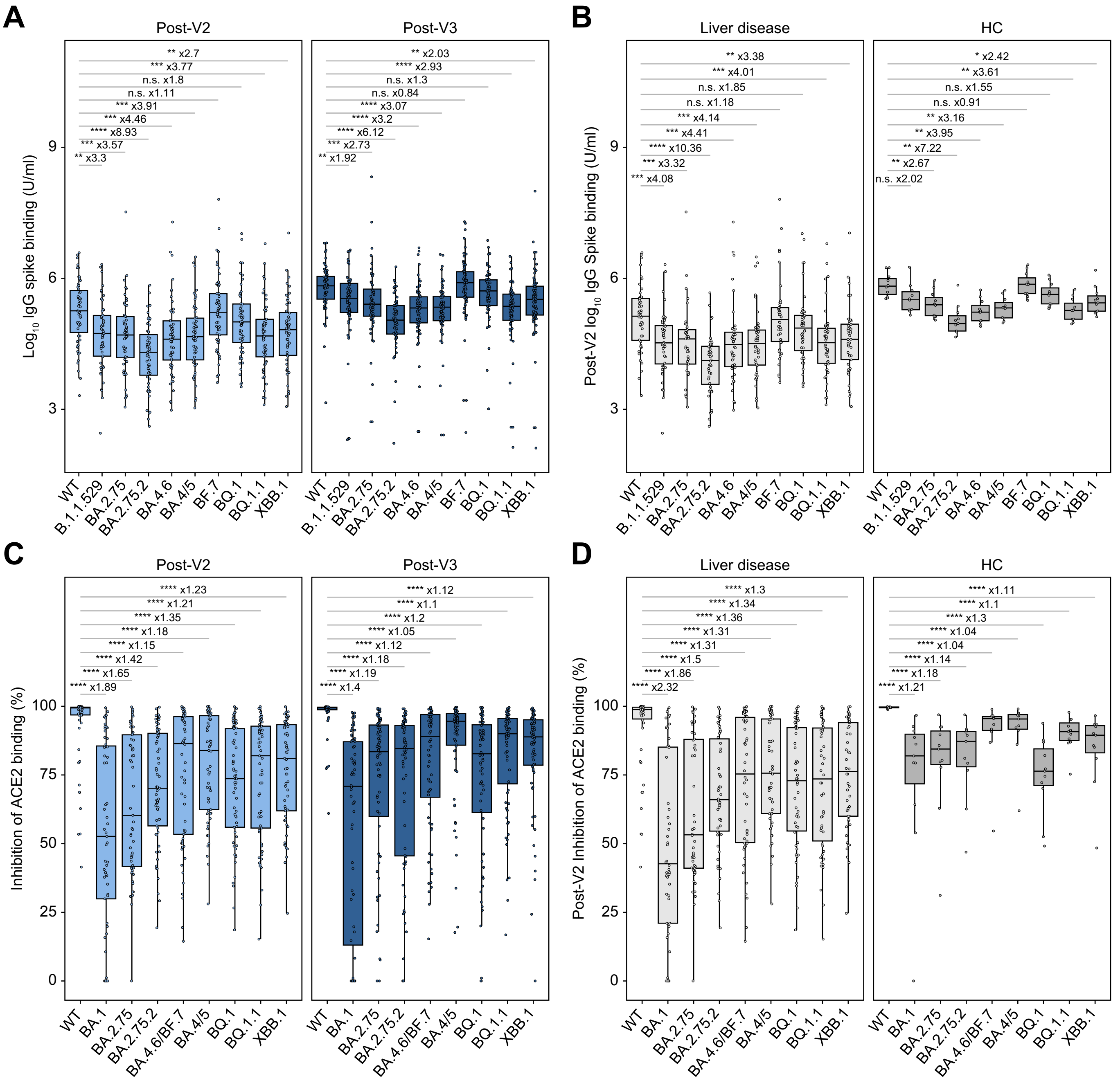

Fig. 2. COVID-19 vaccine-induced serological responses to Omicron subvariants. (A,B) IgG to subvariant spike protein and (C,D) inhibition of ACE2 binding to subvariant RBD in 68 individuals. (A,C) Includes all individuals split by timepoint and (B,D) are post-V2 responses separated by liver disease (inc. liver transplant) and HCs. Mann-Whitney *U* test with Holm-Bonferroni adjustment. Boxes represent median and IQR, whiskers represent +/- 1.5x IQR. Fold-change of median depicted. **p* < 0.05, ***p* < 0.01, ****p* < 0.001, *****p* <0.0001. ACE2, angiotensin-converting enzyme 2; HCs, healthy controls; RBD, receptor binding domain; WT, wild-type.

Liver transplant recipients

Antibody responses to two and three vaccine doses in LTRs are presented in Fig. 3A and are separated according to class of immunosuppression. This shows a downward trend in post-V2 anti-RBD titres associated with increasing intensity of immunosuppression, with significant reductions observed in patients on a calcineurin inhibitor (CNI) plus mycophenolate mofetil (MMF) *vs*. a CNI alone, and in patients on a CNI plus another immunosuppressant other than MMF, compared to CNI alone. LTRs on MMF additionally had significantly reduced responses compared to those on thiopurines (p = 0.011, Fig. S5A), and an increasing daily dose of MMF was significantly associated with decreased anti-RBD titres at the post-V2 timepoint (p = 0.031) (Fig. S5B). A third vaccine dose significantly improved antibody responses across all groups, except with mTOR inhibitor monotherapy where cohort numbers were small (Fig. 3A). LTRs had high rates of antibody non-response, with non-response rates at the post-V2 timepoint of 4/12 (33%)

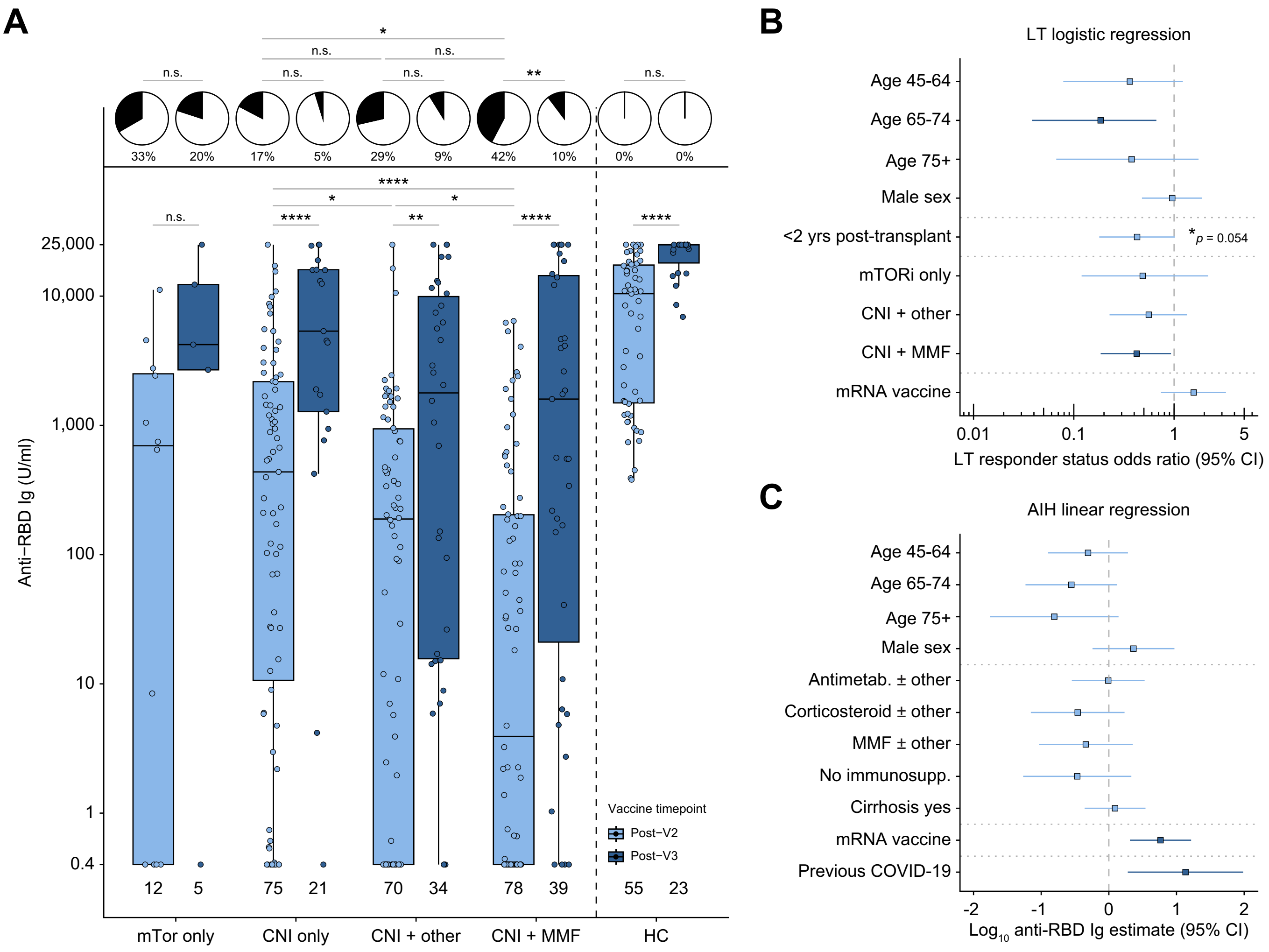

Fig. 3. Serological responses to COVID-19 vaccines in immunosuppressed patients with LT and AIH. (A) Magnitude of anti-SARS-CoV-2 RBD Ig in SARS-CoV-2 infection-naïve liver transplant recipients and HCs. Proportions of seropositive (white) and seronegative (black) (<0.8 U/ml) patients in each subgroup presented in pie charts. (B) Forest plot of multivariable logistic regression showing odds of seropositivity at the post-V2 timepoint within the liver transplant cohort and (C) results of multivariable linear regression of log10 anti-SARS-CoV-2 RBD Ig at post-v2 timepoint in autoimmune hepatitis cohort. (A) Boxes represent median and IQR, whiskers represent +/- 1.5x IQR. (B,C) Point represents odds ratio, whiskers 95% CI. (A) Mann-Whitney *U* test or Fisher's exact tests with Holm-Bonferroni adjustment. Dark blue indicates significantly associated variables ($p < 0.05$). n.s., non-significant, *$p < 0.05$, **$p < 0.01$, ***$p < 0.001$, ****$p < 0.0001$. AIH, autoimmune hepatitis; Antimetab, antimetabolite immunosuppression; CNI, calcineurin inhibitor; CNI + other, calcineurin inhibitor plus any immunosuppression other than MMF; HC, healthy control; LT, liver transplant; MMF, mycophenolate mofetil; mTORi, mTOR inhibitor; RBD, receptor binding domain.

in the mTOR inhibitor only group, 13/75 (17%) in the CNI only group, 20/70 (29%) in the CNI plus other immunosuppression group and 33/78 (42%) in the CNI plus MMF group. The rate of serological non-responsiveness was significantly higher in the CNI plus MMF group compared to the CNI alone group. A third vaccine dose led to improvement in rates of non-response across all subgroups of immunosuppression (mTOR inhibitor only 1/5 [20%]; CNI only 1/21 [5%]; CNI plus other immunosuppression 3/34 [9%]; CNI plus MMF 4/39 [10%]).

Among LTRs, both univariable and multivariable analyses showed that the factors significantly associated with reduced odds of seropositivity after V2 were age (65-74 age group) and CNI plus MMF (Fig. 3B, Table S5). LTRs with previous SARS-CoV-2 infection were removed from the logistic regression, as all of these patients had a detectable response after V2.

Patients with autoimmune hepatitis

Patients with AIH had higher post-V2 antibody responses than LTRs with both ChAdOx1 and mRNA platforms despite both groups being immunosuppressed and being of similar age (61 years [49–69] *vs.* 60 years [52–68]). Unlike LTRs, there were no significant differences in serological response between class and intensity of immunosuppression at post-V2, despite the fact some patients with AIH (6/8 with MMF dose data) were on high dose (2 g/day) MMF (Fig. S6A). There were also no differences in response when the AIH cohort was split by the presence or absence of cirrhosis (Fig. S6B). Univariable and multivariable analyses showed that the factors significantly associated with higher antibody responses after V2 were mRNA vaccination and previous COVID-19 (Fig. 3C, Table S6).

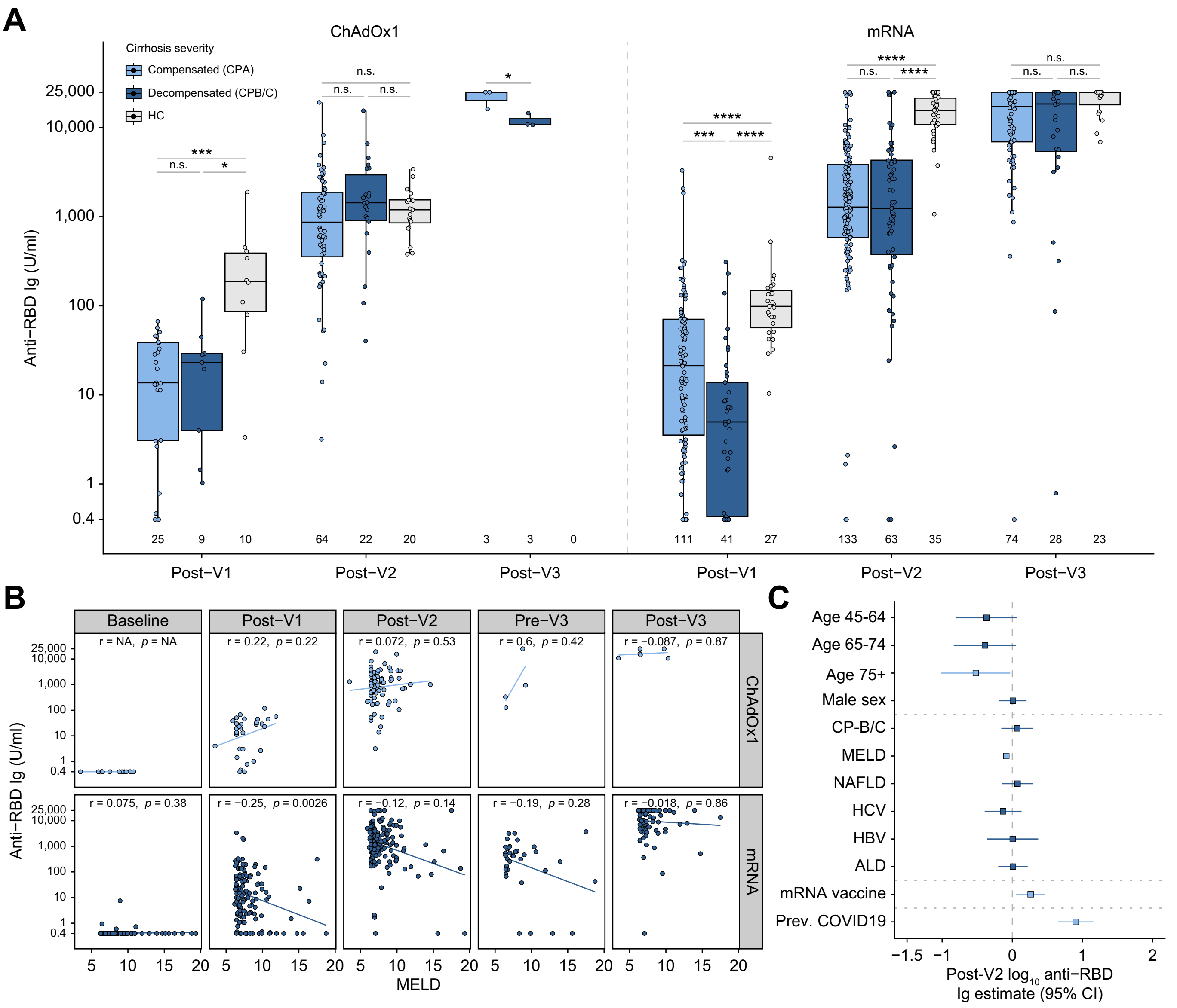

Fig. 4. Serological responses to COVID-19 vaccines in patients with cirrhosis. (A) Magnitude of vaccine responses in SARS-CoV-2 infection-naïve patients with cirrhosis and healthy controls. Vaccine type is for first two vaccine doses. (B) Spearman correlation of MELD score with anti-SARS-CoV-2 RBD Ig in same cirrhosis patients as A. (C) Forest plot depicting results of multivariable linear regression of log10 anti-SARS-CoV-2 RBD Ig at post-V2 timepoint in cirrhosis cohort. (A) Boxes represent median and IQR, whiskers represent +/- 1.5x IQR. (B) Line represents linear regression. (C) Point represents odds ratio, whiskers 95% CI. (A) Kruskal-Wallis with *post hoc* Dunn's test adjusted with Holm-Bonferroni method. (C) Dark blue indicates significantly associated variables (p <0.05). n.s., non-significant, **p < 0.01, ***p < 0.001, ****p < 0.0001. ALD, alcohol-related liver disease; CP, Child-Pugh class; HC, healthy control; NALFD, non-alcoholic fatty liver disease.

Patients with cirrhosis

Antibody responses in patients with cirrhosis at post-V1, post-V2, and post-V3 are presented in Fig. 4A and are separated according to vaccination type and CP class. This revealed a dynamic interaction between number of doses, severity of cirrhosis, and vaccine type. At post-V1 there were no differences in antibody titres between compensated (CP-A) and decompensated (CP–B/C) cirrhosis when vaccinated with ChAdOx1. However, CP-A did have higher antibodies than CP-B/C at post-V1 when vaccinated with mRNA. At post-V1, both CP-A and CP-B/C had lower antibody titres than HC with both vaccine types. At post-V2, there were no significant differences between CP-A and CP-B/C or between patients and HCs when vaccinated with ChAdOx1. Whereas at post-V2 for mRNA, HCs had higher titres than patients with cirrhosis irrespective of CP class. At post-V3, there were no significant differences between any groups. Broadly, this suggested an association between liver disease severity and reduced antibody responses when vaccinated with mRNA but not the ChAdOx1 platform, particularly early in the vaccination course. To explore the interaction between disease severity and vaccine type further we plotted the correlation between MELD score and anti-RBD titres (Fig. 4B). At post-V1, this again showed that increasing disease severity was associated with decreased anti-RBD titres when vaccinated with mRNA platform but not with ChAdOx1. This trend persisted after V2 and V3, although it was non-significant.

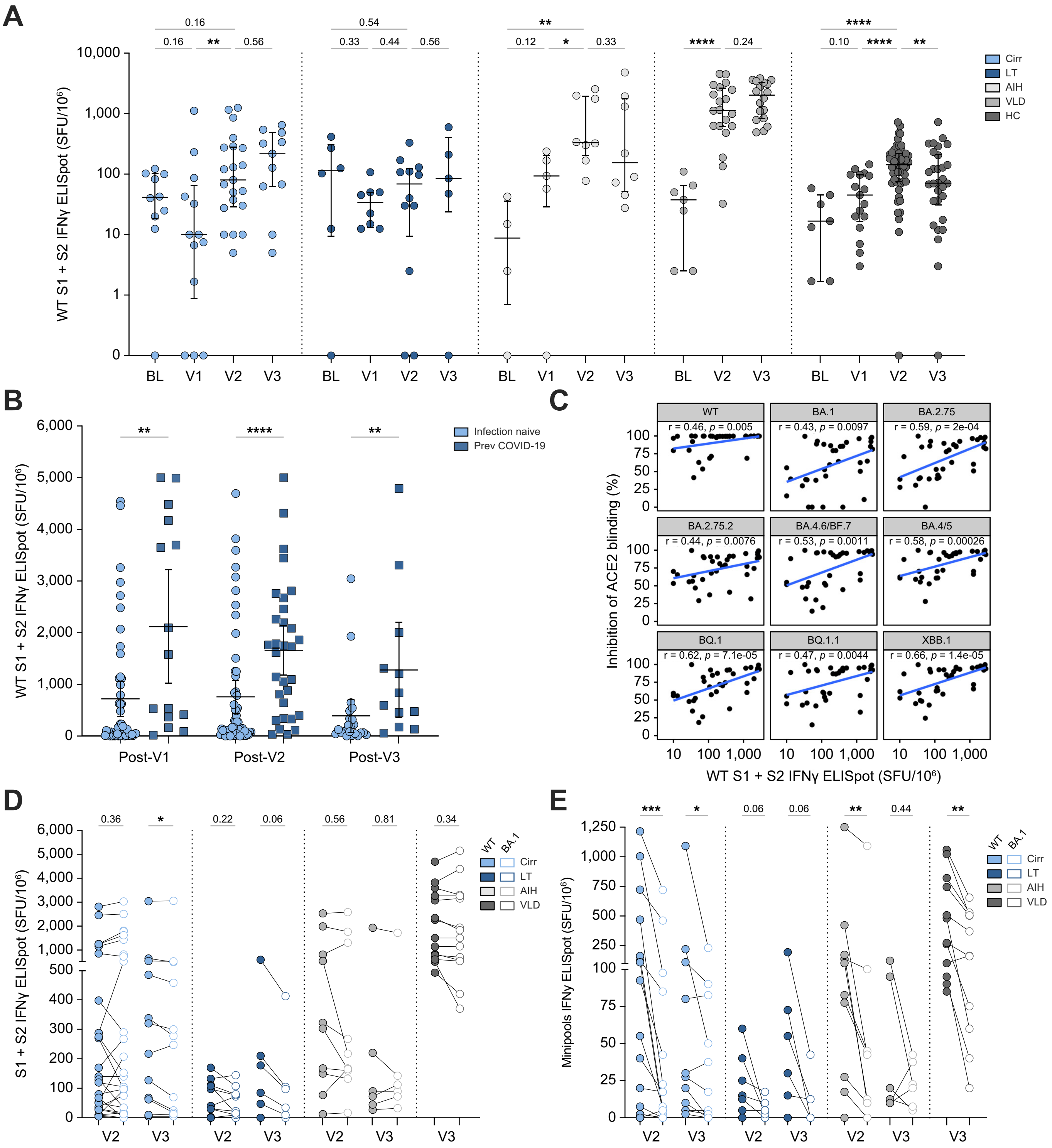

Fig. 5. IFNγ T-cell responses to COVID-19 vaccination. (A) Magnitude of IFNγ T-cell response to WT SARS-CoV-2 spike peptides across time in a subgroup of SARS-CoV-2-naïve individuals with cirrhosis (Cirr, n = 24), autoimmune hepatitis (AIH, n = 12), or vascular liver disease (VLD, n = 22), as well as in liver transplant recipients (LTRs, n = 12) and healthy controls (HCs, n = 28). Baseline data are from same individuals as later timepoints. (B) IFNγ T-cell responses in SARS-CoV-2-naïve (n = 68) and previously infected individuals (n = 31) across all disease groups. (C) Spearman correlation between magnitude of IFNγ T-cell responses to WT spike and percent inhibition of ACE2 binding to Omicron subvariant RBD by serum at post-V2 timepoint. (D,E) Magnitude of IFNγ T-cell responses to WT (filled circles) and Omicron BA.1 (open circles) peptides after two or three vaccines covering (D) whole spike and (E) minipools. (A,B) Line represents median and whiskers IQR. (C) Line represents linear regression. (A,B) Mann-Whitney *U* test. (D&E) Wilcoxon matched-paired signed rank test. n.s., non-significant, *$p < 0.05$,**$p < 0.01$,***$p < 0.001$,****$p < 0.0001$. AIH, autoimmune hepatitis; BL, baseline; RBD, receptor binding domain; SFU, spot-forming units; WT, wild-type.

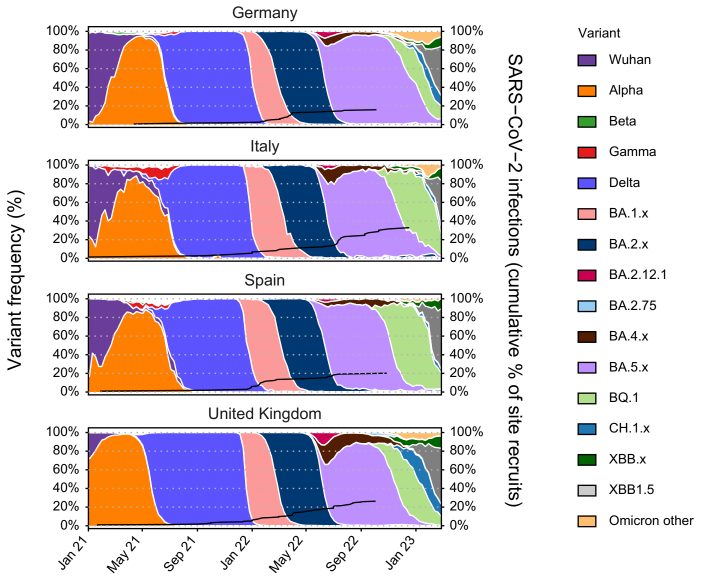

	Infection summary		COVID-19 severity	
	Uninfected (n = 320)	Infected (n = 122)	Mild-moderate COVID-19 (n = 117)	Severe/ hospitalised (n = 5)
**Age***	60 (51, 68)	59 (49, 66)	60 (49, 66)	58 (54, 68)
Unknown	0	1	1	0
**Sex (F)**	137 (43%)	54 (44%)	53 (45%)	1 (20%)
**Obesity**				
No	206 (64%)	72 (59%)	70 (60%)	2 (40%)
Yes	70 (22%)	37 (30%)	35 (30%)	2 (40%)
Unknown	44 (14%)	13 (11%)	12 (10%)	1 (20%)
**Disease type**				
AIH	29 (9.1%)	18 (15%)	18 (15%)	0 (0%)
Cirr	133 (42%)	46 (38%)	45 (38%)	1 (20%)
LT	130 (41%)	51 (42%)	47 (40%)	4 (80%)
VLD	28 (8.8%)	7 (5.7%)	7 (6.0%)	0 (0%)
**LT specific**				
<2yr post-Tx	26/130 (20%)	5/51 (9.8%)	4/47 (8.5%)	1/4 (25%)
Immunosup. MMF	47 (36%)	17 (14%)	15 (32%)	2 (40%)
**Child-Pugh score**				
Non-cirrhotic	146 (50%)	66 (57%)	69 (59%)	4 (80%)
A	83 (29%)	38 (33%)	37 (32%)	1 (20%)
B	43 (15%)	9 (8%)	9 (7.7%)	0 (0%)
C	19 (6.5%)	2 (2%)	2 (1.7%)	0 (0%)
**Infection timepoint**				
Post-V2	-	40 (33%)	38 (32%)	2 (40%)
Post-V3	-	47 (39%)	47 (40%)	0 (0%)
Post-V4	-	35 (29%)	32 (27%)	3 (60%)
Time-post vaccination (days)*	-	143 (76, 218)	143 (72, 231)	142 (122,165)
**Post-V2 anti-RBD serological status**				
High	196 (70%)	91 (76%)	88 (77%)	3 (60%)
Low	50 (18%)	15 (12%)	15 (13%)	0 (0%)
No	36 (13%)	14 (12%)	12 (10%)	2 (40%)
**Post-V2 anti-RBD Ig***	1,410 (208, 4,288)	1,020 (410, 3,718)	1,032 (432, 4,212)	565 (0, 1,234)
Unknown	16	2	2	0

Fig. 6. Breakthrough SARS-CoV-2 infection after two COVID-19 vaccines in liver disease cohort (n = 442). (A) Reported SARS-CoV-2 infection after second dose vaccine split across recruitment site countries. Frequency of SARS-CoV-2 and Omicron subvariants per country per week with cumulative proportion of infected individuals out of total individuals recruited at each site (black line). (B) Demographics and immunogenicity of cases with breakdown of severity (bottom panel). *Median and IQR. AIH, autoimmune hepatitis; Cirr, cirrhosis; LT, liver transplant; MMF, mycophenolate mofetil; VLD, vascular liver disease. (This figure appears in color on the web.)

Among patients with cirrhosis, both univariable and multivariable analyses showed that age over 75 years and increasing MELD score were associated with a lower antibody response, whereas mRNA platform and previous COVID-19 were associated with higher titres (Fig. 4C, Table S7).

Patients with vascular liver disease

All patients with VLD were vaccinated with an mRNA platform. The VLD cohort had lower post-V2 responses compared to mRNA-vaccinated HCs but equivalent titres post-V3. Patients with VLD had higher post-V2 antibody titres compared to mRNA-vaccinated LTRs (p < 0.0001), and patients with cirrhosis (p = 0.004) and AIH (p < 0.0001) (Tables S1 and S2).

T-cell responses

T-cell responses to WT SARS-CoV-2 across liver disease phenotypes

T-cell IFNγ responses to WT virus for a subset of infection-naïve participants from each disease group are presented in Fig. 5A and Fig. S7. The majority of patients across the entire cohort had positive T-cell responses (>26 SFU/10^6 PBMCs) after at least one dose of vaccination. Within the liver disease cohort all patients generated a positive T-cell response after V2 except for 3/10 (30%) LTRs, 4/24 (17%) with cirrhosis, and 1/12 (8%) with AIH. Despite significant heterogeneity, all groups except for LTRs had a significant increase in the magnitude of IFNγ responses after two or three vaccine doses (Fig. 5A). Within the total liver disease cohort, patients with previous COVID-19 had significantly higher IFNγ responses after V1, V2, and V3 (Fig. 5B). There were positive correlations between post-V2 T-cell responses to WT spike, anti-RBD binding antibodies to WT, and functional antibody responses to all variants (ACE2 binding inhibition) (Figs 5C and S4A).

T-cell responses to Omicron BA.1 SARS-CoV-2 across liver disease phenotypes

To determine the cross-reactivity of vaccine-induced cellular responses we additionally assessed IFNγ T-cell responses to peptides covering the Omicron (BA.1) spike protein (Fig. 5D). Compared to the WT antigen, T-cell responses to BA.1 spike were well preserved regardless of disease group at post-V2 and post-V3 timepoints. However, when only assessing responses to peptides that differed between WT and BA.1 spike (mutated peptide pools; "minipools") there were significant reductions in BA.1 peptide-specific reactivity compared to WT in all groups (Fig. 5E), indicating that T-cell responses specifically to mutated epitopes were reduced but overall responses were maintained.

Breakthrough SARS-CoV-2 infection after vaccination

SARS-CoV-2 infection status after COVID-19 vaccination was available for 442/722 (57%) of the entire liver disease cohort (Fig. 6A). Of these cases, 122/442 (28%) developed breakthrough infection after at least two vaccines; 40/122 (33%) after V2 and 47/122 (39%) after V3, and 35/122 (29%) after fourth vaccine (Fig. 6B). In those developing breakthrough infections, 117/122 (96%) were mild-moderate and 5/122 (4%) were severe. The majority (101/122, 83%) of breakthrough infections occurred after the emergence of Omicron as the dominant SARS-CoV-2 variant in each recruiting country. A breakthrough infection occurred in 51/181 (28%) LTRs, 46/179 (26%) patients with cirrhosis, 18/47 (38%) with AIH and 8/35 (23%) with VLD. Of those with severe breakthrough COVID-19, 4/5 (80%) were LTRs, two of whom were immunosuppressed with MMF and had absent anti-RBD responses after V2. Although sample size prevented robust statistical comparisons, the median post-V2 anti-RBD titre was numerically lower in those with severe COVID-19 compared to mild-moderate disease (565 U/ml [0-1,234] severe; 1,032 U/ml mild-moderate [432-4,212]) (Fig. S8). Two of five individuals with severe symptomatic breakthrough infection had PBMCs available at the post-V2 timepoint and had similar T-cell responses to WT spike (163 SFU/10^6 PBMCs and 65 SFU/10^6 PBMCs) as other LTRs (median 65 SFU/10^6 PBMCs) (Fig. 5A).

Discussion

In a large, international, prospective study we assess humoral and cellular immune responses to multiple COVID-19 vaccine platforms across a range of liver disease types and severities using standardised timepoints and laboratory assays. We report on functional antibody and cellular responses to novel viral variants including a number of the most up-to-date Omicron subtypes, and assess COVID-19 infection rates and disease severity.

Longitudinal serum sampling demonstrated a stepwise increase in the magnitude of anti-SARS-CoV-2 RBD antibodies following one, two and three vaccine doses, and following past SARS-CoV-2 infection. In our cohort, use of the mRNA platform was associated with greater antibody titres compared to ChAdOx1 which is in line with other datasets in healthy and immunosuppressed cohorts.[20,21] In addition, as observed in healthy populations,[22] heterologous first and second vaccination was associated with >5-fold increase in post-V2 antibody responses compared to homologous vaccine delivery, suggesting that this approach should also be considered in liver cohorts.

Our data identifies LTRs as a particularly vulnerable cohort, having the lowest post-V2 antibody titres compared to all other disease groups. Although the number of cases of severe breakthrough infection in our cohort was small, it is notable that 4/5 (80%) were LTRs, two of whom received MMF therapy and had absent anti-RBD responses after V2. Vaccine immunogenicity in LTRs was heavily influenced by intensity of immunosuppressive medication with MMF associated with low antibody titres and high rates of non-response and MMF dose negatively associated with anti-RBD titres. This supports evidence from other patient groups that short-term discontinuation or dose reductions of immunosuppressive therapy may help maximise antibody responses to vaccination.[23,24] Although other studies have reported suboptimal antibody responses in LTRs,[25,26] our cohort is notable for its size and geographic diversity. It also allows for direct comparisons with immunosuppressed patients with AIH who have more robust antibody responses despite being of similar age. This is most likely accounted for by the predominant use of thiopurines and corticosteroids, and the absence of MMF plus CNI dual therapy in patients with AIH. Reassuringly, irrespective of immunosuppressive status, antibody titres and rates of seroconversion were universally improved in all groups following the third vaccination, though some LTRs remained non-responsive to vaccination.

In parallel with increasing serological titres to WT SARS-CoV-2, the cross-reactivity of vaccine-induced antibodies to VoC also improved across the liver disease cohort between post-V2 and post-V3 timepoints. This suggests that increasing the magnitude of serological response through a third vaccination maximises the likelihood that a proportion of antibodies will cross-react with VoC even in individuals who may have reduced capacity to produce high-affinity class-switched or somatically hypermutated antibodies.[27] Despite improvements in antibody function with repeat vaccination, there was still a significant decrease in IgG binding and ACE-2 binding inhibition to nearly all Omicron subvariants in patients with liver disease after V2 and V3 relative to WT virus. This immune escape may partly account for the clear stepwise increments in breakthrough infection rates observed in our cohort.

Early in the vaccination course, severity of cirrhosis (indicated by CP class and MELD score) was associated with lower antibody responses to mRNA but not ChAdOx1. Although this effect was ultimately overcome by repeated vaccine doses it does point towards differential immunological mechanisms governing antibody response according to vaccine type. mRNA and adenoviral vector platforms are thought to induce antibody production through varying biological pathways which may be differentially impacted by cirrhosis-associated immune dysfunction.[28,29] Further work is required to decipher the complex interplay between cirrhosis-associated immune dysfunction and immune responses to COVID-19 vaccination. Nonetheless, it is important to note that mRNA vaccines still induce higher antibody titres than ChAdOx1 at the post-V2 timepoint.

Despite heterogeneity in the magnitude of T-cell responses across HCs (as observed elsewhere[18]) and disease cohorts, the majority of assessed individuals (88%) mounted an IFNγ response to WT SARS-CoV-2 spike antigens after a single vaccine dose which was preserved after V2 and V3. Notably, nearly all infection-naïve participants had low-level T-cell responses detected at baseline, likely as a consequence of cross-reactivity with seasonal human coronaviruses, or possible undetected previous COVID-19 due to waning of post-infection nucleocapsid antibodies.[30–32] Patients with VLD had particularly robust IFNγ T-cell responses compared to other disease cohorts, which is possibly related to the absence of immunosuppressive medications, a possible unknown biological mechanism, preserved liver function, and use of mRNA-1273 vaccination which has previously been shown in healthy individuals to be associated with higher CD4+ and CD8+ SARS-CoV-2-specific T-cell responses.[21] Overall, T-cell responses in individuals with liver diseases were also well maintained against the Omicron BA.1 variant, however reduced responses to BA.1 minipools suggest that responses to specific mutated epitopes may be lost.[33] The cellular immune response to vaccination has emerged as a major determinant of individual risk of developing severe COVID-19, including in immunocompromised individuals.[13,14] This may help explain why the majority of breakthrough infections reported in the liver disease cohort were mild-moderate with only 5/122 (4%) total infections reported as severe. However, post-V2 T-cell responses were detectable in the two participants with severe breakthrough COVID-19 and available PBMCs in this cohort, and other factors including potentially reduced virulence of later SARS-CoV-2 variants may additionally impact this.[34]

There are some limitations to our study. Firstly, despite recruiting from four different countries with diverse immunisation regimens, certain vaccine platforms in particular groups are lacking, including mRNA-1273-vaccinated HCs, third dose of ChAdOx1 in HCs, and ChAdOx1-or BNT162b2-vaccinated patients with VLD. Although it is tempting to extrapolate the immunological principles identified across three vaccines, the precise immune changes after multiple subsequent vaccine doses remain to be determined. Another constraint of our dataset is that the HC cohort is comprised of healthcare workers who are significantly younger with fewer comorbidities than the liver disease population. However, we have performed a multivariable analysis of the entire cohort which accounts for age in order to identify cofactors and disease groups associated with vaccine response. The breakthrough COVID-19 data must also be interpreted with caution as asymptomatic infection may not have been identified and it remains impossible to fully account for important confounding variables such as local SARS-CoV-2 prevalence, viral load exposure, further vaccine doses, and individual patient behaviours including shielding measures. Furthermore, we were unable to systematically collect accurate data on the use of antiviral medications and recombinant antibodies due to incomplete documentation in electronic hospital records and geographic variability in access to these agents. As a result, we have opted to remain descriptive with this domain of the study and have not performed statistical analyses. Lastly, due to sampling limitations, IFNγ T-cell assay results were not available in all participants with severe breakthrough infection.

In summary, we demonstrate that the three most widely available vaccine platforms are immunogenic and appear to protect against severe COVID-19 in a diverse group of patients with a variety of underlying liver conditions. Even patients with advanced cirrhosis mount robust immune responses after two and three vaccine doses irrespective of vaccine type. This will provide reassurance to patients with chronic liver disease who were previously deemed at high risk of severe COVID-19 and death during the pre-vaccination era. In addition, our data will be encouraging in the event of future unforeseen viral infections which may also require rapid vaccine development and delivery to patients with liver disease. However, we show that LTRs mount lower antibody and T-cell responses, related to intensity of immunosuppression and the use of MMF, with most cases of severe COVID-19 occurring in this patient group. We recommend that LTRs should be vigilantly monitored for the development of severe COVID-19 if infected, and prioritised for repeated vaccination, prophylactic antiviral agents, and enrolment into trials exploring the role of immunosuppressive dose modification and alternative vaccine strategies.

Affiliations

[1]Peter Medawar Building for Pathogen Research, Nuffield Department of Clinical Medicine, University of Oxford, Oxford, UK; [2]Liver Unit, Hospital Clínic, Institut de Investigacions Biomèdiques August Pi i Sunyer (IDIBAPS), University of Barcelona, Barcelona, Spain; [3]CIBERehd (Centro de Investigación Biomédica en Red Enfermedades Hepáticas y Digestivas), Spain; [4]German Center for Infection Research (DZIF), Partner Site Hamburg-Lübeck-Borstel-Riems, Germany; [5]Department of Internal Medicine, University Medical Center Hamburg-Eppendorf, Hamburg, Germany; [6]Centre for Statistics in Medicine, University of Oxford, Oxford, UK;

[7]Translational Gastroenterology Unit, Nuffield Department of Medicine, University of Oxford, Oxford, UK; [8]Health Care Provider of the European Reference Network on Rare Liver Disorders (ERN-Liver), Germany; [9]National Institute for Health Research Birmingham Biomedical Research Centre, Centre for Liver and Gastrointestinal Research, Institute of Immunology and Immunotherapy, University of Birmingham, Germany; [10]Liver Unit, University Hospitals Birmingham NHS Foundation Trust, Queen Elizabeth Hospital, Birmingham, UK; [11]Division of Digestive Diseases, Department of Metabolism, Digestion and Reproduction, Faculty of Medicine, Imperial College London, London, UK; [12]Department of Hepatology, St Mary's Hospital, Imperial College Healthcare NHS Trust, London, UK; [13]Wellcome Centre for Human Genetics, University of Oxford, Oxford, UK; [14]University of Padova, Department of Surgery, Oncology and Gastroenterology DISCOG, Italy; [15]The Oxford NIHR Biomedical Research Centre, Oxford University Hospital NHS Trust, Oxford, UK; [16]Mahidol Oxford Tropical Medicine Research Unit, University of Mahidol, Bangkok, Thailand; [17]Laboratory of Synthetic Immunology, Department of Surgery, Oncology and Gastroenterology, University of Padova, Padova, Italy; [18]Veneto Institute of Oncology IOV-IRCCS, Padova, Italy; [19]Division of Gastroenterology and Hepatology, Foundation IRCCS Ca' Granda Ospedale Maggiore Policlinico, Milan, Italy; [20]CRC "A. M. and A. Migliavacca" Center for Liver Disease, Department of Pathophysiology and Transplantation, University of Milan, Milan, Italy; [21]Institute of Medical Microbiology, Virology and Hygiene, University Medical Center Hamburg-Eppendorf, Hamburg, Germany; [22]Oxford Centre for Diabetes, Endocrinology and Metabolism (OCDEM), NIHR Oxford Biomedical Research Centre, Churchill Hospital, University of Oxford, Oxford, UK; [23]Oxford Liver Unit, Oxford University Hospitals NHS Foundation Trust, John Radcliffe Hospital, Oxford, UK

Abbreviations

AIH, autoimmune hepatitis; CNI, calcineurin inhibitor; ChAdOx1, ChAdOx1 nCoV-19; CP, Child-Pugh; HC, healthy control; MELD, model for end-stage liver disease; MMF, mycophenolate mofetil; RBD, receptor binding domain; LTRs, liver transplant recipients; VoC, variant of concern; VLD, vascular liver disease; WT, wild-type.

Financial support

The COVID-Hep vaccine network is supported by a registry grant from the European Association for the Study of the Liver (EASL). The UK OCTAVE study is funded by a grant from UK Research and Innovation (UKRI) administered by the Medical Research Council (reference: MC_PC_20031). PITCH is funded by the UK Department of Health and Social Care by UKRI as part of "Investigation of proven vaccine breakthrough by SARS-CoV-2 variants in established UK healthcare worker cohorts: SIREN consortium & PITCH Plus Pathway (reference: MR/W02067X/1), with contributions from UKRI/NIHR through the UK Coronavirus Immunology Consortium (UK–CIC), the Huo Family Foundation and the NIHR UKRIDHSC COVID-19 Rapid Response Rolling Call (reference: COV19-RECPLAS). SMM is supported by the Medical Research Council. T.M. is supported via a Wellcome Trust Clinical Research Training Fellowship (reference: 102176/B/13/Z). E.B. is supported by the Oxford NIHR Biomedical Research Centre and is an NIHR Senior Investigator. P.J.T receives institutional salary support from the National Institute for Health Research (NIHR) Birmingham Biomedical Research Centre (BRC). BHM is the recipient of an NIHR Academic Clinical Lectureship (CL-2019-21-002). A.W.L, J.S.z.W., M.L, receive financial support from the German Center for Infection Research (DZIF). Part of the work of this study (P.G.) has been funded by a grant of the Instituto de Salud Carlos III-ISCIII, grant number: PI020/00579. The Division of Digestive Diseases at Imperial College London receives financial and infrastructure support from the NIHR Imperial Biomedical Research Centre (BRC) based at Imperial College Healthcare NHS Trust and Imperial College London. The views expressed in this article are those of the authors and not necessarily those of the NHS, the NIHR, or the Department of Health.

Conflicts of interest

E.B. and P.K. have received consultancy fees from AstraZeneca. E.B has received consultancy fees from Vaccitech. P.L. is on the advisory board for BMS, Roche, Gilead Sciences, GSK, AbbVie, MSD, Arrowhead, ALNYLAM, Janssen, SBRING Bank, MYR, Eiger, Antios, ALIGOS, VI. M.I. is on the advisory board or has been a speaker for Bureau for Bayer, Gilead Sciences, BMS, Janssen, Ipsen, MSD, BTG-Boston Scientific, AbbVie, Guerbet, EISAI, Roche, AstraZeneca. P.J.T has received grant support from the Wellcome Trust, the Medical Research Foundation, LifeArc, Innovate UK, GSK, Guts UK, PSC Support, Intercept/Advanz Pharma, Dr. Falk Pharma, Gilead sciences, and Bristol Myers Squibb, speaker fees from Intercept and Dr Falk, and advisory board/consultancy fees from Albireo, Cymabay, Pliant Pharma, IPSEN, Intercept, Dr. Falk and GSK. MCL has received advisory fees from Intercept/AdvanzPharma, IPSEN, GSK and lecture fees from Intercept/AdvanzPharma. All other authors declare no conflicts of interest.

Please refer to the accompanying ICMJE disclosure forms for further details.

Authors' contributions

TM and EB are Chief Investigators of the EASL supported COVID-Hep vaccine network. SJD and PK are the Chief Investigators of the PITCH Study. EB, SJD, are members of the OCTAVE Trial Management Group. TM and EB wrote the grant proposal for EASL registry funding. TM, EB, FPR, JSZW, MI, ML, EP wrote the EASL network study protocol. SMM, GM, SI, NP performed T-cell laboratory assays. ML and JSZW performed antibody titre assays. MC, TT, and SH performed variant of concern antibody assays. TM, EB, MW, GS, PD, ZL, SD, GM, ML, PT, KB, BM, PM, EP, MI, MS, JSZW, PG, VHG, JGP, VP were all involved in sample and/or clinical data collection. SMM, JC, and TM performed the statistical analysis. SMM and TM wrote the initial manuscript. All named authors contributed to the interpretation of the analyses and the writing of the paper. The corresponding authors had full access to all the data in the study and had final responsibility for the decision to submit for publication.

Data availability statement

Data may be made available upon reasonable request to corresponding author.

Acknowledgements

We gratefully acknowledge all GISAID EpiCoV data contributors, i.e., the authors and their originating laboratories responsible for obtaining the specimens, and their submitting laboratories for generating the genetic sequence and metadata and sharing via the GISAID Initiative. We acknowledge with gratitude the input of Elspeth Insch and Richard Beesley who gave valuable feedback on this manuscript as patient representatives. We thank all patients and healthy volunteers for their participation in the study.

Supplementary data

Supplementary data to this article can be found online at .https://doi.org/10.1016/j.jhep.2023.10.009

References

Author names in bold designate shared co-first authorship

[1] **Nyberg T**, **Ferguson NM**, Nash SG, Webster HH, Flaxman S, Andrews N, et al. Comparative analysis of the risks of hospitalisation and death associated with SARS-CoV-2 omicron (B. 1.1. 529) and delta (B. 1.617. 2) variants in England: a cohort study. The Lancet 2022;399:1303–1312.

[2] Moghadas SM, Vilches TN, Zhang K, Wells CR, Shoukat A, Singer BH, et al. The impact of vaccination on coronavirus disease 2019 (COVID-19) outbreaks in the United States. Clin Infect Dis 2021;73:2257–2264.

[3] Marjot T, Webb GJ, Barritt AS, Ginès P, Lohse AW, Moon AM, et al. SARS-CoV-2 vaccination in patients with liver disease: responding to the next big question. Lancet Gastroenterol Hepatol 2021;6:156–158.

[4] **Marjot T**, **Moon AM**, Cook JA, Abd-Elsalam S, Aloman C, Armstrong MJ, et al. Outcomes following SARS-CoV-2 infection in patients with chronic liver disease: an international registry study. J Hepatol 2021;74:567–577.

[5] **Webb GJ**, **Marjot T**, Cook JA, Aloman C, Armstrong MJ, Brenner EJ, et al. Outcomes following SARS-CoV-2 infection in liver transplant recipients: an international registry study. Lancet Gastroenterol Hepatol 2020;5:1008–1016.

[6] Baiges A, Cerda E, Amicone C, Téllez L, Alvarado-Tapias E, Puente A, et al. Impact of SARS-CoV-2 pandemic on vascular liver diseases. Clin Gastroenterol Hepatol 2022;20:1525–1533. e1525.

[7] Iavarone M, D'Ambrosio R, Soria A, Triolo M, Pugliese N, Del Poggio P, et al. High rates of 30-day mortality in patients with cirrhosis and COVID-19. J Hepatol 2020;73:1063–1071.

[8] John BV, Ferreira RD, Doshi A, Kaplan DE, Taddei TH, Spector SA, et al. Third dose of COVID-19 mRNA vaccine appears to overcome vaccine hyporesponsiveness in patients with cirrhosis. J Hepatol 2022;77:1349–1358.

[9] Marjot T, Eberhardt CS, Boettler T, Belli LS, Berenguer M, Buti M, et al. Impact of COVID-19 on the liver and on the care of patients with chronic liver disease, hepatobiliary cancer, and liver transplantation: an updated EASL position paper. J Hepatol 2022;77:1161–1197.

[10] Luo D, Chen X, Du J, Mei B, Wang A, Kuang F, et al. Immunogenicity of COVID-19 vaccines in chronic liver disease patients and liver transplant recipients: a systematic review and meta-analysis. Liver Int 2023;43:34–48.
[11] Toniutto P, Falleti E, Cmet S, Cussigh A, Veneto L, Bitetto D, et al. Past COVID-19 and immunosuppressive regimens affect the long-term response to anti-SARS-CoV-2 vaccination in liver transplant recipients. J Hepatol 2022;77:152–162.
[12] **Luxenburger H**, **Reeg DB**, **Lang-Meli J**, Reinscheid M, Eisner M, Bettinger D, et al. Boosting compromised SARS-CoV-2-specific immunity with mRNA vaccination in liver transplant recipients. J Hepatol 2023;78:1017–1027.
[13] Scurr MJ, Lippiatt G, Capitani L, Bentley K, Lauder SN, Smart K, et al. Magnitude of venous or capillary blood-derived SARS-CoV-2-specific T cell response determines COVID-19 immunity. Nat Commun 2022;13:5422.
[14] **Barnes E**, **Goodyear CS**, **Willicombe M**, **Gaskell C**, Siebert S, I de Silva T, et al. SARS-CoV-2-specific immune responses and clinical outcomes after COVID-19 vaccination in patients with immune-suppressive disease. Nat Med 2023;29:1760–1774.
[15] Duengelhoef P, Hartl J, Rüther D, Steinmann S, Brehm TT, Weltzsch JP, et al. SARS-CoV-2 vaccination response in patients with autoimmune hepatitis and autoimmune cholestatic liver disease. United Eur Gastroenterol J 2022;10:319–329.
[16] Harberts A, Schaub GM, Ruether DF, Duengelhoef PM, Brehm TT, Karsten H, et al. Humoral and cellular immune response after third and fourth SARS-CoV-2 mRNA vaccination in liver transplant recipients. Clin Gastroenterol Hepatol 2022;20:2558–2566.e2555.
[17] Clerical changes for implementation of adding serum sodium to the MELD score. Organ Procurement and Transplantation Network; 2015.
[18] Payne RP, Longet S, Austin JA, Skelly DT, Dejnirattisai W, Adele S, et al. Immunogenicity of standard and extended dosing intervals of BNT162b2 mRNA vaccine. Cell 2021;184:5699–5714.e5611.
[19] World Health Organization. Living guidance for clinical management of COVID-19: living guidance, 23 November 2021. World Health Organization; 2021.
[20] McDonald I, Murray SM, Reynolds CJ, Altmann DM, Boyton RJ. Comparative systematic review and meta-analysis of reactogenicity, immunogenicity and efficacy of vaccines against SARS-CoV-2. npj Vaccin 2021;6:74.
[21] Klemis V, Schmidt T, Schub D, Mihm J, Marx S, Abu-Omar A, et al. Comparative immunogenicity and reactogenicity of heterologous ChAdOx1-nCoV-19-priming and BNT162b2 or mRNA-1273-boosting with homologous COVID-19 vaccine regimens. Nat Commun 2022;13:4710.
[22] **Stuart ASV**, **Shaw RH**, **Liu X**, Greenland M, Aley PK, Andrews NJ, et al. Immunogenicity, safety, and reactogenicity of heterologous COVID-19 primary vaccination incorporating mRNA, viral-vector, and protein-adjuvant vaccines in the UK (Com-COV2): a single-blind, randomised, phase 2, non-inferiority trial. The Lancet 2022;399:36–49.
[23] Abhishek A, Boyton RJ, Peckham N, McKnight Á, Coates LC, Bluett J, et al. Effect of a 2-week interruption in methotrexate treatment versus continued treatment on COVID-19 booster vaccine immunity in adults with inflammatory conditions (VROOM study): a randomised, open label, superiority trial. Lancet Respir Med 2022;10:840–850.
[24] Kantauskaite M, Müller L, Hillebrandt J, Lamberti J, Fischer S, Kolb T, et al. Immune response to third SARS-CoV-2 vaccination in seronegative kidney transplant recipients: possible improvement by mycophenolate mofetil reduction. Clin Transplant 2022;36:e14790.
[25] Thuluvath PJ, Robarts P, Chauhan M. Analysis of antibody responses after COVID-19 vaccination in liver transplant recipients and those with chronic liver diseases. J Hepatol 2021;75:1434–1439.
[26] Ruether DF, Schaub GM, Duengelhoef PM, Haag F, Brehm TT, Fathi A, et al. SARS-CoV2-specific humoral and T-cell immune response after second vaccination in liver cirrhosis and transplant patients. Clin Gastroenterol Hepatol 2022;20:162–172. e169.
[27] Eickenberg S, Mickholz E, Jung E, Nofer JR, Pavenstadt HJ, Jacobi AM. Mycophenolic acid counteracts B cell proliferation and plasmablast formation in patients with systemic lupus erythematosus. Arthritis Res Ther 2012;14:R110.
[28] Provine NM, Klenerman P. Adenovirus vector and mRNA vaccines: mechanisms regulating their immunogenicity. Eur J Immunol 2022;53:2250022.
[29] Albillos A, Martin-Mateos R, Van der Merwe S, Wiest R, Jalan R, Álvarez-Mon M. Cirrhosis-associated immune dysfunction. Nat Rev Gastroenterol Hepatol 2022;19:112–134.
[30] Murray SM, Ansari AM, Frater J, Klenerman P, Dunachie S, Barnes E, et al. The impact of pre-existing cross-reactive immunity on SARS-CoV-2 infection and vaccine responses. Nat Rev Immunol 2023;23:304–316.
[31] Iavarone M, Tosetti G, Facchetti F, Topa M, Er JM, Hang SK, et al. Spike-specific humoral and cellular immune responses after COVID-19 mRNA vaccination in patients with cirrhosis: a prospective single center study. Dig Liver Dis 2023;55:160–168.
[32] **Tomic A**, **Skelly DT**, **Ogbe A**, **O'Connor D**, Pace M, Adland E, et al. Divergent trajectories of antiviral memory after SARS-CoV-2 infection. Nat Commun 2022;13:1251.
[33] **Moore SC**, **Kronsteiner B**, **Longet S**, Adele S, Deeks AS, Liu C, et al. Evolution of long-term vaccine-induced and hybrid immunity in healthcare workers after different COVID-19 vaccine regimens. Med 2023;4:191–215.e199.
[34] **Carabelli AM**, **Peacock TP**, Thorne LG, Harvey WT, Hughes J, de Silva TI, et al. SARS-CoV-2 variant biology: immune escape, transmission and fitness. Nat Rev Microbiol 2023;21:162–177.

Journal of Hepatology, Volume *80*

Supplemental information

Immune responses and clinical outcomes after COVID-19 vaccination in patients with liver disease and liver transplant recipients

Sam M. Murray, Elisa Pose, Melanie Wittner, Maria-Carlota Londoño, Golda Schaub, Jonathan Cook, Stavros Dimitriadis, Georgina Meacham, Sophie Irwin, Zixiang Lim, Paul Duengelhoef, Martina Sterneck, Ansgar W. Lohse, Valeria Perez, Palak Trivedi, Khush Bhandal, Benjamin H. Mullish, Pinelopi Manousou, Nicholas M. Provine, Emma Avitabile, Miles Carroll, Tom Tipton, Saoirse Healy, Patrizia Burra, Paul Klenerman, Susanna Dunachie, Barbara Kronsteiner, Agnieszka Katarzyna Maciola, Giulia Pasqual, Virginia Hernandez-Gea, Juan Carlos Garcia-Pagan, Pietro Lampertico, Massimo Iavarone, Pere Gines, Marc Lütgehetmann, Julian Schulze zur Wiesch, Francesco Paolo Russo, Eleanor Barnes, Thomas Marjot, and on behalf of the OCTAVE Collaborative Group, PITCH study, and the EASL supported COVID-Hep vaccine network

Immune responses and clinical outcomes after COVID-19 vaccination in patients with liver disease and in liver transplant recipients

Sam M. Murray, Elisa Pose, Melanie Wittner, Maria-Carlota Londoño, Golda Schaub, Jonathan Cook, Stavros Dimitriadis, Georgina Meacham, Sophie Irwin, Zixiang Lim, Paul Duengelhoef, Martina Sterneck, Ansgar W. Lohse, Valeria Perez, Palak Trivedi, Khush Bhandal, Ben Mullish, Pinelopi Manousou, Nicholas M. Provine, Emma Avitabile, Miles Carroll, Tom Tipton, Saoirse Healy, Patrizia Burra, Paul Klenerman, Susanna Dunachie, Barbara Kronsteiner, Agnieszka Katarzyna Maciola, Giulia Pasqual, Virginia Hernandez-Gea, Juan Carlos Garcia-Pagan, Pietro Lampertico, Massimo Iavarone, Pere Gines, Marc Lütgehetmann, Julian Schulze zur Wiesch, Francesco Paolo Russo, Eleanor Barnes, Thomas Marjot on behalf of the OCTAVE Collaborative Group, PITCH study, and the EASL supported COVID-Hep vaccine network

Table of contents

OCTAVE Collaborative group 4
EASL supported COVID-Hep vaccine network 6
PITCH Consortium 7
Supplementary methods 8
Fig. S1 10
Fig. S2 11
Fig. S3 12
Fig. S4 13
Fig. S5 14
Fig. S6 15

Fig. S7 16
Fig. S8 17
Table S1 18
Table S2 19
Table S3 20
Table S4 21
Table S5 22
Table S6 23
Table S7 24
Supplementary reference 25

OCTAVE Collaborative group

Gary Middleton	Cancer Centre, University Hospitals Birmingham, NHS Foundation Trust, Birmingham B15 2WB, UK
Charlotte Gaskell	
Daniel Rea	
Sarah Pirrie	
Sarah J Bowden	
Ann Pope	
Ana Hughes	
Molly Harrison	
Amanda Kirkham	
Lucinda Middleton	
Faye Lowe	
Sophia Magwaro	
Pamela Kearns	National Institute for Health Research Birmingham Biomedical Research Centre, Institute of Cancer and Genomic Sciences, University of Birmingham, Birmingham B15 2TT UK.
	Cancer Research UK Clinical Trials Unit (CRCTU), University of Birmingham, Edgbaston, Birmingham. B15 2TT, UK.
Sean H Lim	Centre for Cancer Immunology, University of Southampton, Southampton, SO16 6YD UK.
Michelle Willicombe	Centre for Inflammatory Disease, Department of Immunology and Inflammation, Imperial College London, Hammersmith Campus, Du Cane Road, London W12 0NN UK.
Maria Prendecki	
Candice Clarke	
Paige Mortimer	
Stacey McIntyre	
David Thomas	
Alex Richter	Clinical Immunology Service, University of Birmingham, Edgbaston, Birmingham. B15 2TT, UK.
Sally Al-Taei	
Carl S Goodyear	College of Medical, Veterinary & Life Sciences; University of Glasgow, Glasgow; G12 8QQ, UK.
Stefan Siebert	
Neil Basu	
Ashley Gilmour	
Iain B McInnes	
Andrew Tong	Department of Haematology, Cambridge University Hospitals NHS Foundation Trust, Cambridge, CB2 OQQ, UK
Kieran Woolcock	
Faisal Basheer	
Charles Crawley	
Ram Malladi	
Andrew King	
Sophie Lockey	
Ben Uttenthal	
John A Snowden	Department of Haematology, Sheffield Teaching Hospitals NHS Foundation Trust, Royal Hallamshire Hospital, Sheffield. S10 2JF, UK.
Rachael Selby	
Kim Orchard	Department of Haematology, University Hospital Southampton NHS Foundation Trust, Southampton, S016 6YD UK.
Thushan I de Silva	Department of Infection, Immunity and Cardiovascular Disease, The Medical School, The University of Sheffield, Sheffield. S10 2RX, UK.
Naomi Meardon	
Sam Hansford	
Gurjinder Sandhar	
Peter Kelleher	Department of Infectious Diseases, Imperial College London, School of Medicine Chelsea and Westminster Hospital, London SW10 9NH UK
Murali Kesavan	Department of Oncology, Cancer and Haematology Centre, Churchill Hospital, Old Road, Headington, Oxford, OX3 7LE, UK
Celia Moore	Division of Digestive Diseases, Department of Metabolism, Digestion and Reproduction, Faculty of Medicine, Imperial College London, London, W2 1NY, UK
Pinelopi Manousou	
Gareth Hahn	
Benjamin Mullish	
Maria Atta	Haematology Department, Hammermith Hospital, London, W12 0HS UK
Sarah Gleeson	Imperial College Healthcare NHS Trust, Hammersmith Hospital, London, W12 OHS, UK
Liz Lightstone	
Paul Martin	
Stephen McAdoo	
Tina Thomson	
Mickey BC Koh	Infection and Immunity Clinical Academic Group, St George's, University of London; Department of Haematology, St George's University Hospital NHS Foundation Trust, London SW17 0QT
Daniele Avenoso	King's College Hospital NHS Foundation Trust, London, SE5 9RS, UK
Robin Sanderson	
Claire Taylor	Leeds Institute of Medical Research, University of Leeds, Leeds, LS2 9NL
Khushpreet Bhandal	Liver Research Delivery Team, University Hospitals Birmingham NHS Foundation Trust, Birmingham, B15 2GW, UK
Diana Hall	
Andrew Filer	National Institute for Health Research (NIHR) Birmingham Biomedical Research Centre and NIHR Clinical Research Facility, Institute of Inflammation and Ageing, University of Birmingham and University Hospitals Birmingham NHS Foundation Trust, Birmingham, B15 2TT, UK
Palak Trivedi	
Gordon Cook	

Erin Hurst<br>Amy Publicover<br>Katy Scouse	Northern Centre for Cancer Care, Freeman Hospital, Newcastle upon Tyne, NE7 7DN, UK
Paul Klenerman<br>Susanna J Dunachie,<br>Eleanor Barnes	Nuffield Department of Medicine, University of Oxford, Oxford, OX1 2JD, UK.<br>National Institute for Health Research, Oxford Biomedical Research Centre, Oxford University Hospitals NHS Trust, Oxford, UK.
Sam M Murray<br>Zixiang Lim<br>Jack Satsangi<br>Sophie Irwin<br>Georgina Meacham<br>Thomas Marjot<br>Stavros Dimitriadis<br>Jem Chalk<br>Daniel Hanke<br>Josef Hanke<br>Saoirse Healy<br>Stephen Laidlaw<br>Stephanie Longet<br>Nicholas Provine<br>Sarah Thomas<br>Victoria Walker<br>Zay Win	Nuffield Department of Medicine, University of Oxford, Oxford, OX1 2JD, UK.
Richard Beesley<br>Vicky Churchill<br>Holly Loughton<br>Elspeth Insch<br>Eilean MacDonald	Patient and Public Representatives on the Trial Management Group
Doreen Trown	Sheffield Teaching Hospitals NHS Foundation Trust, Royal Hallamshire Hospital, Sheffield S10 2JF, UK
Patricia Faria<br>Julie Chackathayil	St George's hospital and Medical School, St George's University Hospitals NHS Foundation Trust, London, SW17 0QT, UK
Clare Hutchison<br>Deborah Richardson	University Hospital Southampton NHS Foundation Trust, Southampton General Hospital, Southampton, SO16 6YD, UK
Maxine Arnott<br>Louise Bennett<br>James Brock<br>Victora Keillor<br>Andrew Melville<br>Lisa Melville<br>Samantha Miller<br>Aurelie Najm<br>Caron Paterson<br>Lewis Rodgers<br>Matthew Rutherford<br>Suzann Rundell<br>Emily Smith<br>Lynn Stewart<br>Flavia Sunzini	University of Glasgow, Glasgow, G12 8QQ, UK
Miles Carroll	Wellcome Centre for Human Genetics, University of Oxford, Oxford, UK.

EASL supported COVID-Hep vaccine network

Patrizia Burra	University of Padova, Department of Surgery, Oncology and Gastroenterology DISCOG, Italy
Francesco Paolo Russo	
Paola Zanaga	
Thomas Marjot	Oxford Centre for Diabetes, Endocrinology and Metabolism (OCDEM), NIHR Oxford Biomedical Research Centre, Churchill Hospital, University of Oxford, Oxford, UK
Sam M. Murray	Nuffield Department of Medicine, University of Oxford, Oxford, OX1 2JD, UK.
Eleanor Barnes	
Victoria Walker	
Anthony Brown	
Georgina Meacham	
Sophie Irwin	
Marc Lütgehetmann	Institute of Medical Microbiology, Virology and Hygiene, University Medical Center Hamburg-Eppendorf, Hamburg, Germany
Melanie Wittner	Department of Internal Medicine, University Medical Center Hamburg-Eppendorf, Hamburg, Germany
Golda Schaub	
Paul Duengelhoef	
Martina Sterneck	
Ansgar W. Lohse	
Julian Schulze zur Wiesch	
Pietro Lampertico	Division of Gastroenterology and Hepatology, Foundation IRCCS Ca' Granda Ospedale Maggiore Policlinico, Milan, Italy
Massimo Iavarone	
Jonathan Cook	Centre for Statistics in Medicine, University of Oxford, Oxford, UK
Maria-Carlota Londoño	Liver Unit, Hospital Clínic, Institut de Investigacions Biomèdiques August Pi i Sunyer (IDIBAPS), University of Barcelona, Barcelona. CIBEREHD (Centro de Investigación Biomédica en Red Enfermedades Hepáticas y Digestivas). Health Care Provider of the European Reference Network on Rare Liver Disorders (ERN-Liver), Spain
Elisa Pose	
Valeria Perez	
Virginia Hernandez-Gea	
Juan Carlos Garcia-Pagan	
Pere Gines	
Nicola van Berckel	

PITCH Consortium

Thushan I. de Silva	Department of Infection, Immunity and Cardiovascular Disease, University of Sheffield, Sheffield, UK
Sarah L. Rowland-Jones	
Sian Faustini	Institute for Immunology and Immunotherapy, College of Medical and Dental Science, University of Birmingham, Birmingham, UK
Alex Richter	
Susan L Dobson	Institute of Infection, Veterinary and Ecological Sciences, University of Liverpool, Liverpool, UK
Shona C Moore	
Lance Turtle	
Daniel G. Wootton	
James E.D. Thaventhiran	MRC Toxicology Unit
Donal Skelly	Nuffield Department of Clinical Neurosciences, University of Oxford, Oxford, UK
Priyanka Abraham	Nuffield Department of Medicine, University of Oxford, Oxford, UK
Sandra Adele	
Mohammad Ali	
Eleanor Barnes	
Anthony Brown	
Miles Carroll	
Christopher P. Conlon	
Alexandra S Deeks	
Susanna Dunachie	
John Frater	
Lisa Frending	
Siobhan Gardiner	
Anni Jamsen	
Paul Klenerman	
Barbara Kronsteiner	
Stephanie Longet	
Tom Malone	
Alexander J. Mentzer	
Eloise Phillips	
Patpong Rongkard	
Lizzie Stafford	
Sagida Bibi	Oxford Vaccine Group, Department of Paediatrics, University of Oxford, Oxford, UK
Christina Dold	
Teresa Lambe	
Katie Jeffery	Radcliffe Department of Medicine, University of Oxford, Oxford, UK
Christopher JA Duncan	Translational and Clinical Research Institute, Newcastle University, Newcastle-upon-Tyne
Rebecca P. Payne	
Simon Travis	Translational Gastroenterology Unit, University of Oxford, Oxford, UK
Sarah Foulkes	UK Health Security Agency, UK
Victoria Hall	
Susan Hopkins	
Jasmin Islam	
Ashley Otter	

Supplementary methods

Ethical and regulatory approvals

All centres involved in the EASL supported COVID-Hep vaccine registry recruited participants through local ethics approvals as follows: University Medical Center Hamburg-Eppendorf (approved by local ethics committee, Hamburg, Germany) ref No. PV7103 and PV7298; Foundation IRCCS Ca' Granda Ospedale Maggiore Policlinico: part of PolImmuneCOVID study (No. 286_2021) approved by INMI "Lazzaro Spallanzani" Ethics Committee (Roma, Italy); University of Padova: URC code COVID16; University of Barcelona: Reg. No. HCB/2021/0632 approved by Comité de ética e investigación médica (CEIM). The UK OCTAVE study was approved by the UK Medicines and Healthcare Products Regulatory Agency and London and Chelsea Research Ethics Committee (REC reference: 21/HRA/0489). The PITCH study is a sub-study of the SIREN study, which was approved by the Berkshire Research Ethics Committee, Health Research 250 Authority (REC reference: 20/SC/0230). All PBMCs collected at participating sites that were centralised to the University of Oxford were transferred and stored in accordance with the UK Human Tissue Act.

Anti-SARS-CoV-2 VoC IgG binding and ACE2 inhibition

In order to assess antibody responses to VoC, IgG titres to the spike protein of wild-type SARS-CoV-2 and nine of the most prevalent Omicron subvariants (as of February 2023: B.1.1.529/BA.1/BA.1.15, BA.2.75, BA.2.75.2, BA.4.6, BA.5, BF.7, BQ.1, BQ.1.1, and XBB.1) were assessed using a multiplexed MSD® immunoassay (K15668U). In brief, antigens were spotted at 200−400 µg/mL in 96-well plates which were blocked with MSD® Blocker A for 30 minutes. Following washing plasma/serum samples were diluted 1:10,000 and 1:30,000 in diluent buffer and incubated for 2 hours. Samples were then washed and detected using a MESO® SECTOR S 600 Reader. Concentrations were expressed in Units/ml (U/mL).

To assess functional antibody responses, a V-PLEX SARS-CoV-2 Panel 33 (ACE2) Kit (K15679U) was used to measure the ability of serum/plasma samples to inhibit angiotensin-converting enzyme 2 (ACE2) binding to the RBD of wild-type and the same Omicron subvariants listed above. Assays were performed as per manufacturer's instructions with 1:10 and 1:100 dilutions of serum/plasma. Percentage ACE2 inhibition was determined by comparison of chemiluminescence of sample spots compared to negative controls (blanks) on each plate.

IFNγ T-cell ELISpot assay

200,000 thawed PBMCs were rested for 3 hours and added to Multiscreen-IP filter plates (Millipore) coated with capture antibody (clone 1-D1K). Overlapping peptide pools (18-mers with 10 amino acid overlap, Mimotopes) representing wild-type S1 and S2 regions, membrane, and N proteins were added at a final concentration of 2ug/ml for 16–18hrs at 37°C. Selected samples also included pools covering

the entire Omicron (B.1.1.529, BA.1) S1 and S2 regions, and pools including only peptides which contained mutations in BA.1 spike, or the analogous peptides from wild-type (minipools). CEF and concanavalin A were used as positive controls, DMSO in Rab10 was used as a negative control. Plates were developed and then read using a CTL immunocapture (Cellular Technology Limited) plate reader, using Smartcount® settings. Mean spots from DMSO negatives are removed from stimulation to give antigen-specific responses. A positive IFNγ response was defined as mean DMSO + 2 standard deviations.

Breakthrough SARS-CoV-2 infection after COVID-19 vaccination

Rates of breakthrough infection were plotted over time for each of the 4 recruiting countries alongside the corresponding proportions of circulating viral variants. Country-specific proportions of SARS-CoV-2 variants were calculated based on data shared via GISAID [1] EpiCoV database, downloaded 23 March 2023. The date on which Omicron became the dominant variant (defined as representing >90% of circulating variants) was 1st January 2022 in the UK, 16th January 2022 in Italy and Spain, and 23rd January 2022 in Germany.

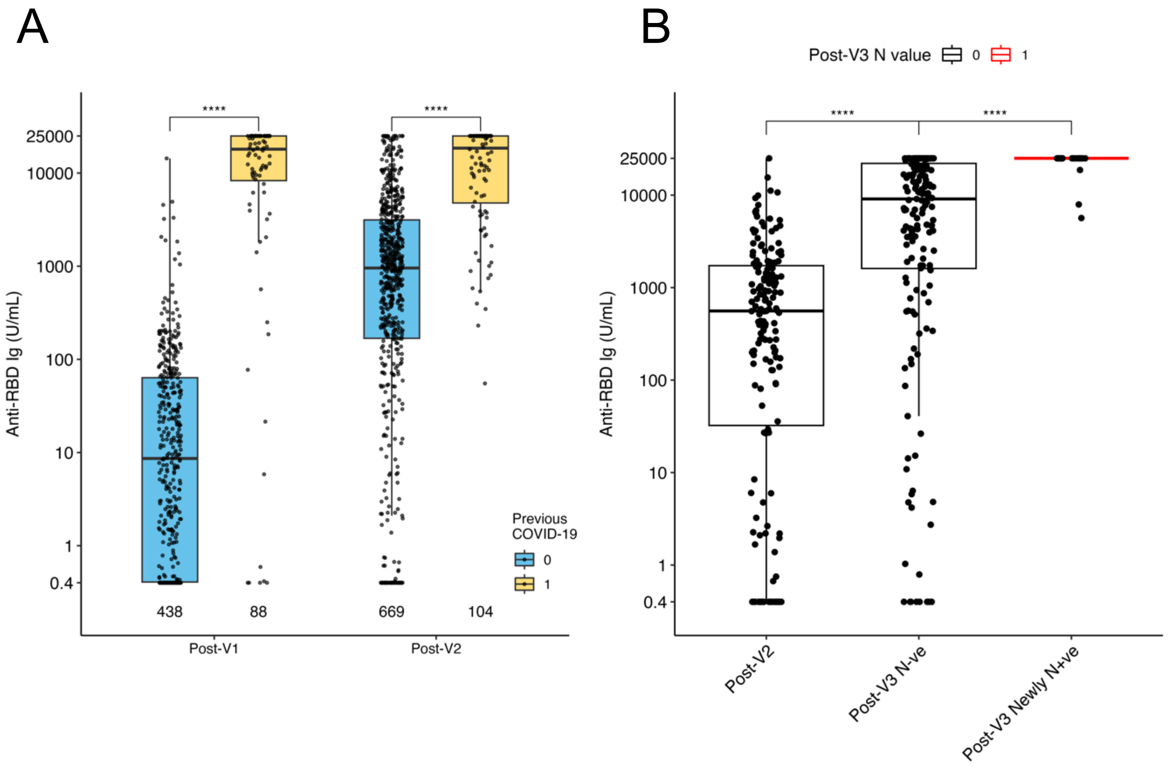

Fig. S1. A) Magnitude of anti SARS-CoV-2 RBD Ig in infection naïve and previously SARS-CoV-2 infected individuals at post-V1 and post-V2 timepoints. B) Magnitude of anti SARS-CoV-2 RBD Ig in Naïve individuals at post-V2 and post-V3 timepoints and in individuals who became nucleocapsid positive between second and third vaccines (Post-V3 Newly N+ve). Boxes represent median and IQR, whiskers represent +/- 1.5x IQR. Mann Whitney U test used, adjusted P value presented.

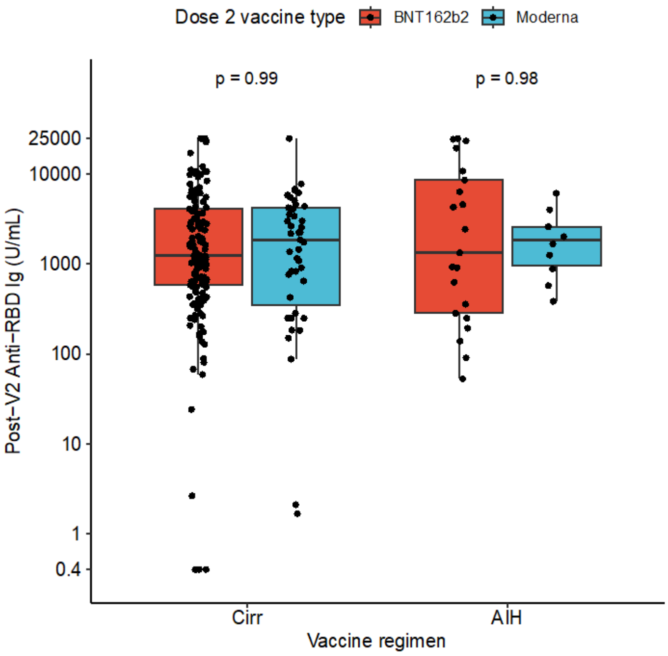

Fig. S2. Magnitude of anti-SARS-CoV-2 RBD Ig in BNT162b2 and mRNA=1273 (Moderna) vaccinated SARS-CoV-2 infection naive individuals from the cirrhosis and autoimmune hepatitis (AIH) disease groups at the post-V2 timepoint. Boxes represent median and IQR, whiskers represent +/- 1.5x IQR. Mann-Whitney U test used, adjusted P value presented.

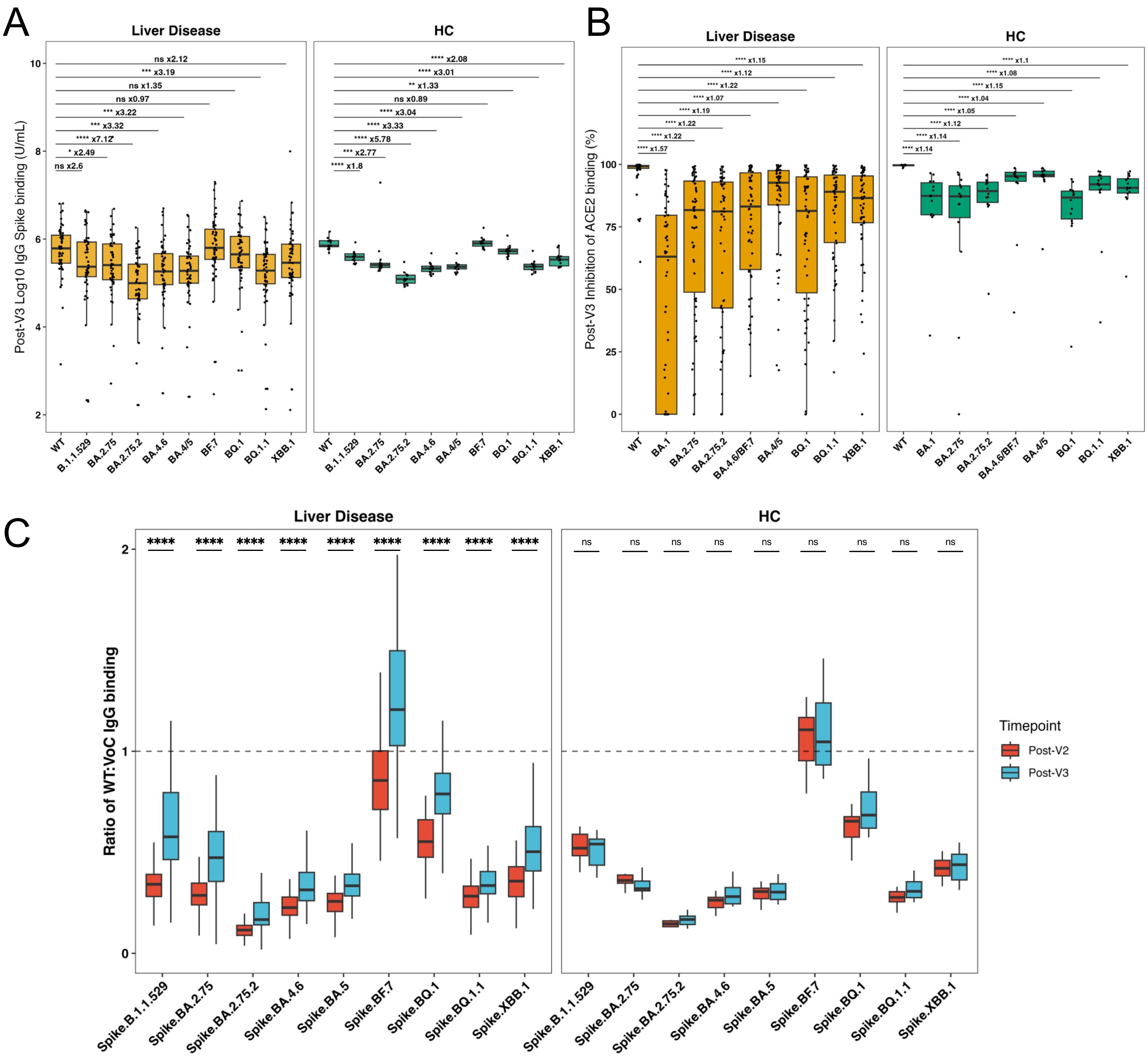

Fig. S3. Post-v3 IgG (A) and ACE2 inhibition (B) to SARS-CoV-2 VoC, separated by liver disease versus healthy controls. C) Ratio of IgG binding to WT and each respective VoC at post-v2 and post-v3 timepoints in liver disease and HC. Two-sided Mann-Whitney U test adjusted with Holm-Bonferroni. Fold-change of median depicted. Boxes represent median and IQR, whiskers represent +/- 1.5x IQR. HC = healthy controls; ACE2 = angiotensin-converting enzyme 2, WT = wild-type. * = P<0.05, ** = P<0.01, *** = P<0.001, **** = P < 0.0001.

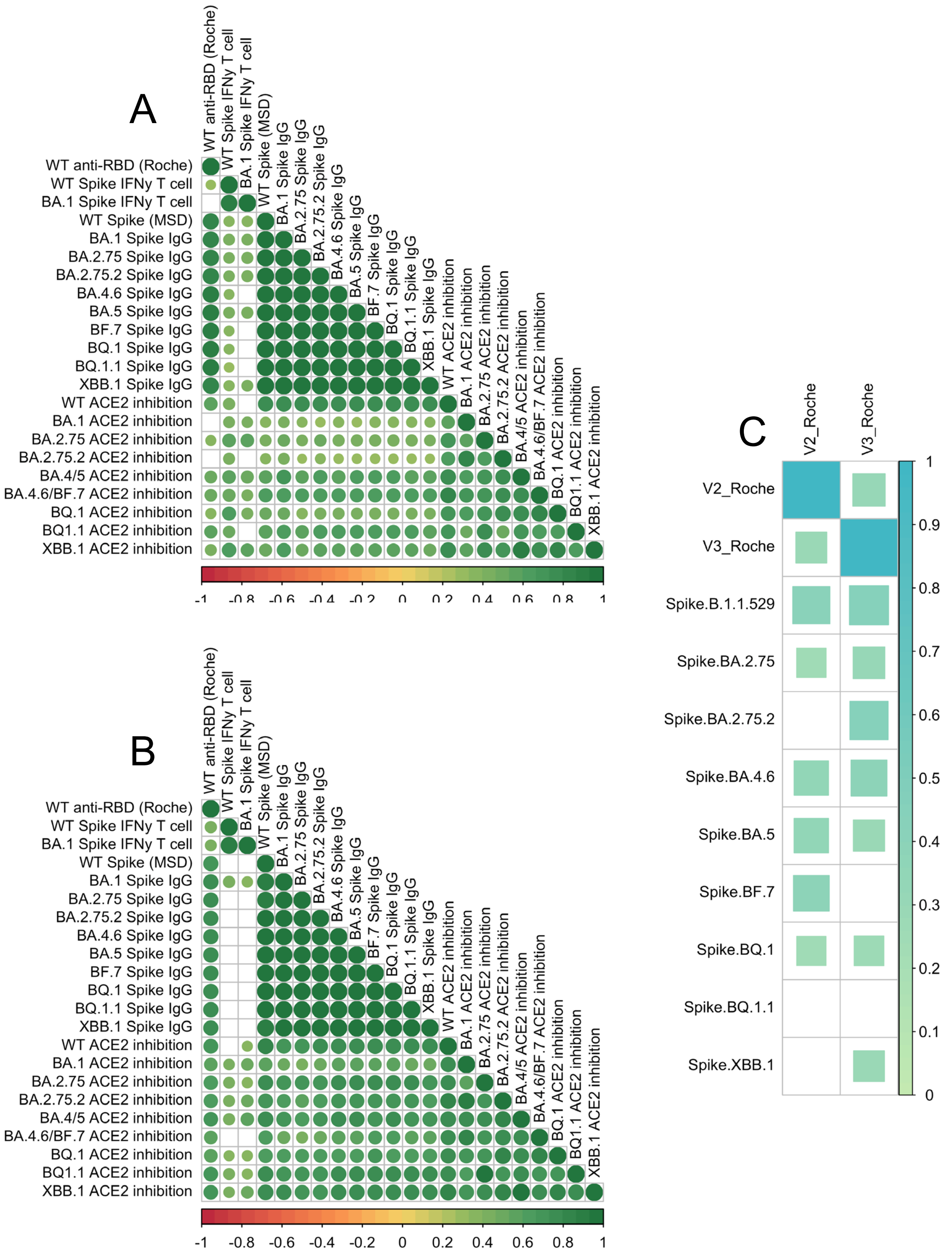

Fig. S4. Correlation of immune assays at A) post-v2 and B) post-v3 timepoints. C) correlation of WT anti-RBD Ig with ratios of WT:VoC binding at post-V2 and post-V3 timepoint. Only significant correlations (P<0.05) are shown. Spearman's correlation. Size and shade of spots/squares represent r value**.** V2_Roche = post-V2 anti-RBD Ig; V3_Roche = post-V3 anti-RBD Ig

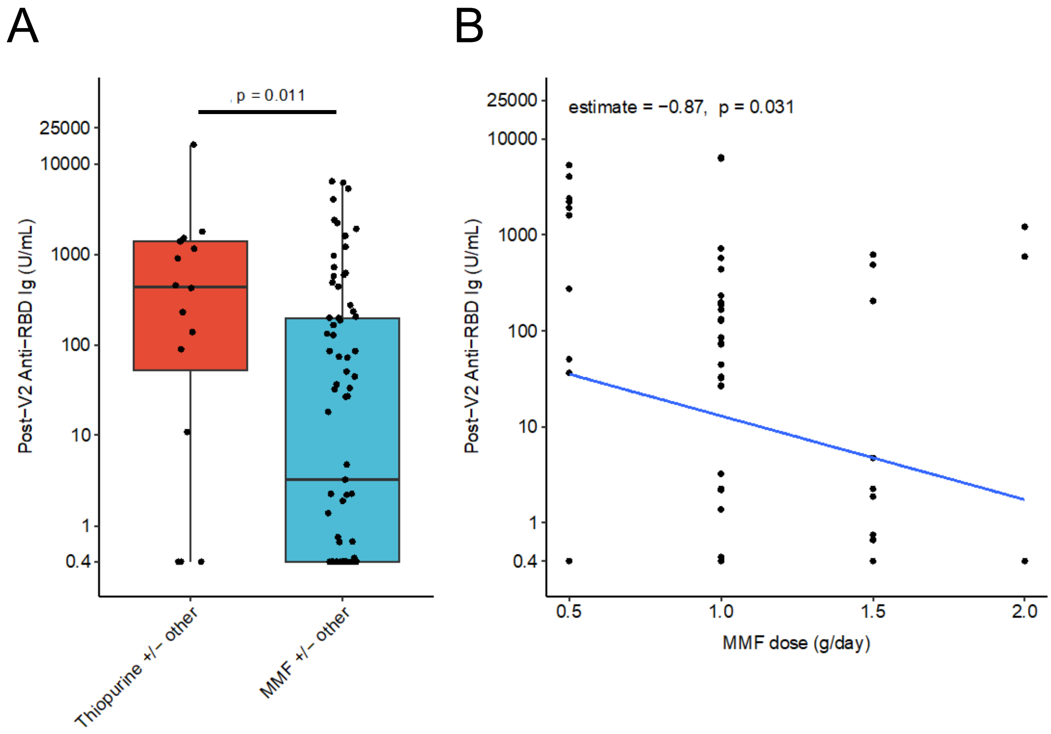

Fig. S5. A.) Anti-RBD Ig responses at the post-V2 timepoint in SARS-CoV-2 infection naive LT recipients who received either thiopurine (Azathioprine or 6-mercaptopurine) or Mycophenolate mofetil (MMF) as an immunosuppressive therapeutic. Participants may have received other immunosuppressive therapeutics in addition. B) MMF dose breakdown (gram/day) in SARS-CoV-2 infection naïve LT recipients at post-V2 timepoint. Linear model of log10 transformed Anti-RBD Ig compared with daily MMF dose. Line represents linear fit, shading represents 95% confidence interval. Boxes represent median and IQR, whiskers represent +/- 1.5x IQR. A) Statistical comparison with Mann-Whitney U test.

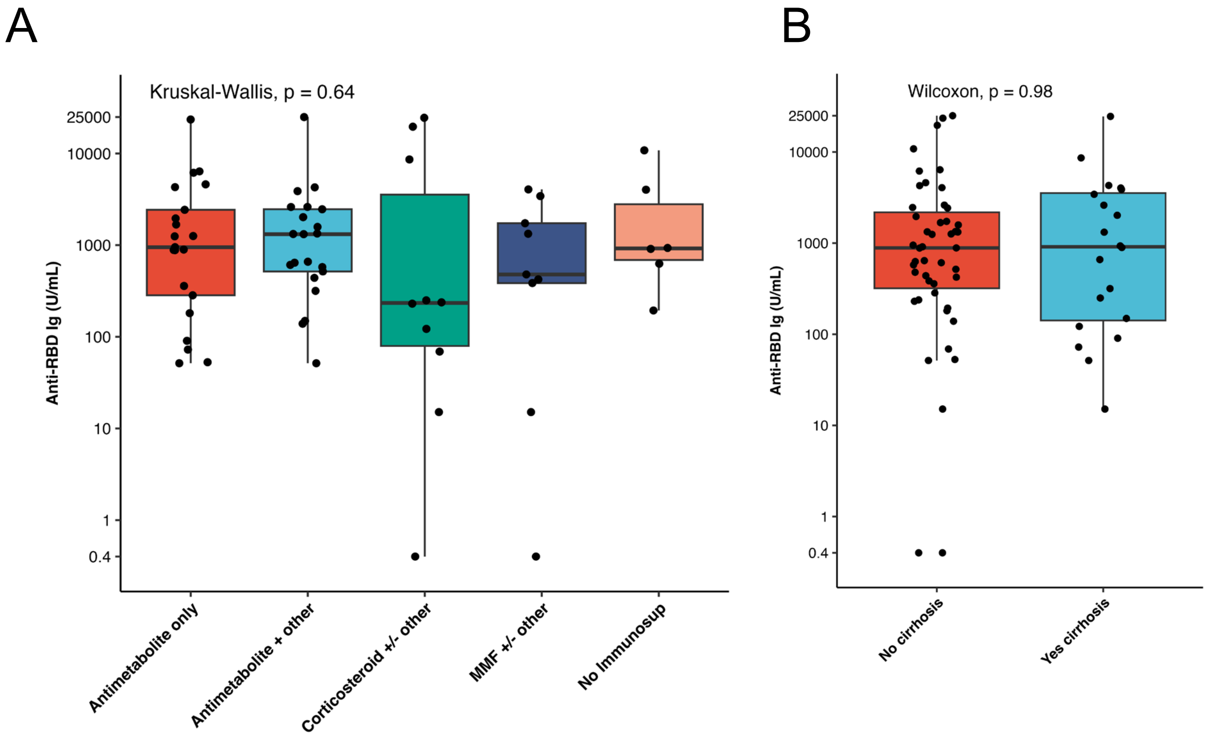

Fig. S6. SARS-CoV-2 infection naïve patients with autoimmune hepatitis at post-v2 timepoint, comparing A) immunosuppressive therapies and B) presence of cirrhosis. Boxes represent median and IQR, whiskers represent +/- 1.5x IQR. Kruskal Wallis (A) or Two-sided Mann-Whitney U test (B). Antimetabolites include 6-mercaptopurine and azathioprine. MMF = Mycophenolate mofetil.

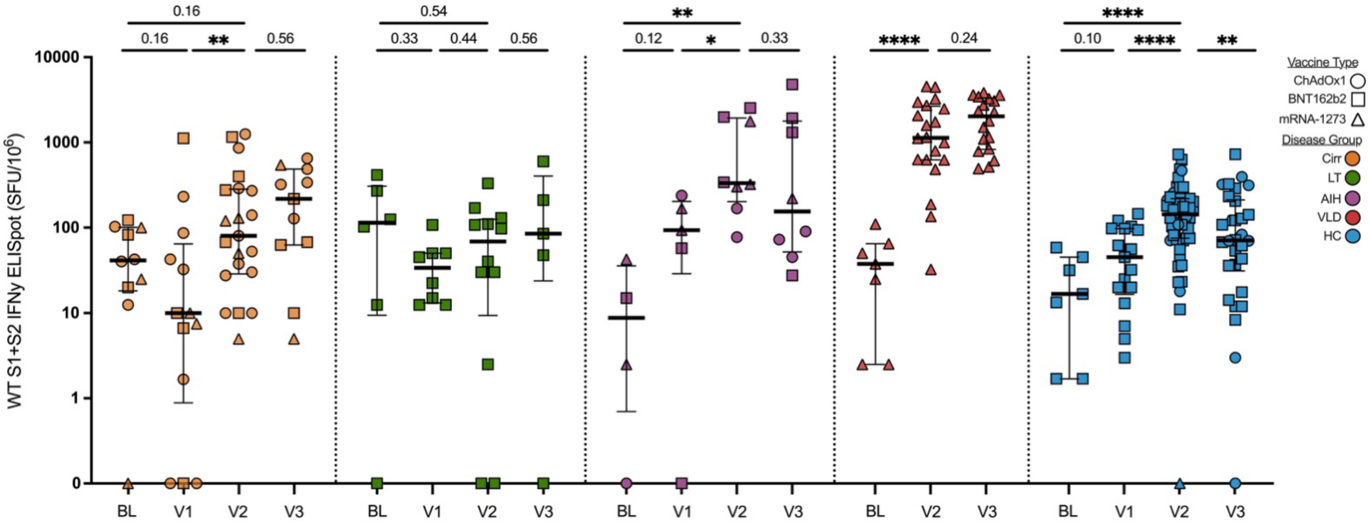

Fig. S7. As in Figure 5A, magnitude of IFN-γ T cell response to wild-type SARS-CoV-2 spike peptides across time in a subgroup of SARS-CoV-2 naïve people with cirrhosis (Cirr, n = 24), liver transplant recipients (LT, n = 12), autoimmune hepatitis (AIH, n=12), vascular liver disease (VLD, n = 22) and healthy controls (HC, n = 28). Baseline data are from same individuals later timepoints. Vaccine type indicated by point of shape. Mann-Whitney U test.

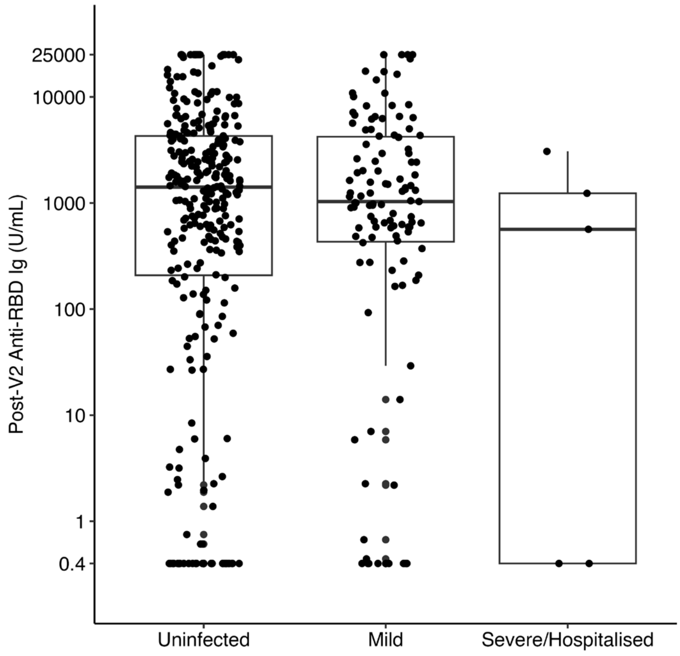

Fig. S8. Anti-RBD Ig at post-v2 timepoint in individuals who did not get breakthrough SARS-CoV-2 infection, had mild-moderate breakthrough SARS-CoV-2 infection or had severe SARS-CoV-2 breakthrough infection after vaccination.

		Baseline		Post-V1			Post-V2			Pre-V3			Post-V3			
Vaccine Type	Disease group	N	Median (IQR)	N	Median (IQR)	P val[1]	N	Median (IQR)	Pval[2]	N	Median (IQR)	P val[3]	N	Median (IQR)	P val[4]	P val[5]
ChAdOx1	Cirr	15	0.4 (0.4-0.4)	34	19.6 (3.3-37.1)	1	85	1106 (395-1838)	7.2×10^{-11}	4	637 (281-6956)	1	6	15368 (11784-22791)	1	0.3
	AIH	6	0.4 (0.4-0.4)	20	15.1 (1.6-41.9)	1	34	498 (129-1323)	4.3×10^{-4}	2	312 (237-387)	1	3	15401 (11133-20201)	1	1
	LT	6	0.4 (0.4-0.4)	27	0.4 (0.4-19.0)	1	52	63 (0.4-608)	0.1	2	151 (133-170)	1	2	3133 (1572-4694)	1	1
	HC	-	-	10	187.5 (87.0-392)	N/A	20	1198 (855-1546)	0.02	-	-	N/A	-	-	N/A	N/A
mRNA	Cirr	156	0.4 (0.4-0.4)	153	13.3 (2.5-55.6)	9.2×10^{-11}	204	1413.5 (578-4140)	6.6×10^{-23}	52	476 (214-958)	0.35	102	18015 (5927-25000)	1.5×10^{-8}	2.2×10^{-6}
	AIH	19	0.4 (0.4-0.4)	19	16.6 (1.8-71.6)	0.87	33	1341 (577-4605)	0.002	11	815 (316-2035)	1	10	18280.5 (13784-25000)	0.73	1
	LT	117	0.4 (0.4-0.4)	119	0.4 (0.4-1.6)	0.09	179	114 (0.4-1113)	2.9×10^{-13}	105	92 (7-390)	1	95	1861 (34-13534)	0.003	9.4×10^{-5}
	VLD	30	0.4 (0.4-0.4)	29	147 (116-203)	0.10	27	4880 (3203-7663)	4.7×10^{-4}	25	1943 (1441-2907)	1	24	25000 (25000-25000)	0.002	0.1
	HC	-	-	27	98.7 (57.2-148)	N/A	35	15634 (10829-21445)	3.2×10^{-11}	22	2171 (1584-3416)	0.007	23	25000 (18359-25000)	2.1×10^{-5}	1

Table S1: In SARS-CoV-2 infection naïve individuals, Roche anti-RBD antibody response to one, two and three doses of COVID-19 vaccine, separated by vaccine type and disease group. [1] comparison of Baseline and Pre-V2, [2] comparison of Pre-V2 and Post-V2, [3] comparison of Post-V2 and Pre-V3, [4] comparison of Pre-V3 and Post-V3, [4] comparison of Post-V2 and Post-V3. Kruskal Wallis with Dunn's post-hoc test, adjusted for multiple comparisons using Bonferroni correction. Cir = Cirrhosis, AIH = Autoimmune hepatitis, LT = Liver transplant, Az = AstraZeneca vaccine.

	Univariable		Multivariable	
**Variable**	**Estimate (95% CI)**	**P value**	**Estimate (95% CI)**	**P value**
Age 45-64	-0.6 (-0.8 - -0.3)	4.81E-06	-0.3 (-0.6 - -0.1)	0.0034
Age 65-74	-0.9 (-1.2 - -0.7)	5.22E-11	-0.5 (-0.8 - -0.3)	8.91E-05
Age 75+	-1 (-1.4 - -0.7)	1.38E-07	-0.6 (-0.9 - -0.2)	0.001
Male sex	-0.3 (-0.5 - -0.1)	0.0030	-0.1 (-0.3 - 0)	0.12
Obesity	0.2 (-0.1 - 0.4)	0.17	-	NA
Hypertension yes	-0.3 (-0.5 - -0.1)	0.013	0 (-0.2 - 0.1)	0.75
Current smoker	0.2 (-0.1 - 0.5)	0.21	-0.1 (-0.4 - 0.1)	0.33
Previous smoker	0.2 (-0.1 - 0.4)	0.16	0.1 (-0.1 - 0.3)	0.29
AIH	-1 (-1.3 - -0.6)	6.81E-08	-0.3 (-0.7 - 0)	0.084
Cirr	-0.7 (-1 - -0.5)	5.01E-08	-0.1 (-0.4 - 0.2)	0.50
LT	-2.1 (-2.4 - -1.8)	1.11E-46	-1.4 (-1.7 - -1.1)	6.18E-18
VLD	-0.2 (-0.7 - 0.2)	0.33	0.2 (-0.3 - 0.6)	0.50
mRNA vaccine	0.3 (0.1 - 0.5)	0.011	0.4 (0.2 - 0.6)	3.39E-06
Heterologous vaccine	1 (0.3 - 1.7)	0.0074	1.3 (0.8 - 1.9)	6.18E-06
Previous COVID-19	1.4 (1.1 - 1.6)	3.43E-23	1 (0.8 - 1.2)	1.47E-16

Table S2: Linear regression model of post-v2 log10 transformed anti-RBD Ig across entire cohort. Age variable is compared to 18-44year old age group. * indicates significant values (P<0.05)

	Comparison groups		Timepoints	
	Group 1	Group 2	Post-V2 (p val)	Post-V3 (p val)
AstraZeneca	Cirr	AIH	0.06	-
	Cirr	LT	2.00E-08*	-
	Cirr	HC	0.57	-
	AIH	LT	0.01*	-
	AIH	HC	0.06	-
	LT	HC	0.000017*	-
mRNA	Cirr	AIH	0.69	0.50999
	Cirr	LT	2.00E-15*	1.98E-08*
	Cirr	VLD	0.0039*	0.00031*
	Cirr	HC	1.30E-10*	0.06007
	AIH	LT	3.40E-06*	0.00219*
	AIH	VLD	0.05	0.12800
	AIH	HC	5.96E-06*	0.57498
	LT	VLD	5.90E-12*	9.10E-13*
	LT	HC	1.28E-27*	1.6641E-07*
	VLD	HC	0.03*	0.23352

Table S3: In infection naïve individuals, comparison of Roche anti-RBD antibody response across disease groups at post-V2 and post-V3. Comparisons at post-v3 in AstraZeneca vaccinated individuals not made due to low n numbers. Kruskal Wallis with Dunn's post-hoc test, adjusted for multiple comparisons using Benjamini Hochberg. Cir = Cirrhosis, AIH = Autoimmune hepatitis, LT = Liver transplant, Az = AstraZeneca vaccine. * indicates statistical significance (P<0.05)

Disease group	Vaccine platform	Timepoint	Seronegative (%)	Seropositive (%)
Cir	ChAdOx1	Post-V1	4 (12%)	30 (88%)
		Post-V2	-	85 (100%)
		Post-V3	-	6 (100%)
	mRNA	Post-V1	20 (13%)	133 (87%)
		Post-V2	4 (2%)	200 (98%)
		Post-V3	2 (2%)	100 (98%)
LT	ChAdOx1	Post-V1	14 (52%)	13 (48%)
		Post-V2	18 (35%)	34 (65%)
		Post-V3	-	2 (100%)
	mRNA	Post-V1	80 (67%)	39 (33%)
		Post-V2	52 (29%)	127 (71%)
		Post-V3	9 (9%)	86 (91%)
AIH	ChAdOx1	Post-V1	4 (20%)	16 (80%)
		Post-V2	2 (6%)	32 (94%)
		Post-V3	-	3 (100%)
	mRNA	Post-V1	4 (21%)	15 (79%)
		Post-V2	-	33 (100%)
		Post-V3	-	10 (100%)
VLD	mRNA	Post-V1	1 (3%)	28 (97%)
		Post-V2	-	27 (100%)
		Post-V3	-	24 (100%)
HC	ChAdOx1	Post-V1	-	10 (100%)
		Post-V2	-	20 (100%)
		Post-V3	-	-
	mRNA	Post-V1	-	27 (100%)
		Post-V2	-	35 (100%)
		Post-V3	-	23 (100%)

Table S4: In infection naïve individuals, comparison of Roche anti-RBD antibody response rate across disease groups at post-V2 and post-V3. Seropositive defined as >0.8AU/mL by anti-RBD Ig assay. Cir = Cirrhosis, AIH = Autoimmune hepatitis, LT = Liver transplant, VLD = Vascular liver disease; HC = healthy control

	Univariable		Multivariable	
Variable	Odds Ratio (95% CI)	P value	Odds Ratio (95% CI)	P value
Age 45-64	0.61 (0.3 - 1.2)	0.17	0.36 (0.078 - 1.2)	0.14
Age 65-74	0.33 (0.16 - 0.69)	0.0035	0.19 (0.038 - 0.67)	0.017
Age 75+	0.49 (0.2 - 1.2)	0.11	0.38 (0.067 - 1.8)	0.23
Male sex	0.69 (0.44 - 1.1)	0.12	0.96 (0.48 - 1.9)	0.9
ALF	0.98 (0.4 - 2.4)	0.96	-	-
HCC	0.99 (0.54 - 1.8)	0.97	-	-
Decompensation	0.77 (0.49 - 1.2)	0.25	-	-
<2yrs post-transplant	0.52 (0.28 - 0.96)	0.038	0.43 (0.18 - 1)	0.054
mTORi only	0.83 (0.32 - 2.2)	0.7	0.49 (0.12 - 2.2)	0.32
CNI + Other	0.66 (0.38 - 1.2)	0.14	0.56 (0.23 - 1.3)	0.2
CNI + MMF	0.37 (0.22 - 0.63)	0.00029	0.42 (0.19 - 0.93)	0.036
mRNA vaccine	1.3 (0.76 - 2.2)	0.34	1.6 (0.74 - 3.3)	0.23

Table S5: Logistic regression models of anti-RBD seropositivity (>0.8U/mL) in liver transplant recipients following two doses of COVID-19 vaccine. Age is a continuous variable, all other variables are discrete. Age is compared to 18-44 age group. Previous COVID-19 was removed as a variable as 100% of patients with previous COVID-19 had responses >0.8U/mL.

	Univariable		Multivariable	
Variable	Odds Ratio (95% CI)	P value	Odds Ratio (95% CI)	P value
Age 45-64	-0.49 (-1.1 - 0.14)	0.13	-0.31 (-0.9 - 0.28)	0.31
Age 65-74	-0.62 (-1.3 - 0.08)	0.081	-0.55 (-1.2 - 0.12)	0.11
Age 75+	-0.5 (-1.5 - 0.51)	0.32	-0.81 (-1.8 - 0.14)	0.1
Antimetab. +/- other	0.16 (-0.4 - 0.72)	0.56	-0.0093 (-0.55 - 0.53)	0.97
Cirrhosis yes	0.1 (-0.39 - 0.59)	0.68	0.092 (-0.36 - 0.54)	0.69
Corticosteroid +/- other	-0.49 (-1.2 - 0.22)	0.17	-0.46 (-1.2 - 0.23)	0.2
Male sex	0.4 (-0.22 - 1)	0.2	0.36 (-0.24 - 0.97)	0.24
MMF +/- other	-0.45 (-1.2 - 0.27)	0.22	-0.34 (-1 - 0.35)	0.34
mRNA vaccine	0.83 (0.42 - 1.2)	0.00013	0.76 (0.31 - 1.2)	0.0016
No immunosupp.	0.45 (-0.32 - 1.2)	0.25	-0.47 (-1.3 - 0.33)	0.26
Previous COVID-19	1.1 (0.26 - 2)	0.011	1.1 (0.28 - 2)	0.012

Table S6: Linear regression models of log10 anti-RBD in autoimmune hepatitis patients following two COVID-19 vaccine. Age is compared to 18-44year old category. Drugs compared to antimetabolite alone group.

	Univariable		Multivariable	
Variable	Estimate (95% CI)	P value	Estimate (95% CI)	P value
Age 45-64	-0.27 (-0.64 - 0.11)	0.16	-0.37 (-0.8 - 0.069)	0.098
Age 65-74	-0.32 (-0.7 - 0.069)	0.11	-0.39 (-0.83 - 0.055)	0.086
Age 75+	-0.55 (-1 - -0.088)	0.02	-0.52 (-1 - -0.029)	0.038
Male sex	-0.032 (-0.22 - 0.16)	0.74	0.0087 (-0.19 - 0.2)	0.93
MELD	-0.064 (-0.11 - -0.02)	0.0043	-0.085 (-0.13 - -0.039)	0.00032
CP-B/C	0.042 (-0.15 - 0.23)	0.67	0.071 (-0.16 - 0.3)	0.54
ALD	0.04 (-0.14 - 0.22)	0.67	0.0091 (-0.2 - 0.22)	0.93
HBV	0.1 (-0.25 - 0.45)	0.56	0.0061 (-0.36 - 0.37)	0.97
HCV	-0.1 (-0.32 - 0.11)	0.34	-0.13 (-0.39 - 0.13)	0.33
NAFLD	0.034 (-0.17 - 0.23)	0.74	0.075 (-0.15 - 0.3)	0.5
mRNA Vaccine	0.17 (-0.026 - 0.36)	0.09	0.26 (0.05 - 0.47)	0.015
Previous COVID-19	0.88 (0.63 - 1.1)	1.90E-11	0.9 (0.65 - 1.2)	1.40E-11

Table S7: Linear regression models of log10 transformed anti-RBD antibody (>0.8U/mL) in cirrhosis patients at the Post-V2 timepoint. MELD is a continuous variable, all other variables are discrete. Age is compared to 18-44 year old group. CP = Child's Pugh class, INR = International normalized ratio, CI = Confidence interval.

Supplementary reference

[1] Elbe S, Buckland-Merrett G. Data, disease and diplomacy: GISAID's innovative contribution to global health. Global Challenges 2017;1:33-46.
